# Ruthenium-catalyzed C–H bond activation and annulation of phenothiazine-3-carbaldehydes: facile access to dual-emission materials†

**DOI:** 10.1039/d4sc07825j

**Published:** 2025-01-16

**Authors:** Junxiang Liu, Kangmin Wang, Liqiu Wan, Xianhui Yang, Bijin Li

**Affiliations:** a Chongqing Key Laboratory of Natural Product Synthesis and Drug Research, School of Pharmaceutical Sciences, Chongqing University Chongqing 401331 P. R. China bijinli@cqu.edu.cn

## Abstract

Reported herein is the first example of a ruthenium-catalyzed C–H activation/annulation of phenothiazine-3-carbaldehydes to construct structurally diverse pyrido[3,4-*c*]phenothiazin-3-iums with dual-emission characteristics. Novel organic single-molecule white-light materials based on pyrido[3,4-*c*]phenothiazin-3-iums with dual-emission and thermally activated delayed fluorescence (TADF) characteristics have been developed for the first time herein. Furthermore, the dual-emission molecule could be fabricated as water-dispersed NPs, which could be applied in two-channel emission intensity ratio imaging to observe the intercellular structure and can specifically target the cell membrane.

## Abstract 2

A ruthenium-catalyzed C–H bond activation and annulation reaction efficiently constructed the structurally diverse pyrido[3,4-*c*]phenothiazin-3-iums with anti-Kasha dual-emission and TADF characteristics.

## Graphical abstract

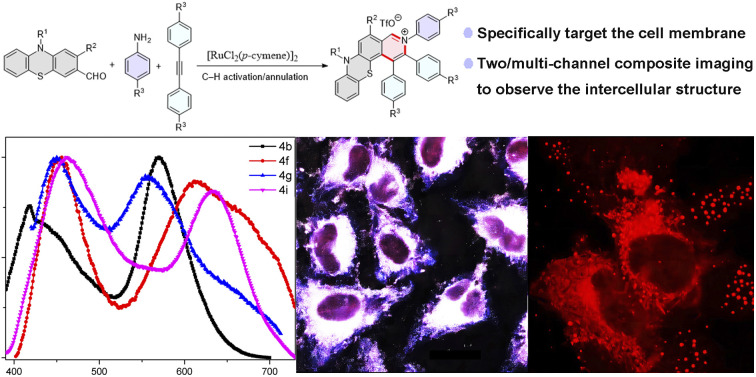

## Introduction

Dual-emission organic fluorescent materials are interesting in scientific and engineering arenas due to their excellent photophysical properties and wide range of potential applications, such as in high-sensitivity bioprobes, sensors, white-light emitters, data encryption, security systems, *etc.*^1–11^ The most common strategy to obtain dual-emission is the physical mixing of two emitting colors from different emitting centers.^12,13^ Compared with the combined two emitters, single-molecule dual-emission materials have several advantages such as good stability, long-term color balance, and low-cost fabrication.^1–9^ However, it remains a great challenge to discover single-molecule dual-emission materials due to the emission originating normally from only the lowest excited state (S_1_ or T_1_) based on Kasha's rule.^14^

**Scheme 1:**
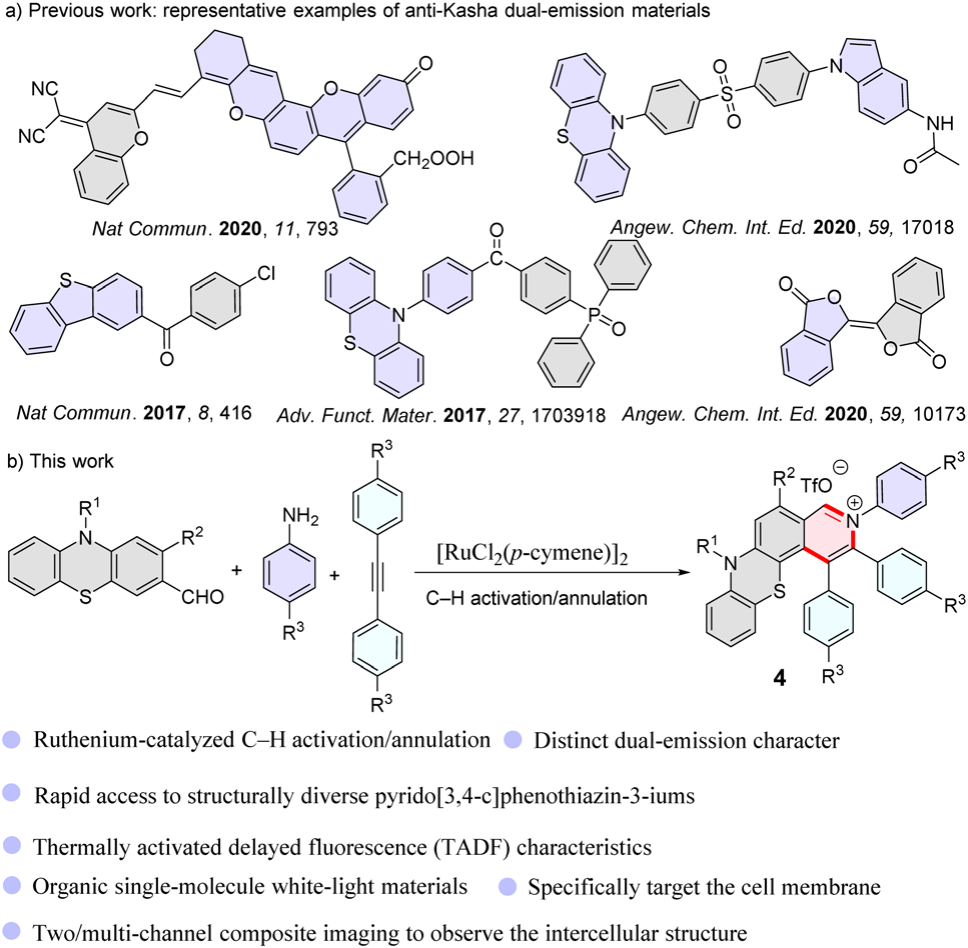
Dual-emission molecules.

Although the luminescence behavior of most organic conjugated molecules follows Kasha's rule, there are exceptions. In recent years, scientists have gradually discovered that some special organic molecules, due to their ultrafast radiative transition rates or large energy gaps between adjacent excited states, will directly undergo radiative transitions from high-energy levels.^7–10,15–33^ In recent years, Escudero and colleagues have conducted an in-depth study on anti-Kasha behaviors based on the phenomenon of high-lying excited state luminescence combined with theoretical calculations.^34–37^ It is worth mentioning that they systematically summarized anti-Kasha scenarios and creatively classified them scientifically from the perspective of electron–vibrational coupling.^34–37^ In fact, studies on anti-Kasha dual-emission materials have attracted considerable attention due to their important applications in single-molecule white-light-emitting materials and highly accurate analysis in basic life science research (Scheme 1a).^7–10,23,27^ To date, examples describing single-molecule anti-Kasha dual-emission materials have been rare^7–10,38,39^ due to the intrinsic limitation of photophysical properties of organic fluorescent molecules, and also due to the tedious multiple-step synthesis using traditional synthetic methods.

In recent years, transition metal-catalyzed C–H bond activation/annulation has been developed as a straightforward and efficient strategy for constructing organic fluorescent materials because this approach can offer more atom- and step-economic syntheses than traditional synthetic methods.^40–59^ Our recent study revealed that phenothiazine compounds are great potential candidates to prepare single-molecule dual-emission materials.^38,39^ Here, we deliberately designed a one-pot three-component reaction of phenothiazine-3-carbaldehydes, amines, and alkynes for the effective construction of single-molecule dual-emission materials *via* ruthenium-catalyzed C–H activation/annulation (Scheme 1b).

## Results and discussion

**Scheme 2:**
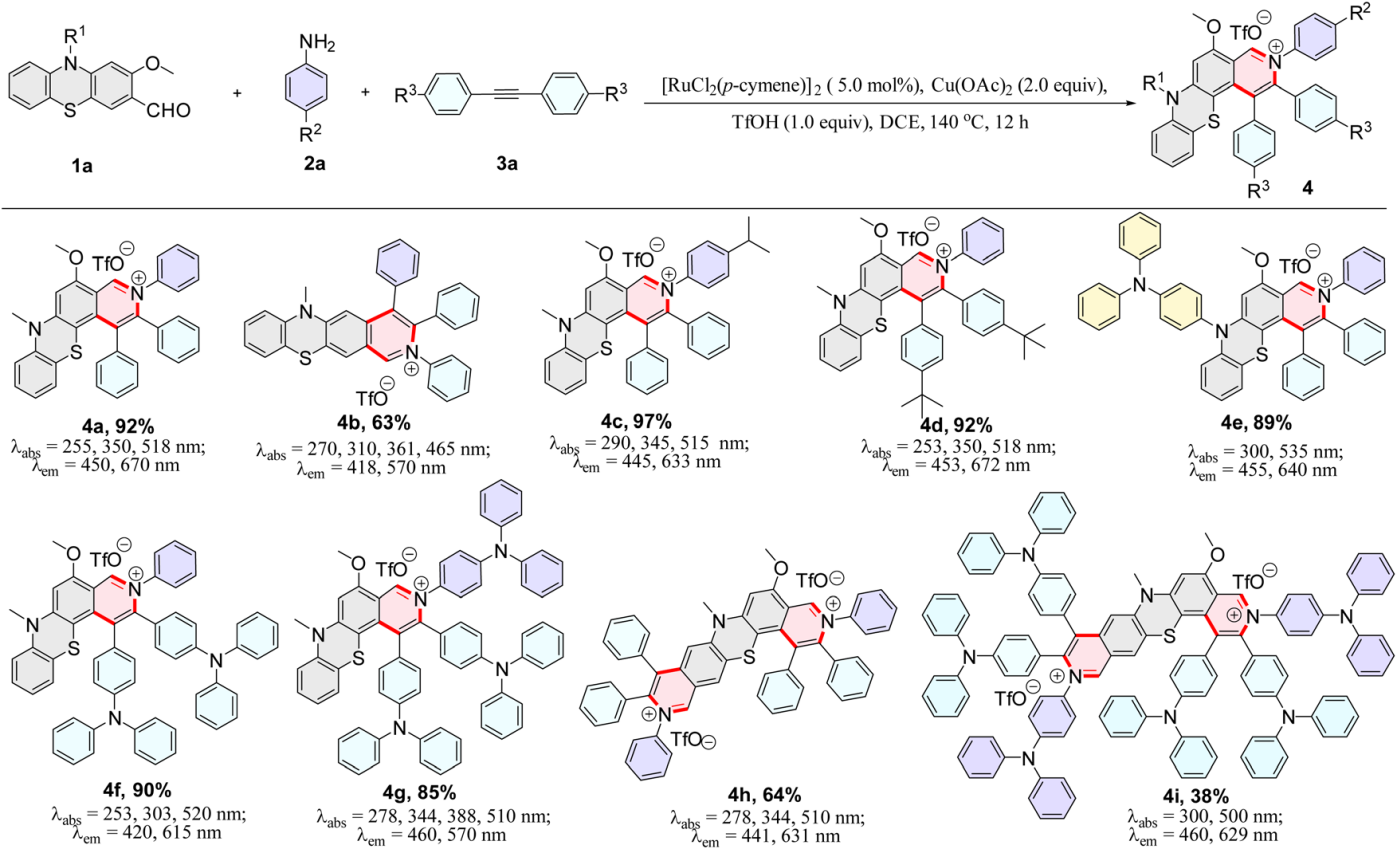
Reaction conditions: 1a (0.1 mmol, 1.0 equiv.), 2a (0.15 mmol, 1.5 equiv.), 3a (0.15 mmol, 1.5 equiv.), Cu(OAc)_2_ (0.2 mmol, 2.0 equiv.), TfOH (0.1 mmol, 1.0 equiv.), DCE (1 mL), 140 °C, 14 h. Absorptions were measured in CH_2_Cl_2_ (50 μM). Emissions were measured in CH_2_Cl_2_ (concentration: 4a, 4c, 4d: 600 μM; 4b: 0.1 μM; 4e: 800 μM; 4f: 250 μM; 4g: 500 μM; 4h: 2.5 μM; 4i: 50 μM, *λ*_ex_ = 370 nm).

We use 2-methoxy-10-methyl-10*H*-phenothiazine-3-carbaldehyde (1a), aniline (2a), and 1,2-diphenylacetylene (3a) as model substrates to evaluate the C–H bond activation and annulation in a one pot strategy (Table S1†). After screening several parameters (see ESI, Table S1†), we obtained a yield of 92% under the standard reaction conditions (5.0 mol% [RuCl_2_(*p*-cymene)]_2_, 2.0 equiv. Cu(OAc)_2_, and 1.0 equiv. TfOH, in DCE at 140 °C for 12 h) (Table S1,† entry 5). The triphenylamine (TPA) group could somewhat prevent aggregation of molecules and enhance fluorescence emission due to its propeller-like non-planar geometry and good electron-donating properties. Subsequently, a series of structurally diverse TPA/aryl-containing pyrido[3,4-*c*]phenothiazin-3-iums were synthesized under the optimized reaction conditions in good to excellent yields (Scheme 2, 4e–4g, 4i).

The structure of 4g was further confirmed by single-crystal X-ray diffraction (Fig. 2, S37 and S38†). Furthermore, a plausible catalytic cycle is proposed (see ESI, Fig. S1†). Initially, the five-membered ruthenacycle intermediate I is generated *via* imine (*in situ* generation from 1a and 2a) nitrogen coordinated with ruthenium(ii) species and *ortho*-C–H bond activation. Next, the alkyne 3a coordinates with the intermediate I to form the intermediate II, and subsequently inserts into the Ru–C bond to give the seven-membered ruthenacycle III. Finally, intermediate III undergoes reductive elimination to produce the cyclization product 4 and release Ru^I^. The Ru^II^ species is regenerated through Cu(OAc)_2_ oxidation.

**Fig. 1:**
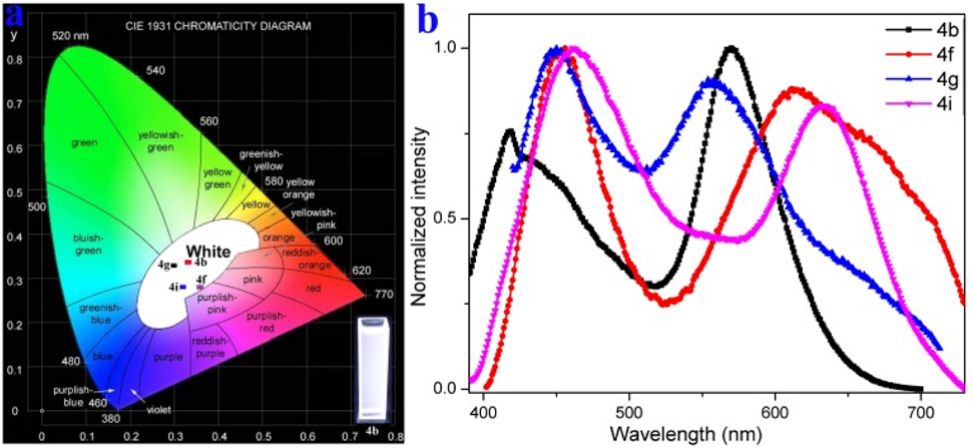
Fluorescence properties. (a) Commission Internationale de l’Eclairage (CIE) coordinates of 4b (0.33, 0.34), 4f (0.36, 0.28), 4g (0.30, 0.33), and 4i (0.32, 0.28) in CH_2_Cl_2_, respectively. (b) Fluorescence emission spectra of 4b, 4f, 4g, and 4i in CH_2_Cl_2_ (concentration: 4b: 0.1 μM; 4f: 250 μM; 4g: 500 μM; 4i: 50 μM).

The photophysical properties of 4a–4i were further measured and the corresponding dual-emission maxima are summarized in Scheme 2, Fig. S3 and S12.† Compounds 4a–4i possess a distinct dual-emission character with a relatively short blue emission and a relatively long red emission wavelength. To our delight, four pyrido[3,4-*c*]phenothiazin-3-iums exhibited white light emissions in dichloromethane solution with Commission Internationale de l’Eclairage (CIE) coordinates of 4b (0.33, 0.34), 4f (0.36, 0.28), 4g (0.30, 0.33), and 4i (0.32, 0.28) respectively at an excitation wavelength of 370 nm (Fig. 1). It is worth pointing out that the CIE coordinates of 4b is very close to those of pure white light (CIE: 0.33, 0.33). In addition, compounds 4a–4i still displayed distinct dual emissions in the PMMA film (0.1–0.2 wt%) (Fig. S13†). However, they mainly exhibit a relatively long orange to near-infrared emission in solid powders (Fig. S14†).

**Fig. 2:**
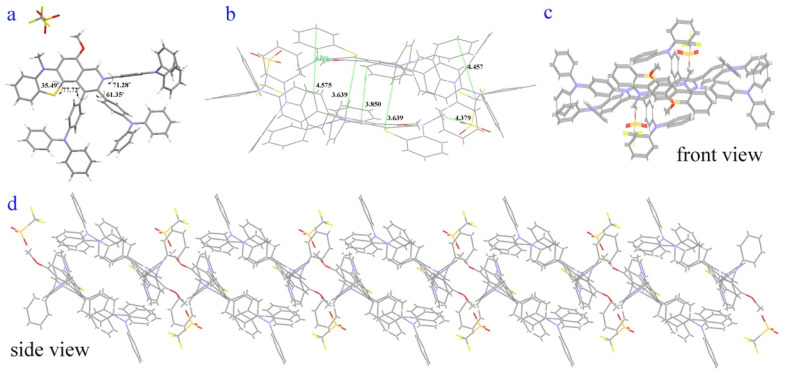
Crystal packing of 4g (CCDC-2369380). (a) The dihedral angles. (b) Two molecules are stacked in reverse. (c) The single crystal displays highly distorted molecular conformation from the front view. (d) The single crystal displays a highly rigid network structure from the side view.

Cyclic voltammetry measurements revealed that 4g has one irreversible oxidation (*E*^ox^_pa_ = 1.06 V), one reversible oxidation (*E*^ox^_1/2_ = 0.18 V *versus* Fc/Fc^+^ couple), and one reversible reduction (*E*^red^_1/2_ = −0.25 V) (Fig. S29†). Moreover, the fluorescence variation of 4g in various THF/H_2_O mixtures was surveyed and showed that 4g was aggregation-induced emission (AIE)-active (Fig. S26†). To gain insight into the AIE luminous behavior of 4g, the single crystal was further investigated (Fig. 2). The crystal of 4g displays a very weak blue emission at ∼440 nm and a strong near-infrared (NIR) fluorescence emission at 720 nm with a quantum yield of 2% (Fig. S20†). Excitation-wavelength-dependent experiments of a crystal of 4g were carried out (Fig. S20†). Lower energy excitations result in a red shift at shorter wavelengths, and longer wavelengths of light do not show significant changes (Fig. S20†). The phenothiazine skeleton shows a non-planar ‟butterfly” structure characteristic and a dihedral angle of 35.49° in the single crystal. The dihedral angles between the pyridinyl and the adjacent three phenyls reveal 71.28°, 61.35° and 77.72° (Fig. 2a). Moreover, two molecules are stacked in reverse with distances of 3.639–4.575 Å and form a cavity-shaped structure in the single crystal (Fig. 2b). The single crystal displays a highly distorted molecular conformation and rigid network structure, which are also observed from the front view and side view (Fig. 2c and d). These results demonstrate that the highly twisted conformation and hindered intermolecular π–π stacking contributed to the AIE character of 4g.

**Fig. 3:**
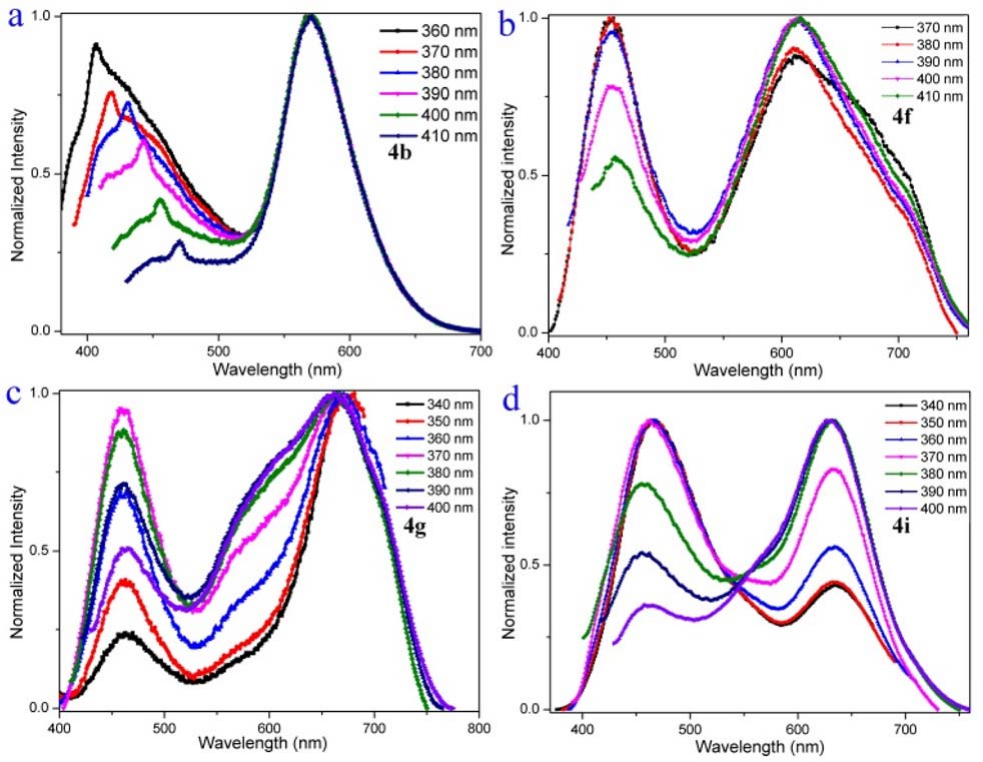
Excitation-wavelength-dependent fluorescence spectra of 4b (0.1 μM), 4f (250 μM), 4g (500 μM), and 4i (50 μM) in CH_2_Cl_2_.

To gain an in-depth understanding of the origin of the dual emission in the solution of compounds 4a–4i, emission-wavelength-dependent excitation experiments, excitation-wavelength-dependent fluorescence experiments, and time-dependent density functional theory calculations were further executed. In the emission-wavelength-dependent excitation experiments, the intensity of the longer excitation wavelengths gradually increased, and the shorter excitation wavelength peaks showed no significant changes, but there was a shoulder peak appearing as the emission wavelength increased (Fig. S4–S11†). Furthermore, the excitation-wavelength-dependent fluorescence experiments indicate that the relative intensity of the dual emission of compounds 4a–4i highly depends on the excitation wavelength (Fig. 3 and S12†). For example, lower energy excitations result in a slight red-shift and decrease the intensity at shorter wavelengths in 4b (Fig. 3a). For 4f, lower energy excitations result in a decreased intensity at shorter wavelengths and a slightly enhanced intensity at longer wavelengths at excitation wavelengths from 370 to 390 nm (Fig. 3b). For 4g, lower energy excitations result in the intensity of shorter wavelengths decreasing and then increasing (Fig. 3c). For 4i, lower energy excitations result in the intensity at shorter wavelengths decreasing and the intensity at longer wavelengths enhancing (Fig. 3d). These experimental results indicate that they have similar anti-Kasha properties, and the two fluorescence emissions bring different excited states.^8–10,34,35^

Furthermore, other cases exhibiting emission dependent on excitation rather than emission from highly excited states were further excluded. First, the compounds used for optical experiments were purified by recrystallization three times to ensure purity. The solvents used for the fluorescence test were freshly prepared after distillation and were optically pure solvents. The 2D excitation–emission plot of 4g was recorded, and two distinct emission bands with the same excitation spectra with a maximum of ∼380 nm were observed (Fig. S15†). Meanwhile, as the excitation wavelength increases, the long-wave emission still exists. Moreover, the intensity of the dual emission did not change significantly by adding trifluoroacetic acid (TFA) or the base triethylamine to the 4g solution (Fig. S16 and S17†). These experimental results indicate only the presence of one species in the 4g solution, and the protonated species can be excluded. The elemental analysis and high-performance liquid chromatography (HPLC) testing showed the presence of only one component in the system (Fig. S2†), which can exclude the presence of other species or an impurity. Second, the red-edge effect was found in rigid media and can lead to longer excitation wavelengths exciting ‘hot’ molecules, and the corresponding fluorescence emission wavelength undergoes a significant redshift. Emission wavelength as a function of the excitation wavelength of 4g in DCM indicates that there is no significant red shift in the emission peak as the excitation wavelength increases, which disagrees with the red-edge effect (Fig. S18†). Third, the fluorescence-decay experiments of 4g displayed no trace of a second lifetime in either subnanoseconds or picoseconds in DCM solvent, which excludes the presence of transition dipoles in different parts of the molecule (Fig. S19†).

**Fig. 4:**
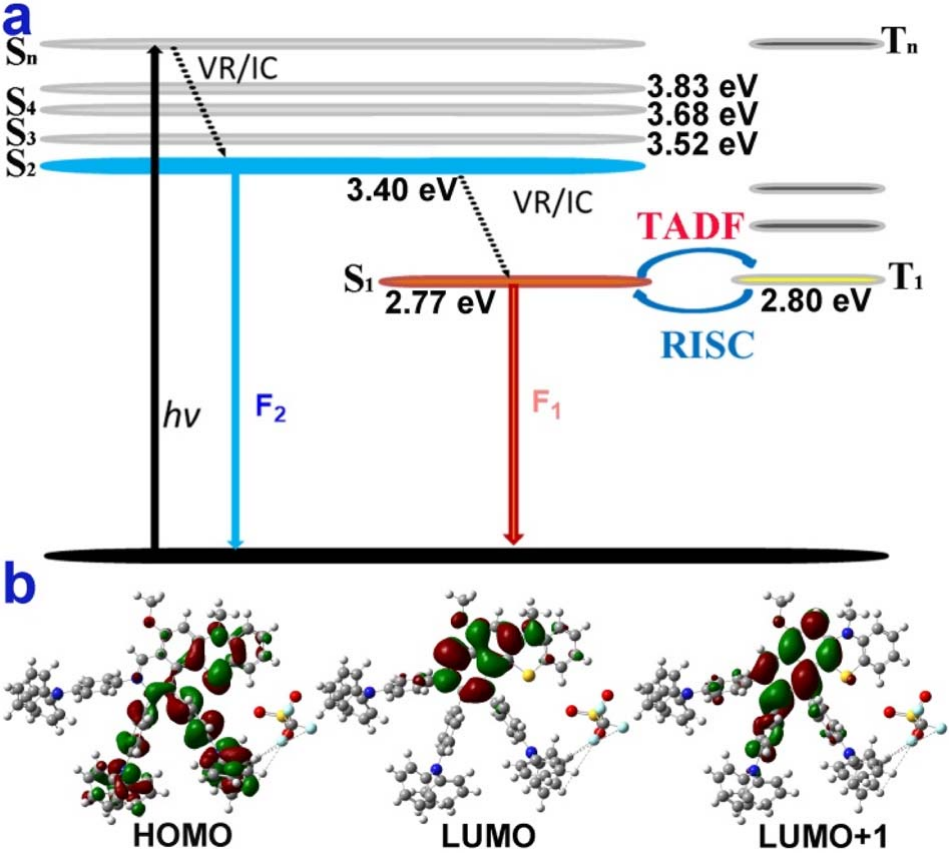
Possible luminescent mechanism of 4g. (a) Jablonski diagram illustrating the speculative dual-emission and TADF mechanism of 4g. (b) Molecular orbitals of the S_0_, S_1_, and S_2_ states of 4g.

In addition, the longer wavelengths of 4g (0.15 wt% in PMMA) enhanced intensity in the nitrogen atmosphere and decreased in the oxygen atmosphere (Fig. S28†), which shows that the enhanced part of the emission could come from reverse intersystem crossing (RISC) from T_1_ to S_1_. Moreover, microsecond-scale long-lifetime fluorescence components exist in the excited-state lifetimes of 4g films (Table S2 and Fig. S23–S25†), further indicating that 4g possesses thermally activated delayed fluorescence characteristics.^60,61^ Furthermore, the reactive oxygen species (ROS) generation efficiency of 4g was surveyed by using a typical ROS indicator (dichlorofluorescein) under UV light irradiation (Fig. S27†). The obvious fluorescence enhancement demonstrated that 4g possessed the ROS generation ability and narrow Δ*E*_S1–T1_ (Fig. 4a).

The theoretical calculations further reveal that 4g could possess similar anti-Kasha emission and TADF characters (Fig. 4, part XI in the ESI†).^8–10,15–39^ The shorter wavelength of 4g may be from high-lying singlet state emission (S_2_ → S_0_) and the longer wavelength from low-lying excited state emission (S_1_ → S_0_) (Fig. 4a). Moreover, the small energy band gaps (Δ*E*_S1–T1_) between S_1_ and T_1_ indicated that the RISC from the T_1_ to S_1_ process was highly favored (Fig. 4a). Furthermore, the highest occupied molecular orbital (HOMO) was mainly located in the electron-rich TPA group and the thiomorpholine ring (Fig. 4b). In contrast, the lowest unoccupied molecular orbital (LUMO) was distributed on the electron-withdrawing benzopyridinium moiety and part of the thiomorpholine ring (Fig. 4b). The large sufficient separation of the HOMO and LUMO could lead to a strong intramolecular charge transfer (ICT) effect and narrow Δ*E*_S1–T1_ value. Moreover, the excitation energy transfer process could take place due to there being some spectral overlap between the emission and absorption spectra of 4g.^34^ The S_1_ and S_2_ states do not fully overlap and are not separated in space either and the D and Sr index of 5.1 Å and 0.27 (part XI in the ESI†).

**Fig. 5:**
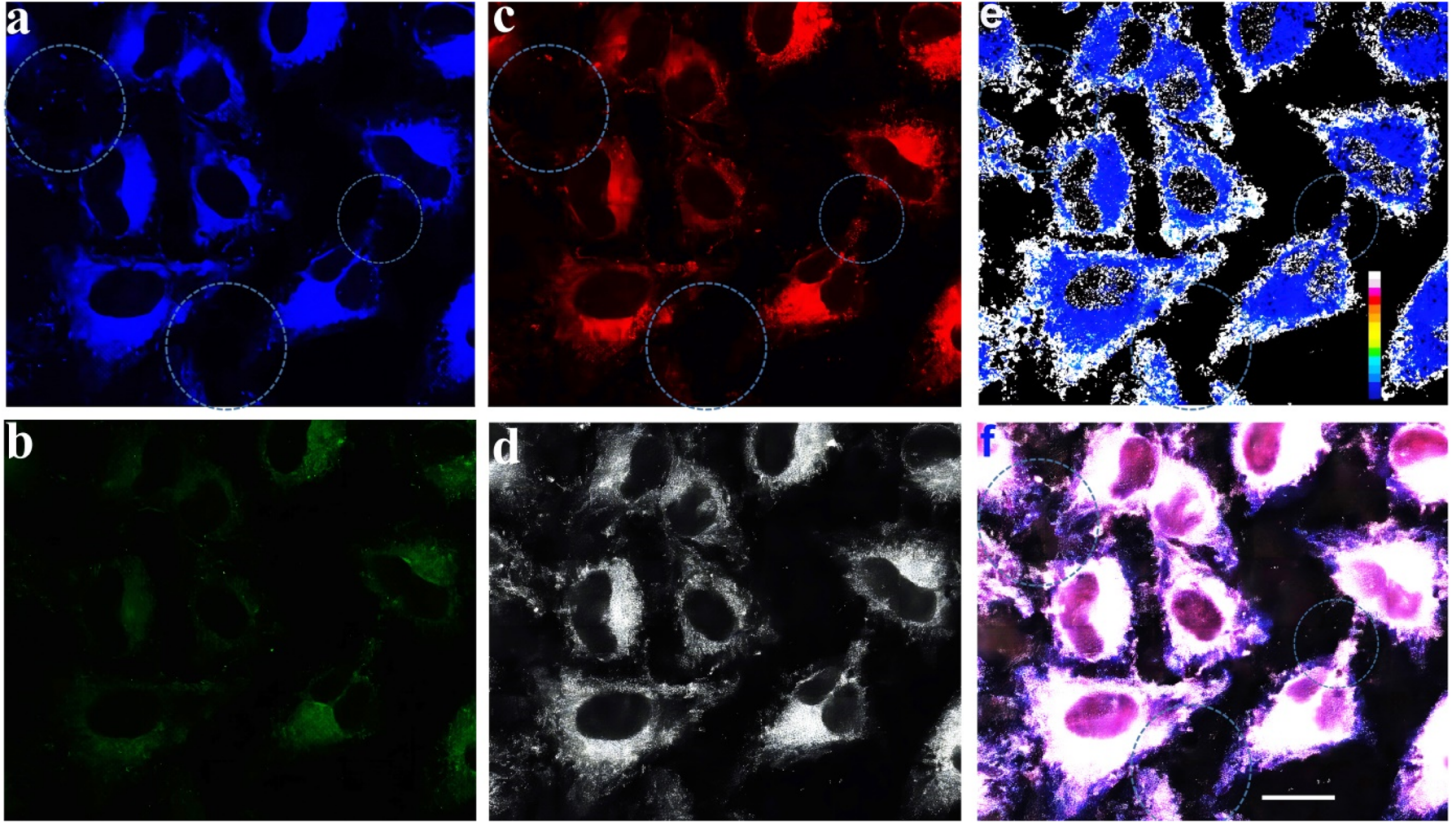
Fluorescence microscopy images of HeLa cells incubated with 4g NPs (10 μM) for 2 h at 37 °C. (a) Fluorescence microscopy image from channel 1 at 425–500 nm (excitation 405 nm); (b) fluorescence microscopy image from channel 1 at 500–700 nm (excitation 405 nm); (c) fluorescence microscopy image from channel 2 at 600–750 nm (excitation 561 nm); (d) the two-photon-excited confocal laser scanning microscopy fluorescence image from channel 3 at 400–700 nm (excitation 900 nm); (e) the emission intensity ratio of (a) and (c) of HeLa cells (image generation by ImageJ software); (f) merged image of frames (a), (b), (c) and (d); the scale bar is 25.0 μm.

In addition, Poloxamer 188 was used as a matrix to fabricate water-dispersed nanoparticles (NPs) of the dual-emission molecule 4g by a thin-film hydration method (see ESI,† Section XII). The 4g NPs exhibited dual emission at 453 nm and 639 nm with white light CIE coordinates of (0.32, 0.22) (Fig. S31†). The hydrodynamic diameter of 4g NPs was measured at about 154 nm by dynamic light scattering (DLS) (Fig. S30†). Furthermore, to explore biomedical applications, the cytotoxicity experiments in HeLa cells were first carried out with the standard methyl thiazolyl tetrazolium (MTT) assay (see ESI, Section XIII, Fig. S32†). The experimental result demonstrated that 4g NPs showed almost no toxicity in HeLa cells. Subsequently, the corresponding staining experiments were performed on HeLa cells and the fluorescence signal was observed by confocal laser scanning microscopy (Fig. 5).

Improving the imaging resolution and detection sensitivity of optical imaging and exploiting high-performance organelle imaging reagents are more favorable by dual-emission than single-emission fluorescence imaging because dual-emission materials can provide two/multi-channel information.^9,10,23^ Hence, dual-emission fluorescence imaging is of great interest in diverse scientific fields including materials science, biology, and medicine.^9,10,23^

Confocal laser scanning microscopy displayed signal distributions in the blue, green, and red regions when excited at 405 nm and 561 nm (Fig. 5a–c), and a two-photon irradiation fluorescence signal in the grey region (Fig. 5d). The emission intensity ratio map was made based on the corresponding fluorescence imaging from the blue and red emission channels using the software ImageJ (Fig. 5e). The intercellular structure can be clearly seen in Fig. 5e. Furthermore, the corresponding merged image Fig. 5f also exhibited a clearer cell contour than blue, green, and red channel images (Fig. 5f). The result indicates that the ratio map (Fig. 5e) and multi-channel merged image (Fig. 5f) contain more comprehensive information and features of cells than only reflect the cellular textures of one-channel images (Fig. 5a–d).

**Fig. 6:**
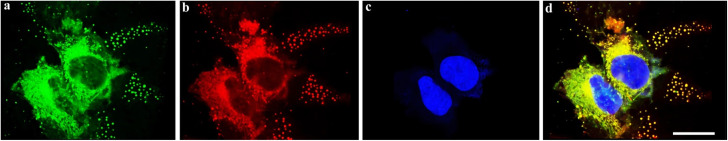
Co-staining of HeLa cells with 4g NPs and DiO to incubate for 30 min. (a) Fluorescence image of HeLa cells with the DiO-stained membrane signals (*λ*_ex_ = 488 nm, *λ*_em_ = 500–540 nm). (b) Fluorescence image of HeLa cells cultured with 4g NPs (10 μM) (*λ*_ex_ = 561 nm, *λ*_em_ = 600–750 nm). (c) The nuclei are stained with DAPI (4′,6-diamidino-2-phenylindole) and excited with a laser at 408 nm. (d) Merged image of (a), (b) and (c). (The Pearson correlation coefficient *r* = 0.90). The scale bar is 25.0 μm.

Subsequently, 4′,6-diamidino-2-phenylindole (DAPI) was employed as a reference dye for fluorescence staining in the staining experiments (see ESI, Section XIII, Fig. S33–S35† and 6c). Furthermore, co-staining experiments of HeLa cells with 4g NPs and the commercially available 3,3′-dioctadecyloxacarbocyanine perchlorate (DiO) (cell membrane-specific tracker), Mito-Tracker green (mitochondria-specific tracker), and Lyso-Tracker green (lysosome-specific tracker), respectively (Fig. S33–S35† and6) were performed. Experimental results indicated that it could specifically target the cell membrane with a Pearson's coefficient of 0.90 (Fig. 6d, see ESI, Section XIII, Fig. S33–S35†).

## Conclusions

In conclusion, the ruthenium-catalyzed C–H activation/annulation of phenothiazine-3-carbaldehydes has been developed to build a library of structurally diverse pyrido[3,4-*c*]phenothiazin-3-iums for the first time. These compounds possess distinct dual-emission characteristics with a relatively short blue emission and a relatively long red emission wavelength. Novel organic single-molecule white-light materials based on pyrido[3,4-*c*]phenothiazin-3-iums with dual-emission and TADF characteristics have been developed for the first time herein. The dual-emission molecule 4g has been fabricated as water-dispersed NPs, which were further applied in two-channel emission intensity ratio imaging and multi-channel composite imaging to raise the imaging resolution of optical imaging. Furthermore, it can specifically target the cell membrane. This work represents the first example of a ruthenium-catalyzed C–H activation/annulation to construct pyrido[3,4-*c*]phenothiazin-3-iums with dual-emission and TADF characteristics, which opens up a new avenue for rapid screening of high-performance organic single-molecule white-light-emitting and two/multi-channel composite imaging materials.

## Data availability

All experimental data associated with this work are provided in the ESI.†

## Author contributions

J. L., K. W., L. W., and X. Y. performed the experiments and analyzed the data. B. L. designed and directed the project and wrote the manuscript. All authors contributed to discussions.

## Conflicts of interest

There are no conflicts to declare.

## Supplementary Material

SC-OLF-D4SC07825J-s001

SC-OLF-D4SC07825J-s002
