# Supplementary material for: Ruthenium-catalyzed C–H bond activation and annulation of phenothiazine-3-carbaldehydes: facile access to dual-emission materials
Source: Chem Sci. 2025 Jan 16;16(7):3107–13. doi: 10.1039/d4sc07825j (PMC11740230; doi:10.1039/d4sc07825j)
Supplement: SC-016-D4SC07825J-s001 [file SC-016-D4SC07825J-s001.pdf]

Supplementary Information (SI) for Chemical Science.
This journal is © The Royal Society of Chemistry 2025

# Supporting Information

## Ruthenium-Catalyzed C−H Bond Activation and Annulation of Phenothiazine-3-Carbaldehydes: Facile Access to Dual-Emission Materials

Junxiang Liu, Kangmin Wang, Liqiu Wan, Xianhui Yang, and Bijin Li*

Chongqing Key Laboratory of Natural Product Synthesis and Drug Research, School of Pharmaceutical Sciences, Chongqing University, Chongqing 401331, P. R. China.

# Table of Contents

I. General remarks ........ S3

II. Synthesis of substrates **1a** and **1b** ........ S4

III. Optimization of reaction conditions ........ S5

IV. General synthesis procedure for compounds **4a-4i** ........ S8

V. Absorption, fluorescence spectra of **4a-4i** ........ S9

VI. The excited state lifetimes of **4g** in film ........ S21

VII. AIE assay ........ S23

VIII. ROS generation assays ........ S24

IX. Fluorescence emission spectra of **4g** in different atmospheres ........ S25

X. Cyclic voltammogram experiment ........ S26

XI. DFT Calculation of **4g** ........ S26

XII. Nanoparticle preparation and characterization ........ S37

XIII. Cell imaging experiments and MTT assays ........ S38

XIV. Experimental data for the substrates **1a** and **1b** ........ S41

XV. Experimental data for the described substances ........ S41

XVI. Crystal ........ S50

XVII. References ........ S51

XVIII. Copies of $^{1}H$ and $^{13}C$ NMR spectra ........ S54

# I. General remarks

NMR spectra were obtained on Agilent 400 MR DD 2 (400 MHz) spectrometer. $^{1}H$ NMR (400 MHz) and $^{13}C$ NMR (100 MHz) chemical shifts were measured relative to $SiMe_4$, $CDCl_3$ using the chemical shift of residual solvent peaks as reference ($SiMe_4$: δ 0 ppm for $^{1}H$ NMR and $^{13}C$ NMR; $CDCl_3$: 7.26 ppm for $^{1}H$ NMR and 77.16 ppm for $^{13}C$ NMR). High-resolution mass spectra (HR-MS) were recorded using Agilent 6546 Q-TOF LC/MS system with Electrospray Ionization (ESI). X-Ray single-crystal diffraction data were collected on an Agilent Super Nova Microfocal spot single-crystal diffractometer. The $CIE_{1931}$ chromaticity coordinates were calculated using a Color Coordinate.exe program. UV−vis spectra were recorded on Agilent Cary 60 spectrometer. Fluorescence spectra were collected on the F-7000 fluorescence spectrometer. The fluorescence decay curve and quantum yield were collected on the Edinburgh FLS1000 Steady transient fluorescence spectrometer. Absorption spectra were obtained from Agilent Cary60. The multi-angle particle size and Zeta potential data were collected on the Brucker Omni. The confocal imaging experiments were performed on Leica TCS SP8 and Leica TCS SP8 DIVE. Cyclic voltammetry (CV) measurement was carried out on DH7001 using an $Ag/Ag^{+}$ reference electrode, a platinum wire counter electrode, and a platinum plate working electrode. Energy levels were calculated with respect to a standard ferrocene/ferrocenium ($Fc/Fc^{+}$) redox couple.

Unless otherwise noted, all reagents were obtained from commercial sources and used directly. Thin layer chromatography (TLC) was performed on EMD precoated plates (silica gel 60 F254, Art 5715) and visualized by fluorescence quenching under UV light. Column chromatography was performed through silica gel (200-300 mesh) using a proper solvent system. All syntheses and manipulations were carried out under an air atmosphere using standard Schlenk. Diphenylacetylene, 2-methoxy-10-methyl-10*H*-phenothiazine and its derivatives was prepared according to the literature procedures.[1] All calculations were performed using the Gaussian 16, Revision A.03.[2] The time-dependent density functional theory (TDDFT) calculations of the excitation energies were calculated at the optimized geometries of the ground states. The compounds used for optical experiments were purified by recrystallization three times to ensure purity. The solvents used for the fluorescence test were freshly prepared after distillation and were optically pure solvents.

## II. Synthesis of substrates 1a and 1b

**General procedure for the synthesis of substrates 1a and 1b:**

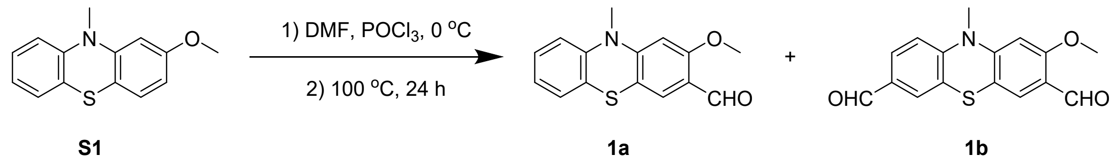


**Scheme S1**. Synthesis of substrates **1a** and **1b**.

Dissolve 2-methoxy-10-methyl-10*H*-phenothiazine (2.43 g, 10 mmol) in 15 ml of dimethylformimide. Cool down the reaction mixture to 0 °C. Add phosphorus

oxychloride (15.3 g, 100 mmol) dropwise to the reaction mixture at 0-5 °C. Increase the temperature of the reaction mixture to 100 °C. Stir the reaction mixture for 24 hours at 100 °C. Then, cool down the reaction mixture to room temperature. Pour the reaction mixture into a cold aqueous potassium hydroxide solution. Extract the reaction mixture with dichloromethane and distilled water, and then separate the organic layer. Dry the organic layer with anhydrous sodium sulfate. Purify the crude product by column chromatography using petroleum ether/ethyl acetate (2/1, v/v) as the eluent. Recrystallize the product from the eluent mixture of solvent.

## III. Optimization of reaction conditions and plausible catalytic cycle

A shrink tube with a magnetic stir bar was charged with 2-methoxy-10-methyl-10*H*-phenothiazine-3-carbaldehyde (**1a**, 0.1 mmol), aniline (**2a**, 0.1-0.15 mmol), 1,2-diphenyl ethyne (**3a**, 0.1-0.15 mmol), catalyst (5 mol%), oxidant (0.2 mmol), additive (0.1 mmol) and solvent (1 ml), and then heated at indicated temperature for indicated time. The mixture was cooled to room temperature, and diluted with 5 mL of DCM. The mixture was filtered through a celite pad and washed with 20 mL of DCM. The organic phase was evaporated under a vacuum. The crude product was purified by column chromatography on silica gel to provide the desired products.

**Table S1.** Optimization of reaction conditions[a]

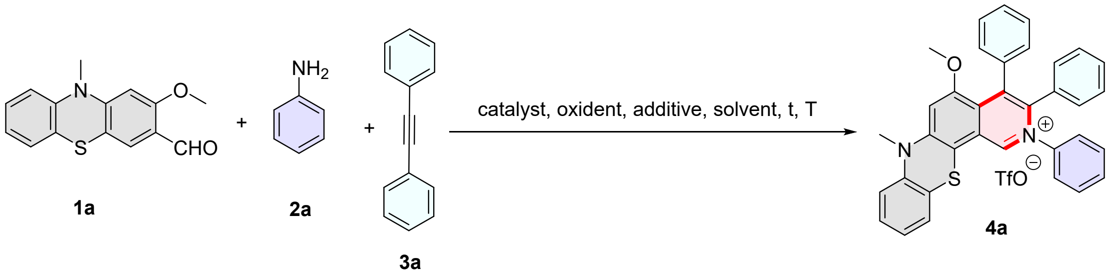


| Entry | Ratio | Metal complex | Oxidant | Additive | Solvent | T (°C) | Time (h) | Yield[b] (%) |
|---|---|---|---|---|---|---|---|---|
| 1 | (**1a**:**2a**:**3a** = 1 : 1.5 : 1.5) | $[RuCl_2(p\text{-cymene})]_2$ | $Cu(OAc)_2$ | AcOH | DCE | 100 | 12 | 14% |
| 2 | (**1a**:**2a**:**3a** = 1 : 1.5 : 1.5) | $[RuCl_2(p\text{-cymene})]_2$ | $Cu(OAc)_2$ | TfOH | DCE | 100 | 12 | 72% |
| 3 | (**1a**:**2a**:**3a** = 1 : 1.5 : 1.5) | $[RuCl_2(p\text{-cymene})]_2$ | $Cu(OAc)_2$ | TfOH | DCE | 120 | 12 | 87% |
| 4 | (**1a**:**2a**:**3a** = 1 : 1.5 : 1.5) | $[RuCl_2(p\text{-cymene})]_2$ | $Cu(OAc)_2$ | TfOH | DCE | 140 | 8 | 69% |
| 5[c] | (**1a**:**2a**:**3a** = 1 : 1.5 : 1.5) | $[RuCl_2(p\text{-cymene})]_2$ | $Cu(OAc)_2$ | TfOH | DCE | 140 | 12 | 92% |
| 6 | (**1a**:**2a**:**3a** = 1 : 1.5 : 1.5) | $[RuCl_2(p\text{-cymene})]_2$ | $Cu(OAc)_2$ | TfOH | DCE | 140 | 16 | 90% |
| 7 | (**1a**:**2a**:**3a** = 1 : 1.5 : 1.5) | $[RuCl_2(p\text{-cymene})]_2$ | $Cu(OAc)_2$ | AcOH | DCE | 140 | 12 | 18% |
| 8 | (**1a**:**2a**:**3a** = 1 : 1.5 : | $[RuCl_2(p\text{-cymene})]_2$ | $O_2$ | TfOH | DCE | 140 | 12 | 52% |

| | | | | | | | | |
|---|---|---|---|---|---|---|---|---|
| | 1.5) | | | | | | | |
| 9 | (**1a:2a:3a** = 1 : 1.5 : 1.5) | $[RuCl_2(p\text{-}cymene)]_2$ | $O_2$ | TfOH | DCE | 140 | 24 | 50% |
| 10 | (**1a:2a:3a** = 1 : 1.5 : 1.5) | $[RuCl_2(p\text{-}cymene)]_2$ | $O_2$ | TfOH | DCE | 140 | 48 | 45% |
| 11[d] | (**1a:2a:3a** = 1 : 1.5 : 1.5) | $[RuCl_2(p\text{-}cymene)]_2$ | $O_2$ | TfOH | DCE | 140 | 24 | trace |
| 12 | (**1a:2a:3a** = 1 : 1.5 : 1.5) | $[RuCl_2(p\text{-}cymene)]_2$ | AgTFA | TfOH | DCE | 140 | 12 | trace |
| 13 | (**1a:2a:3a** = 1 : 1.5 : 1.5) | $[RuCl_2(p\text{-}cymene)]_2$ | AgOAc | TfOH | DCE | 140 | 12 | trace |
| 14 | (**1a:2a:3a** = 1 : 1 : 1) | $[RuCl_2(p\text{-}cymene)]_2$ | $Cu(OAc)_2$ | TfOH | DCE | 140 | 12 | 80% |
| 15 | (**1a:2a:3a** = 1 : 1.5 : 1) | $[RuCl_2(p\text{-}cymene)]_2$ | $Cu(OAc)_2$ | TfOH | DCE | 140 | 12 | 85% |
| 16 | (**1a:2a:3a** = 1 : 1 : 1.5) | $[RuCl_2(p\text{-}cymene)]_2$ | $Cu(OAc)_2$ | TfOH | DCE | 140 | 12 | 75% |
| 17 | (**1a:2a:3a** = 1 : 1.5 : 1.5) | - | $Cu(OAc)_2$ | TfOH | DCE | 140 | 12 | N.D. |
| 18 | (**1a:2a:3a** = 1 : 1.5 : 1.5) | $[RuCl_2(p\text{-}cymene)]_2$ | - | TfOH | DCE | 140 | 12 | trace |
| 19 | (**1a:2a:3a** = | $[RuCl_2(p\text{-}cymene)]_2$ | $Cu(OAc)_2$ | - | DCE | 140 | 12 | trace |

| | | | | | | | | |
|---|---|---|---|---|---|---|---|---|
| | 1 : 1.5 : 1.5) | | | | | | | |
| 20 | (**1a**:**2a**:**3a** = 1 : 1.5 : 1.5) | [RuCl$_2$(p-cymene)]$_2$ | Cu(OAc)$_2$ | TfOH | DMF | 140 | 12 | trace |
| 21 | (**1a**:**2a**:**3a** = 1 : 1.5 : 1.5) | [RuCl$_2$(p-cymene)]$_2$ | Cu(OAc)$_2$ | TfOH | DMSO | 140 | 12 | N.D. |
| 22 | (**1a**:**2a**:**3a** = 1 : 1.5 : 1.5) | [RuCl$_2$(p-cymene)]$_2$ | Cu(OAc)$_2$ | TfOH | NMP | 140 | 12 | trace |
| 23 | (**1a**:**2a**:**3a** = 1 : 1.5 : 1.5) | [RhCp*Cl$_2$]$_2$ | Cu(OAc)$_2$ | TfOH | DCE | 140 | 12 | 80% |

[a] **1a** (0.1 mmol, 1.0 equiv), **2a** (0.1-0.15 mmol), **3a** (0.1-0.15 mmol), oxident (0.2 mmol, 2.0 equiv), solvent (1 ml), 100-140 °C, 8-16 h. [b] Yield is based on **1a**, determined by $^1$H-NMR using dibromomethane as the internal standard. [c] Isolated yields. [d] [RuCl$_2$(p-cymene)]$_2$ (2.5 mol%). N.D.: No detected. DCE: 1, 2-dichloroethane, DMF: *N*,*N*-dimethylformamide. DMSO: Dimethyl sulfoxide. NMP: *N*-Methyl-2-pyrrolidone.

Furthermore, a plausible catalytic cycle is proposed (Fig. S1). Initially, the five-membered ruthenacycle intermediate **I** is generated via imine (in situ generation from **1a** and **2a**) nitrogen coordinated with ruthenium (II) species and *ortho*-C–H bond activation. Next, the alkyne **3a** coordinates with the intermediate **I** to form the intermediate **II**, and subsequently inserts into the Ru-C bond to give the seven-membered ruthenacycle **III**. Finally, intermediate **III** undergoes reductive elimination to produce the cyclization product **4** and release Ru$^{I}$. The Ru$^{II}$ species is regenerated through Cu(OAc)$_2$ oxidation.

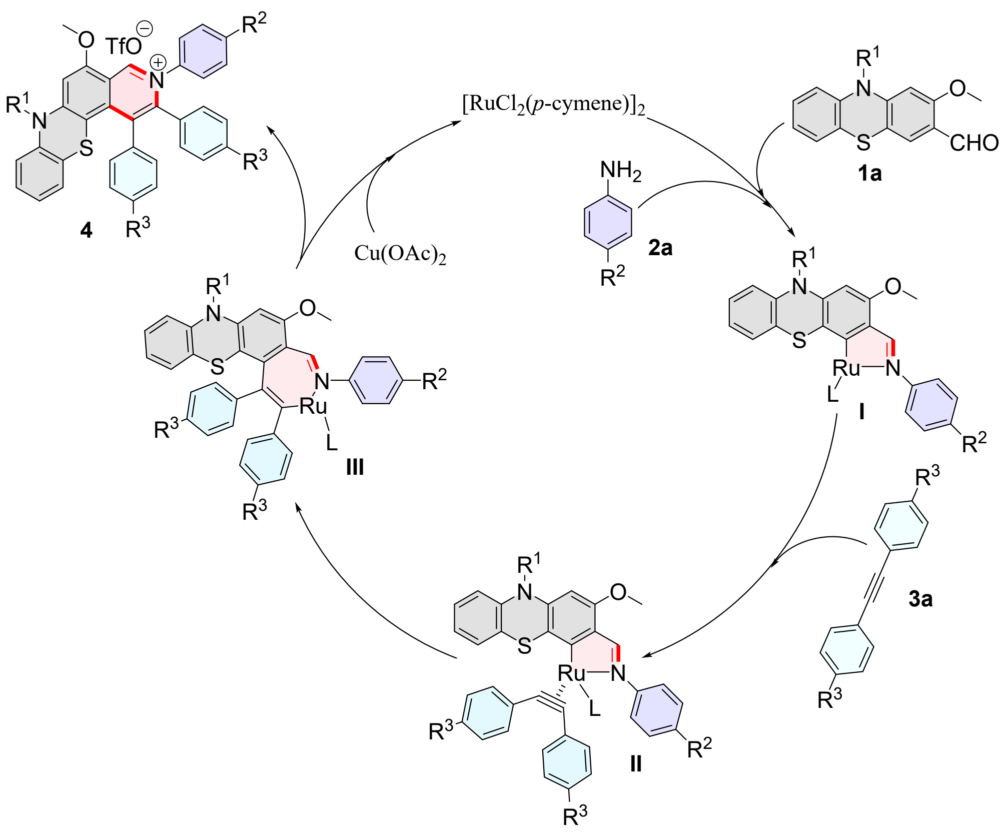


**Fig. S1** Tentative mechanism for ruthenium-catalyzed C–H activation/annulation.

## IV. General synthesis procedure for compounds 4a-4i

A 10 mL reaction tube with a magnetic stir bar was charged with **1a** (0.1 mmol), **2a** (0.15 mmol, 1.5 equiv), **3a** (0.15 mmol, 1.5 equiv), $[RuCl_2(p\text{-cymene})]_2$ (3.1 mg, 5 mol%), $Cu(OAc)_2$ (36.2 mg, 0.2 mmol), TfOH (8.8 μL, 0.1 mmol) and DCE (1 mL) under air atmosphere. The resulting mixture was stirred at 140 ºC for 12 h. The reaction mixture was cooled to ambient temperature, and diluted with 5 mL of DCM. The mixture was filtered through a celite pad and washed with 20 mL of DCM. The organic phase was evaporated under a vacuum. The residue was purified by column chromatography (DCM/MeOH = 20/1, v/v) on silica gel to provide the desired products **4a-4i**.

## V. Absorption, fluorescence spectra of 4a-4i

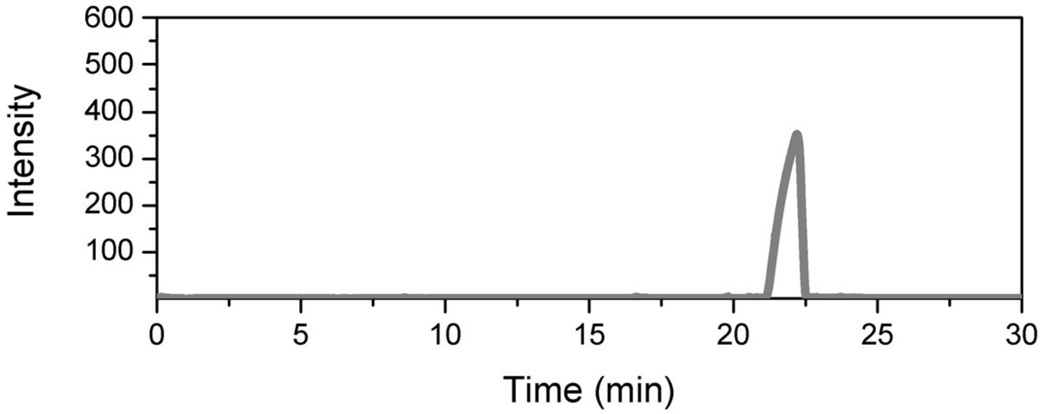


**Fig. S2** The high-performance liquid chromatography (HPLC) spectrogram of **4g** (HPLC condition: $CH_3CN$:$H_2O$=50:50, 5 min, $CH_3CN$:$H_2O$ = 70:30, 10 min, $CH_3CN$:$H_2O$ = 90:10, 15 min, flow rate = 1.0 mL/min, wave length = 510 nm).

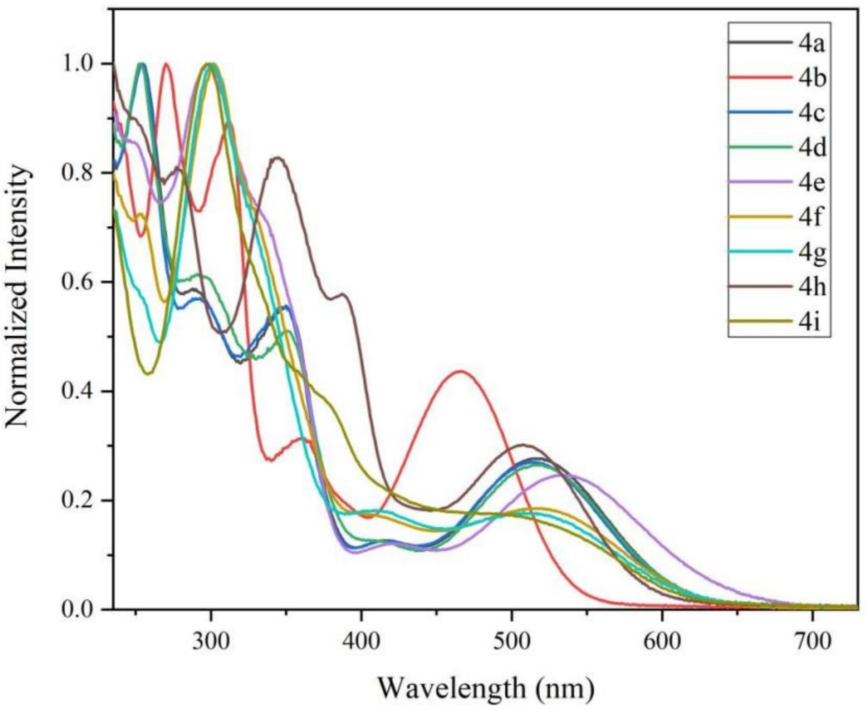


**Fig. S3** Normalized UV-Vis absorption spectra of **4a-4i** in $CH_2Cl_2$ ($5.0\times10^{-5}$ M).

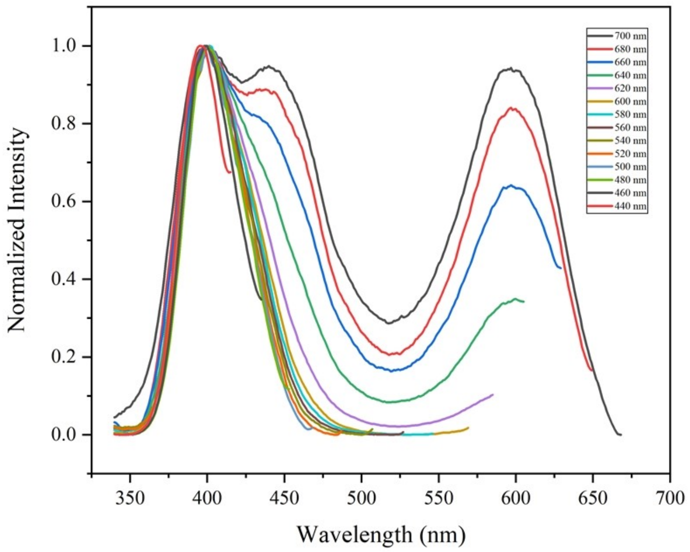


**Fig. S4** The emission-wavelength-dependent excitation spectra of **4a** (600 μM) in $CH_2Cl_2$.

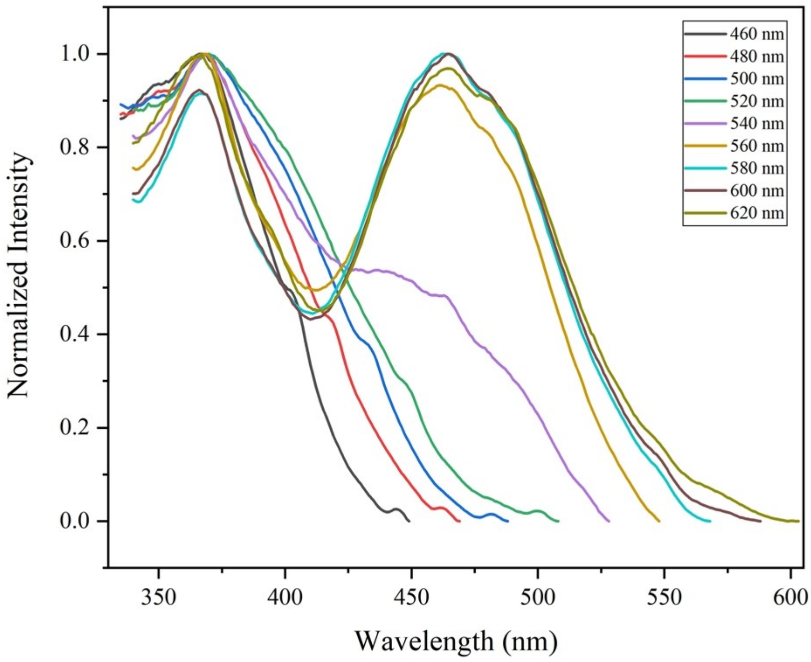


**Fig. S5** The emission-wavelength-dependent excitation spectra of **4b** (0.1 μM) in $CH_2Cl_2$.

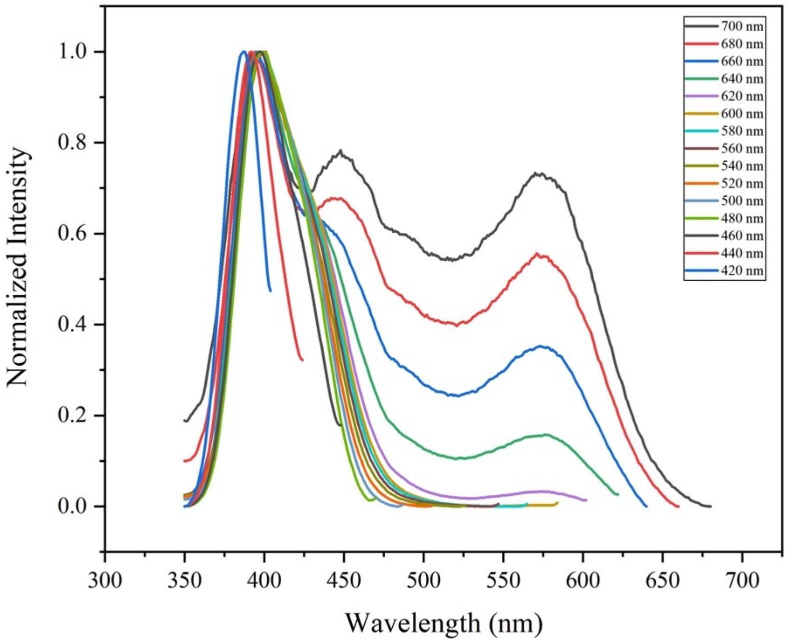


**Fig. S6** The emission-wavelength-dependent excitation spectra of **4c** (600 μM) in $CH_2Cl_2$.

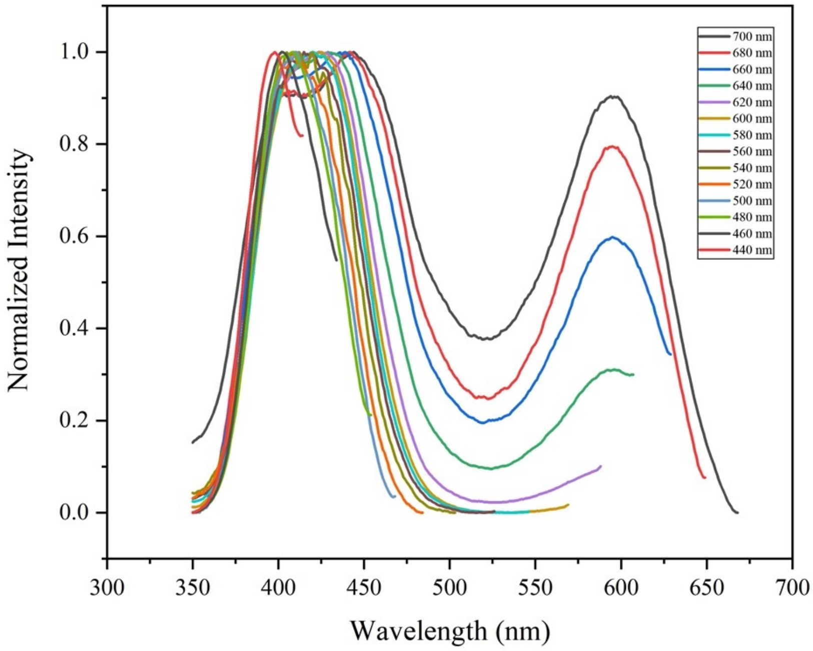


**Fig. S7** The emission-wavelength-dependent excitation spectra of **4d** (600 μM) in $CH_2Cl_2$.

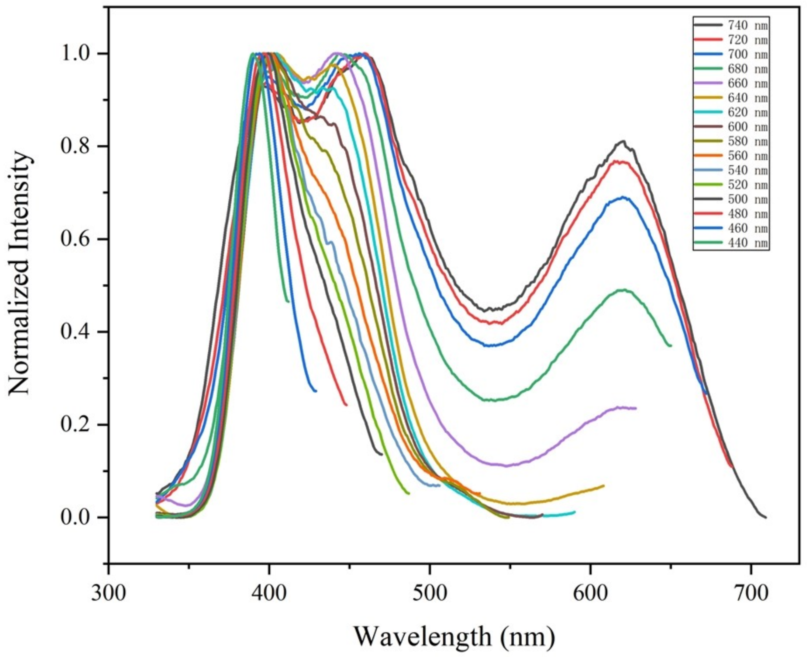


**Fig. S8** The emission-wavelength-dependent excitation spectra of **4e** (800 μM) in $CH_2Cl_2$.

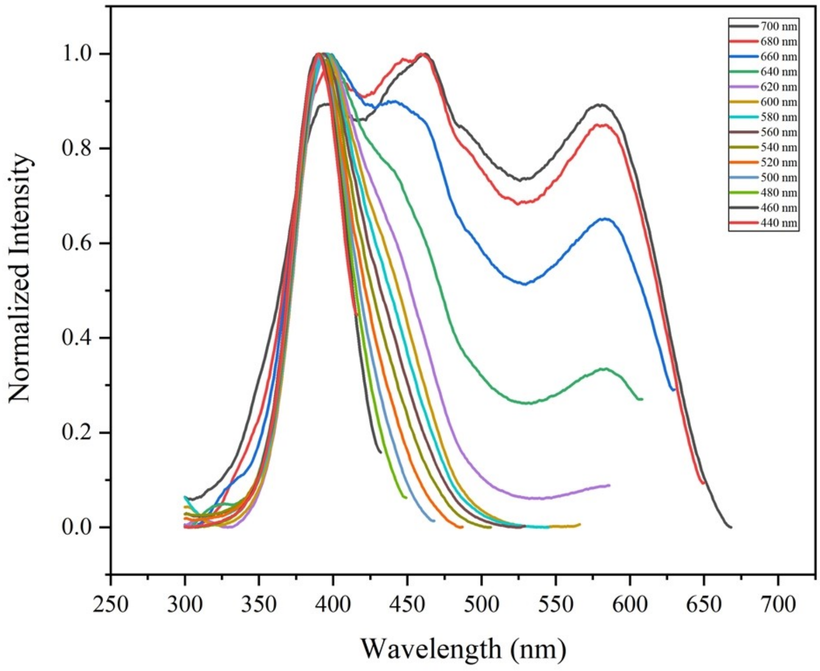

**Fig. S9** The emission-wavelength-dependent excitation spectra of **4f** (250 μM) in $CH_2Cl_2$.

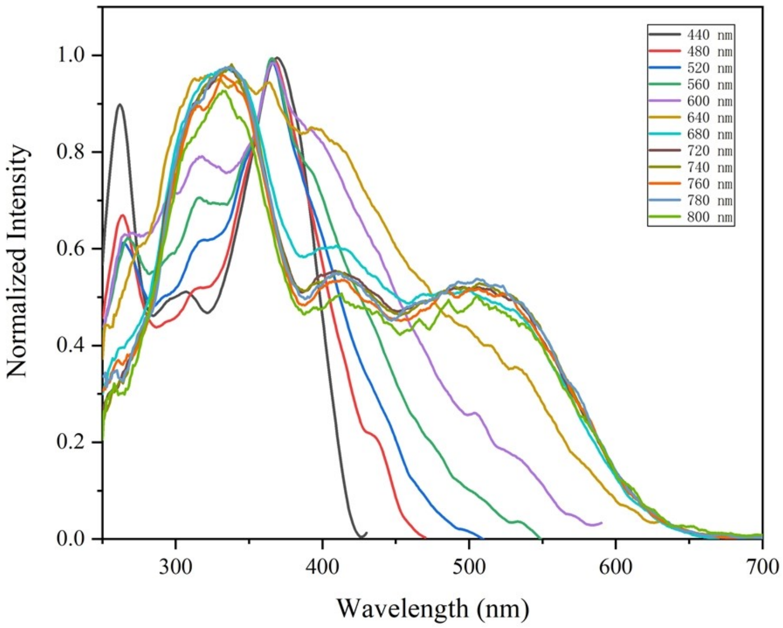


**Fig. S10** The emission-wavelength-dependent excitation spectra of **4g** (500 μM) in $CH_2Cl_2$.

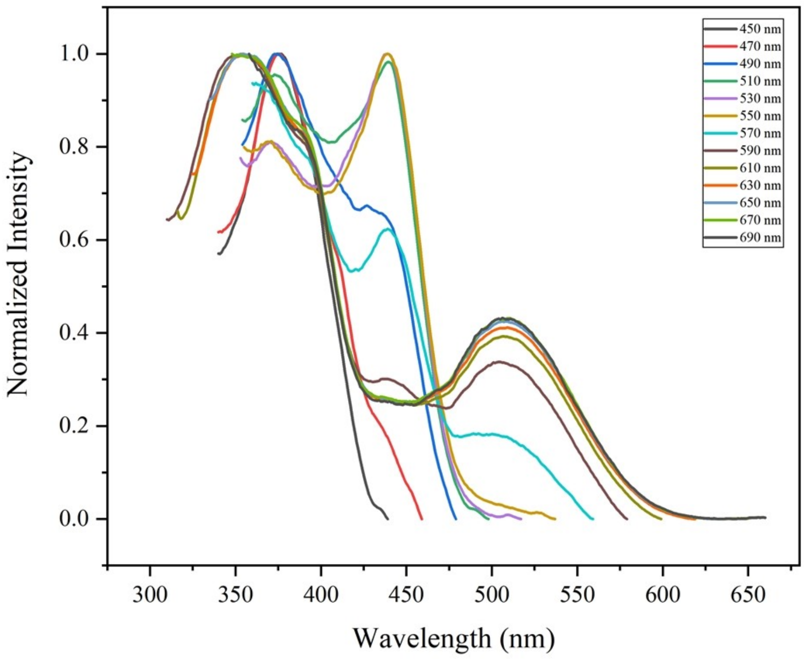


**Fig. S11** The emission-wavelength-dependent excitation spectra of **4h** (2.5 μM) in $CH_2Cl_2$.

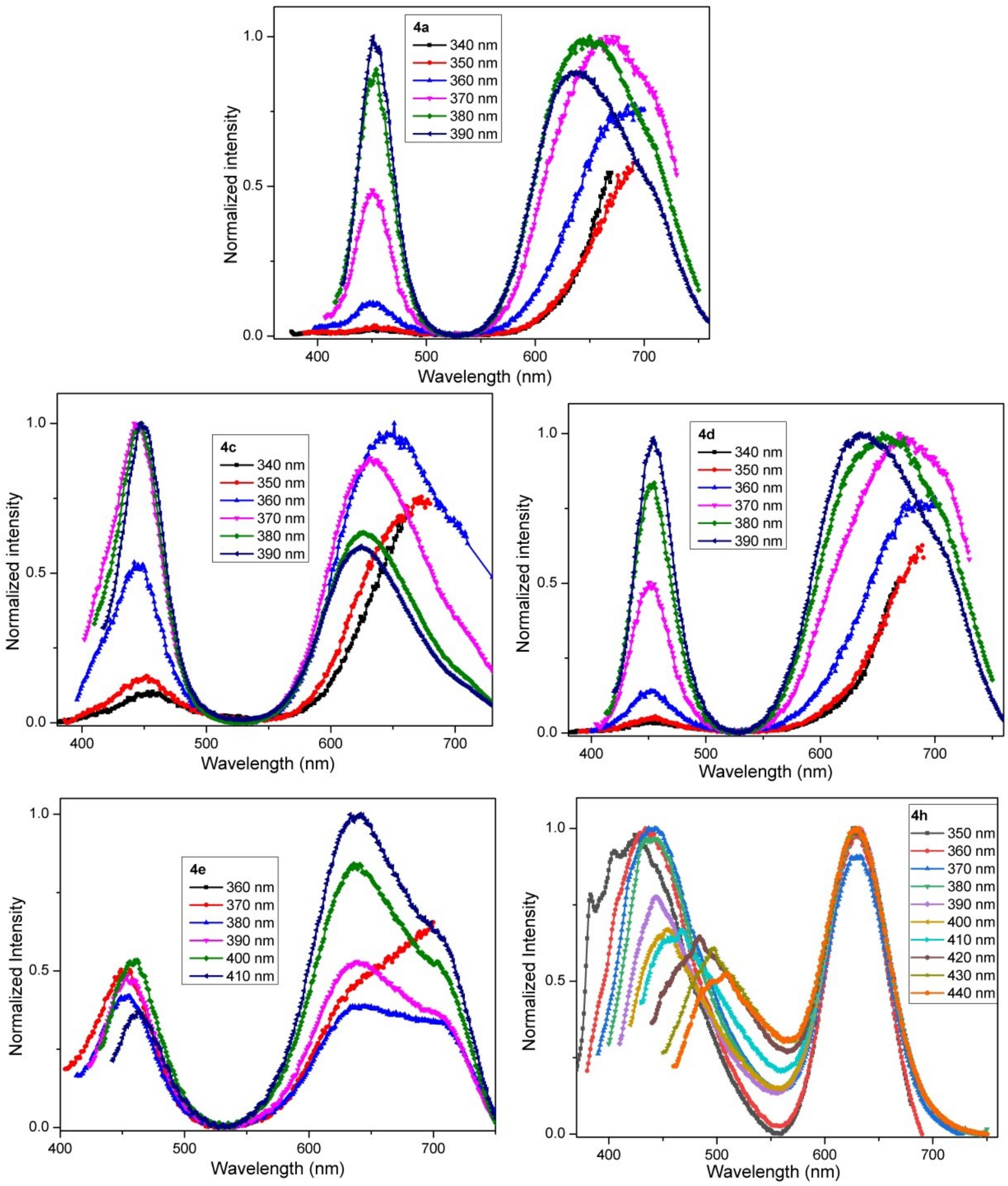


**Fig. S12** Excitation-wavelength-dependent fluorescence spectra of **4a-4h** in $CH_2Cl_2$ (Concentration, **4a**, **4c**, **4d,** 600 μM; **4e**, 800 μM; **4h**, 2.5 μM).

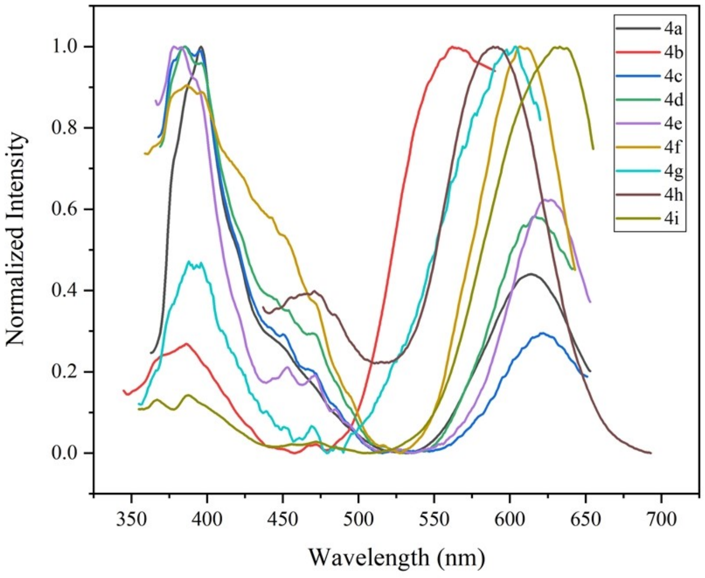


**Fig. S13** Normalized fluorescence emission spectra of **4a-4i** in PMMA films (**4a**, **4c**, **4d**, **4e**, **4f**, **4h**: 0.2 wt %; **4b**, **4i**: 0.1 wt %, **4g**: 0.15 wt %).

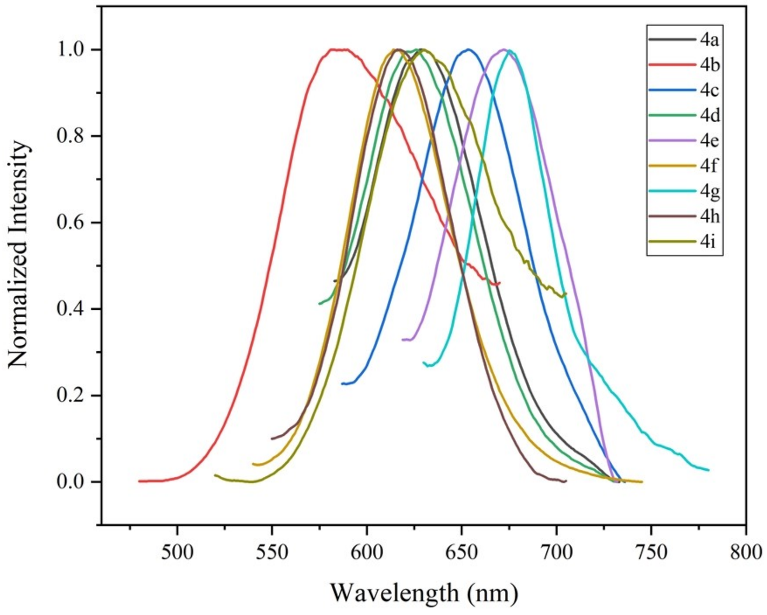


**Fig. S14** Normalized fluorescence emission spectra of solid powders **4a-4i**.

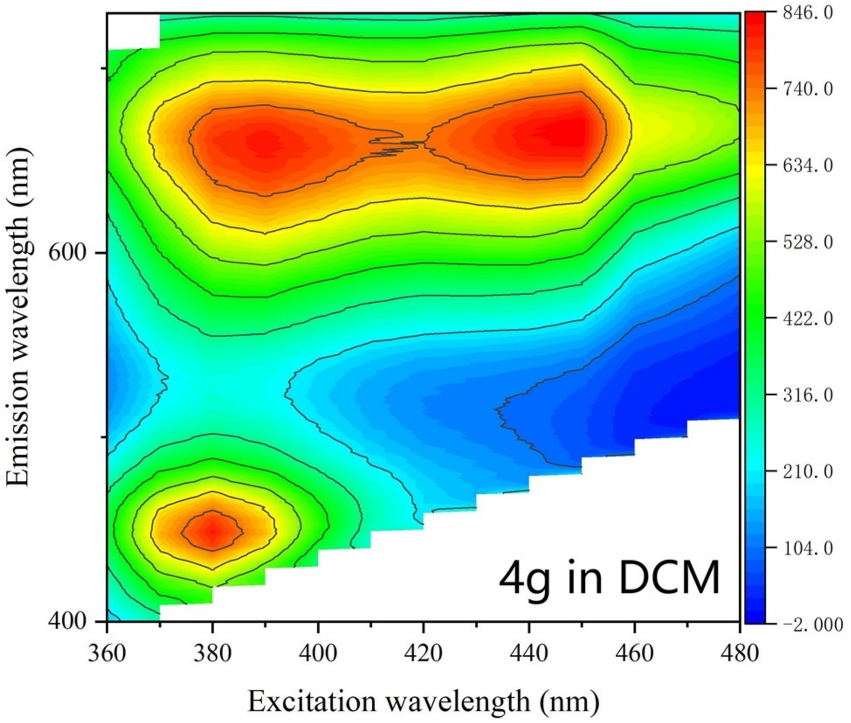


**Fig. S15** Excitation-emission maps of **4g** in DCM (500 μM).

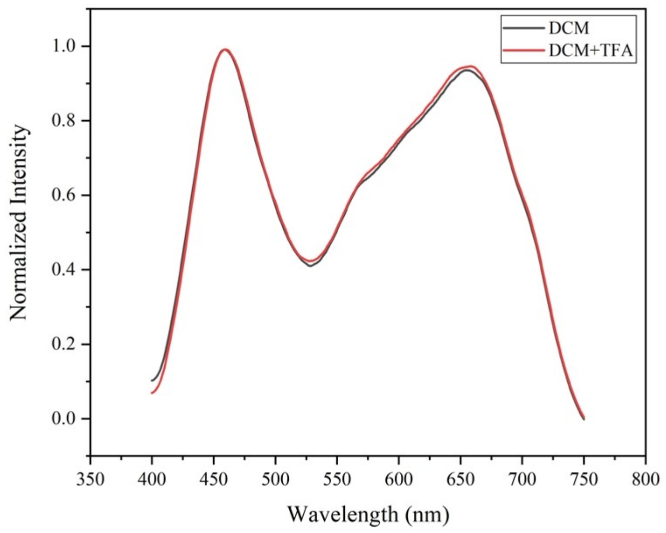


**Fig. S16** The fluorescence spectra of **4g** in $CH_2Cl_2$ acidified with TFA (Concentration, **4g**, 130 μM; TFA, 0.4 uL/mL).

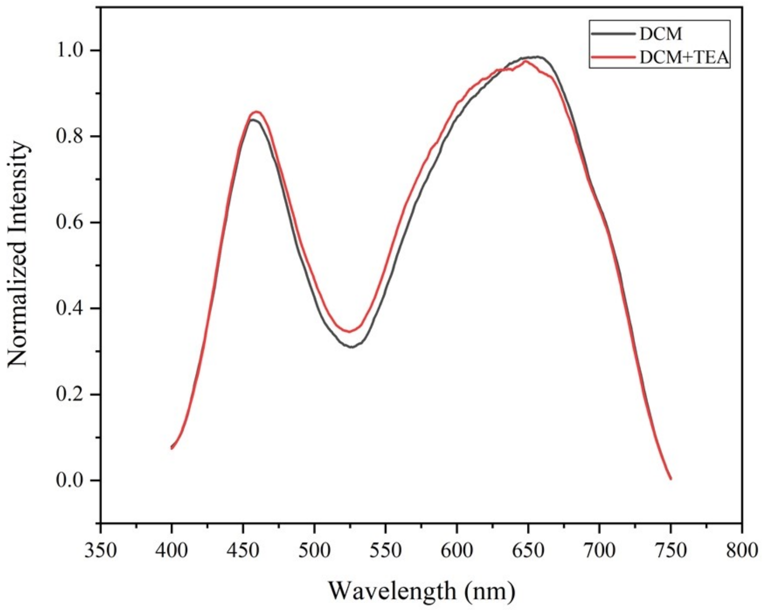


**Fig. S17** The fluorescence spectra of **4g** in $CH_2Cl_2$ based with triethylamine (Concentration, **4g**, 850 μM; TEA, 0.4 uL/mL).

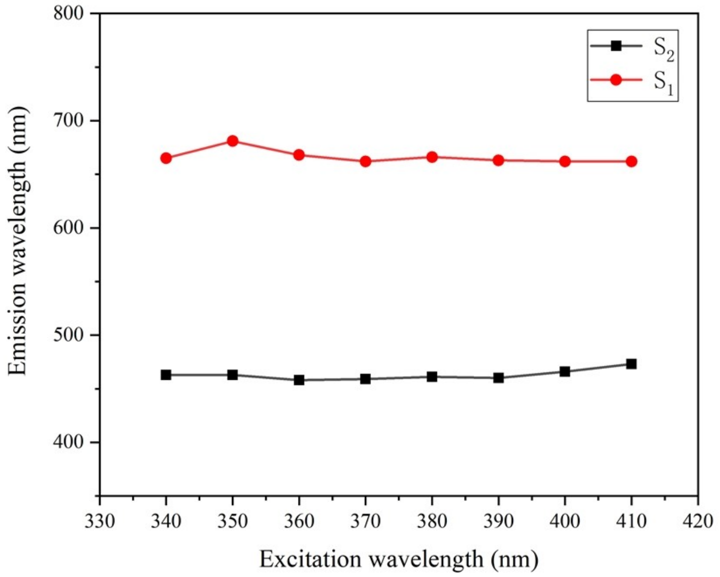


**Fig. S18** Emission wavelength as functions of excitation wavelength of **4g** (500 μM) in $CH_2Cl_2$.

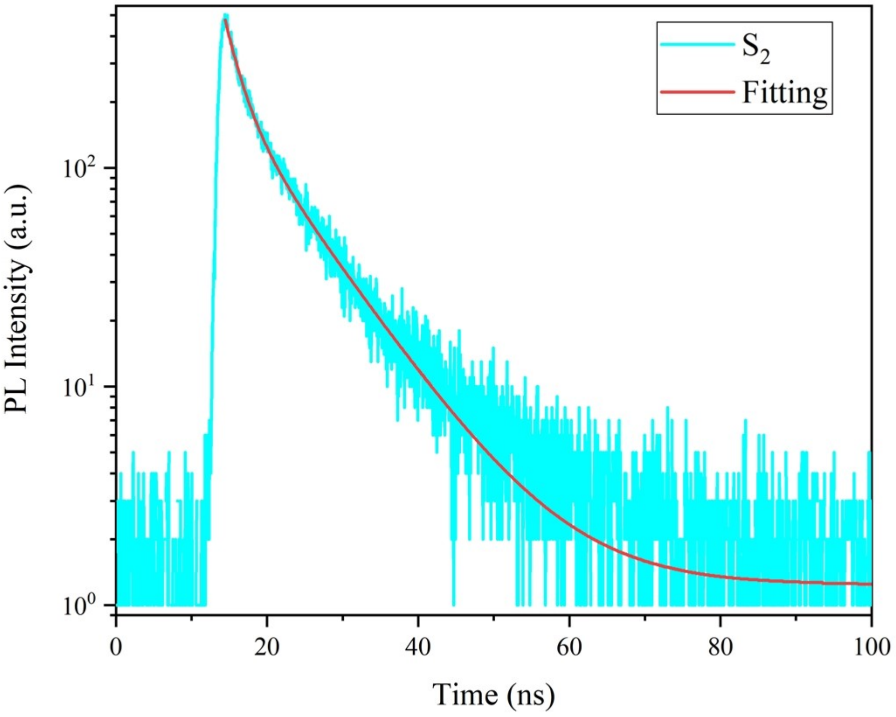


**Fig. S19** Fluorescence decay trace and calculated lifetime (t = 4.81 ns) of a dilute solution of **4g** in $CH_2Cl_2$ (50 μM)

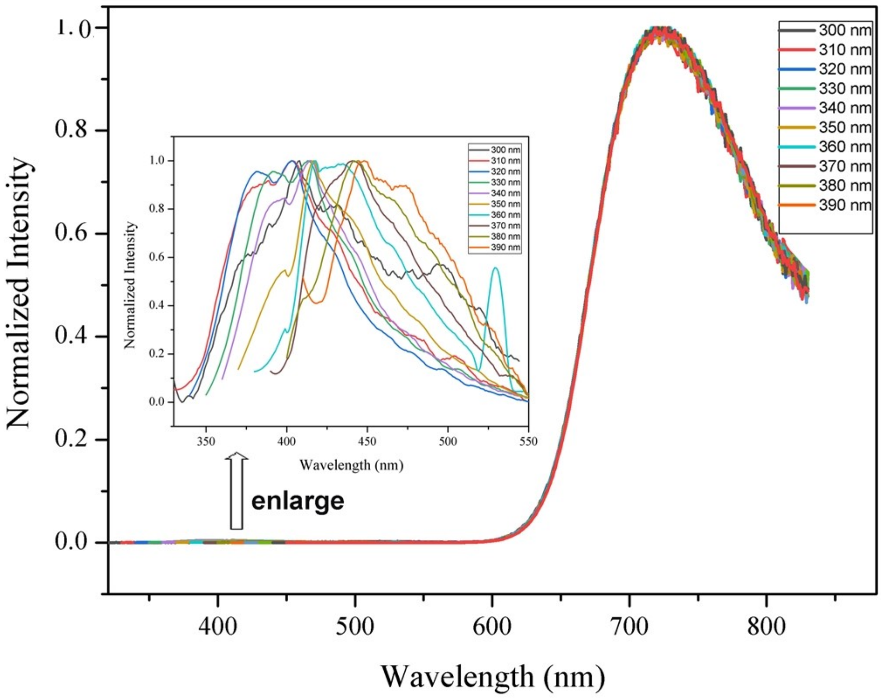

**Fig. S20** Excitation-wavelength-dependent fluorescence spectra of **4g** (crystal). Partial enlarged image (wavelength range 330-550 nm).

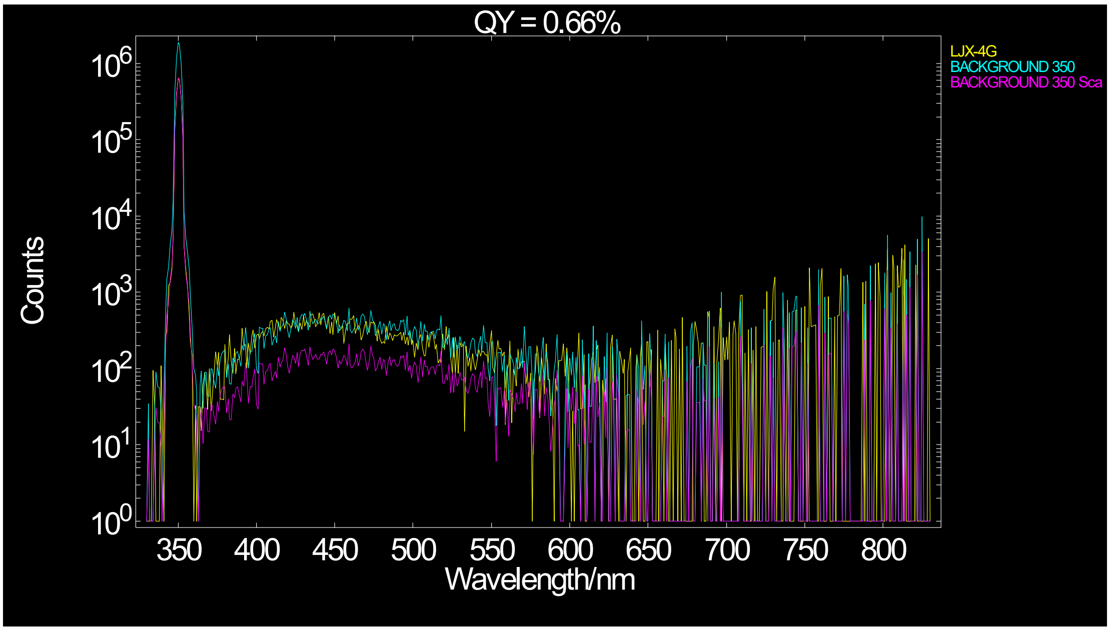


**Fig. S21** The quantum yield of the $S_1$ (0.66%) emissions for the dye **4g** in DCM (concentration: 50 μM). The PLQY were determined on an Edinburgh FLS1000 steady transient fluorescence spectrometer.

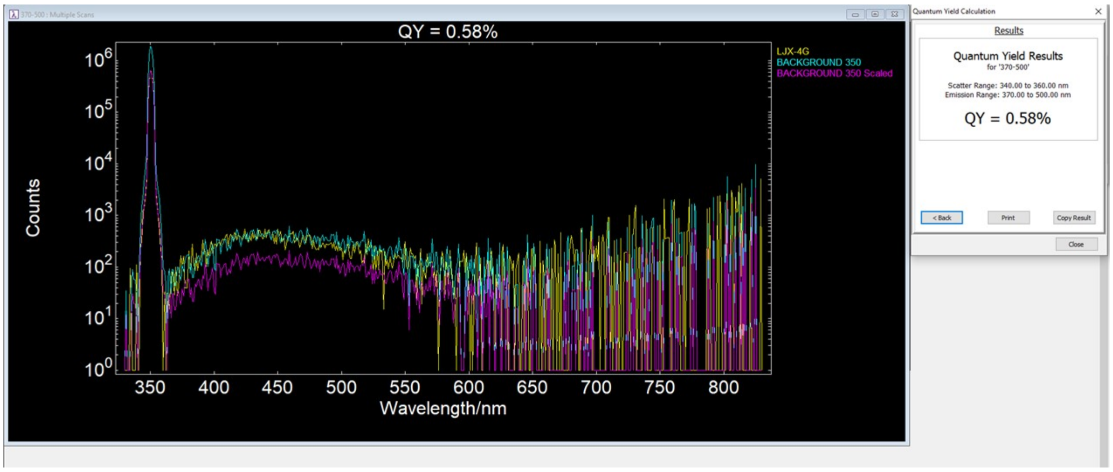


**Fig. S22** The quantum yield of the $S_2$ (0.58%) emissions for the dye **4g** in DCM (concentration: 50 μM).

## VI. The excited state lifetimes of 4g in film

$CBZ_2$-$F_1$ and TBCPF were selected as the host material.

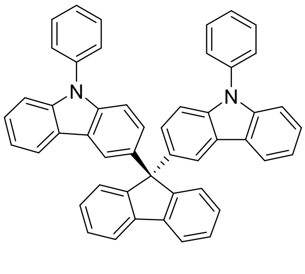

$CBZ_2$-$F_1$

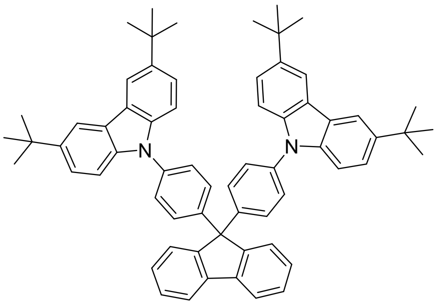

TBCPF

$CBZ_2$-$F_1$ = 3,3'-(9*H*-fluorene-9,9-diyl)bis(9-phenyl-9*H*-carbazole) (HOMO: -5.5 eV, LUMO: -2.1 eV)

TBCPF = 9,9-di(4,4'-bis(3,6-di-tert-butylcarbazole)-phenyl)-9*H*-fluorene (HOMO: -5.5 eV, LUMO: -2.1 eV)

**Table S2**. The excited state lifetimes of **4g**.[a]

| Compound | $\lambda_{em}$ [nm] | Lifetime |
|---|---|---|
| **4g** in film (3 wt %) | 700 | 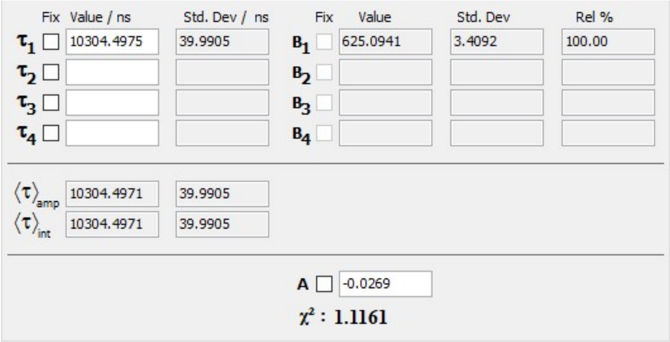 |

| | | |
|---|---|---|
| **4g** in CBZ$_2$-F$_1$ film (3 wt %) | 700 | 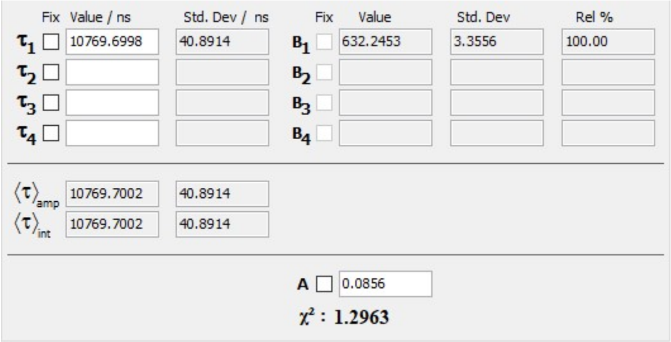 |
| **4g** in CBZ$_2$-F$_1$ film (5 wt %) | 700 | 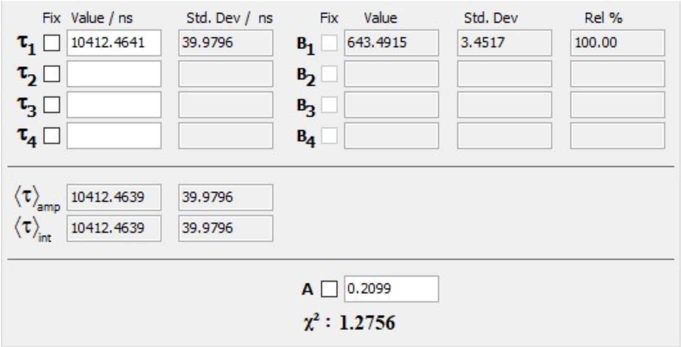 |

[a] The excited state lifetimes were determined on an Edinburgh FLS1000 steady transient fluorescence spectrometer.

**(a) 4g in TBCPF film (3 wt %)**

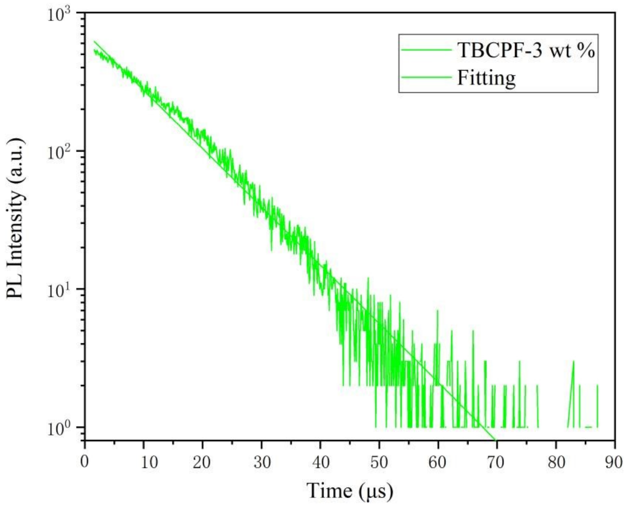


**Fig. S23** Fluorescence lifetime decay curve of **4g** in TBCPF film (3 wt %).

**(b) 4g in CBZ$_2$-F$_1$ film (3 wt %)**

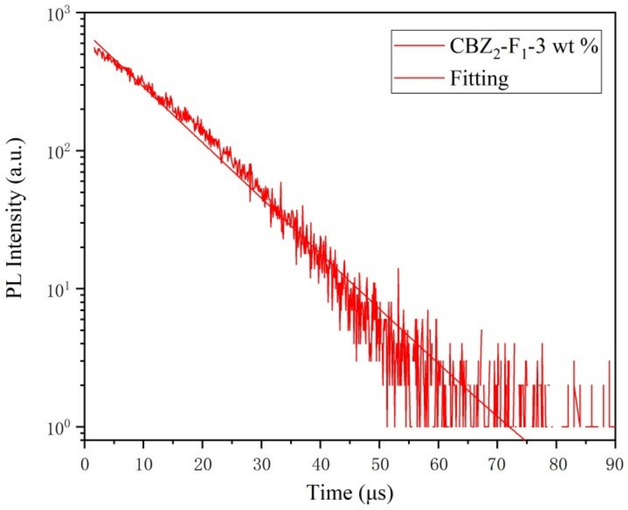


**Fig. S24** Fluorescence lifetime decay curve of **4g** in $CBZ_2$-$F_1$ film (3 wt %).

**(c) 4g in $CBZ_2$-$F_1$ film (5 wt %)**

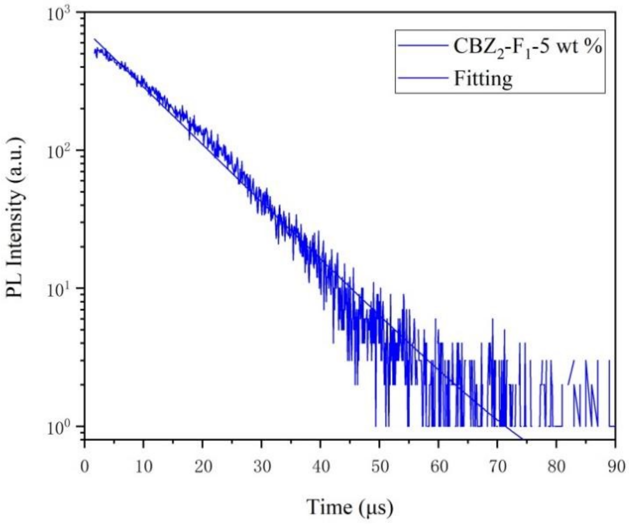


**Fig. S25** Fluorescence lifetime decay curve of **4g** in $CBZ_2$-$F_1$ film (5 wt %).

## VII. AIE assay

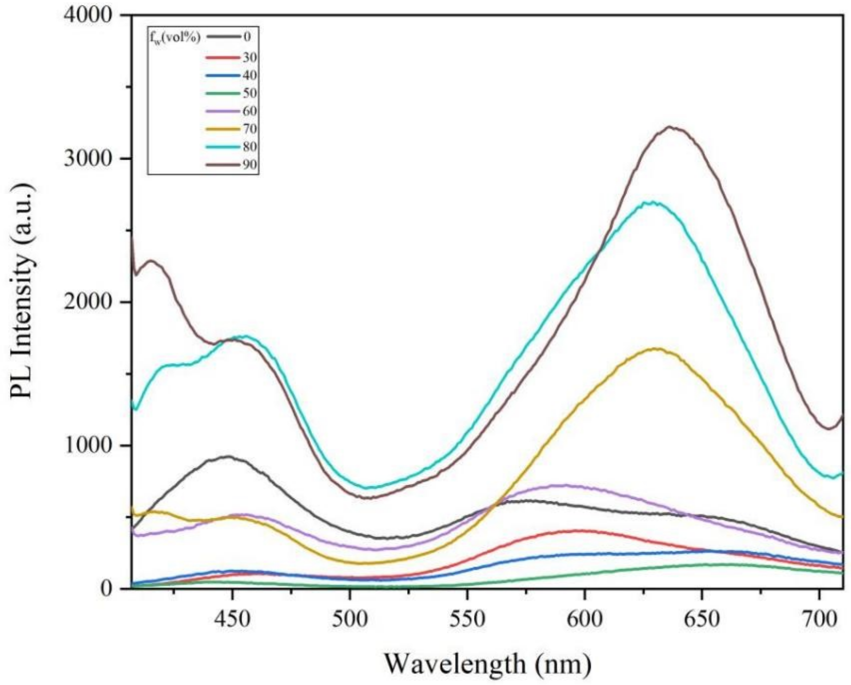


**Fig. S26** Photoluminescence spectra of **4g** in THF/Water mixtures with different Water fractions ($f_w$). (Concentration: 100 μM, excitation wavelength: 380 nm).

## VIII. ROS generation assays

The 0.4 mL of dichlorofluorescein (DCFH) stock solution (50 μM) was added to 1.6 mL of **4g** suspension with the final concentration of 10 μM. The photoluminescence (PL) spectra of DCFH were observed with excitation wavelength at 488 nm and emission spectra region was collected from 500 to 650 nm at various UV light irradiation time. The emission intensity at 525 nm was analyzed to demonstrate the generation rate of reactive oxygen species (ROS).

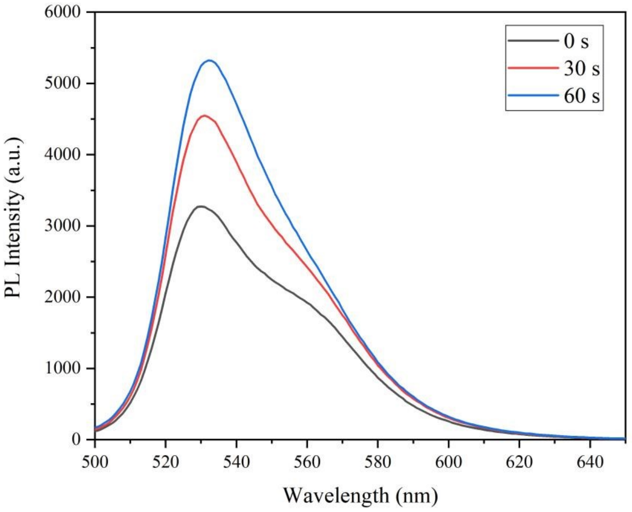


**Fig. S27** The photoluminescence (PL) spectra of DCFH in the **4g** aggregates under the irradiation at different time. Concentration: DCFH (50 μM), **4g** (10 μM), UV light irradiation.

# IX. Fluorescence emission spectra of 4g in different atmospheres

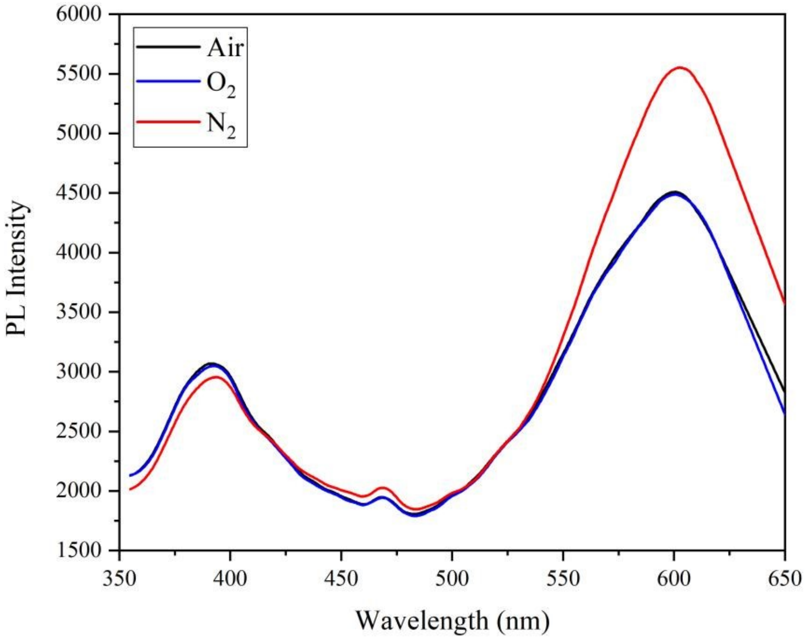

**Fig. S28** Fluorescence emission spectra of **4g** in PMMA film (0.15 wt %) in air, nitrogen, and oxygen at room temperature.

## X. Cyclic voltammogram experiment

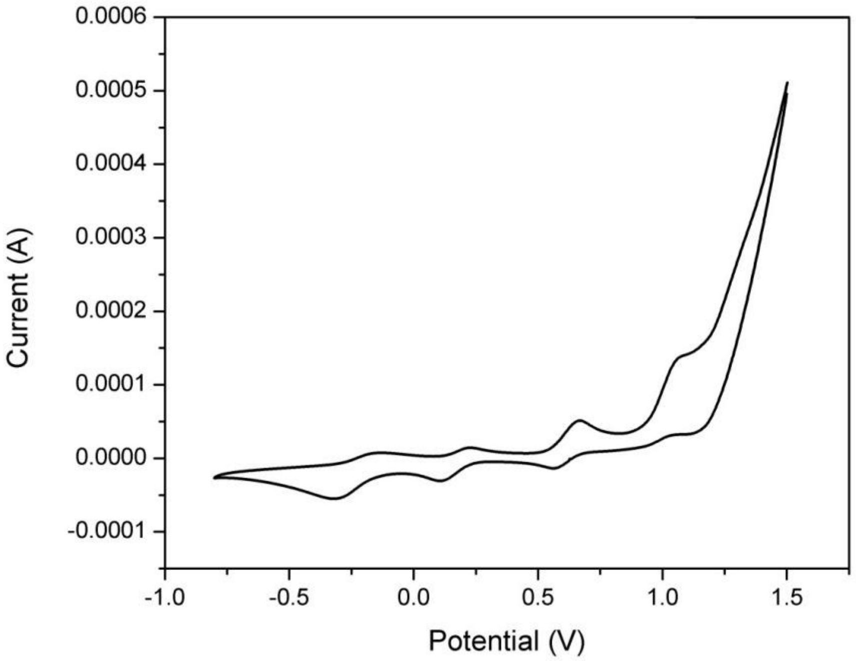


**Fig. S29** Cyclic voltammogram of **4g** in $CH_2Cl_2$.

**Table S3**. Energy levels of **4g**

| Compound | $E_g^{opt}$ (eV)[a] | $E_{on}^{ox}$(eV)[b] | HOMO (eV)[c] | LUMO (eV)[d] |
|---|---|---|---|---|
| **4g** | 1.94 | 0.38 | -5.18 | -3.24 |

[a] $E_g^{opt}$ = 1240/$\lambda_{onset}$ (eV). [b] Estimated from the oxidation onset of the cyclic voltammetry in 1.0 × $10^{-4}$ M DCM solution. [c] HOMO = −(4.8 + $E_{on}^{ox}$) (eV). [d] LUMO = (HOMO + $E_g^{opt}$) (eV).

## XI. DFT Calculation of 4g

All calculations were performed using the Gaussian 16, Revision A.03.[2-15] Geometries were optimized in dichloromethane with the CPCM solvation model by the B3-LYP functional and a basis set of 6-31G(d,p). Vibrational frequencies were calculated at the same level and check the optimized structure. Density functional

theory (DFT)[6,7] and time dependent DFT (TDDFT)[8,9-15] with the CAM-B3LYP functional and 6-31+G(d,p) basis set.[10,11] The D index is equal to the distance between the two Holes and electrons. The Sr index shows how much the hole and electron overlap.

Gaussian optimization + frequency:

# opt freq b3lyp/6-31g scrf = (cpcm, solvent = dichloromethane) geom = connectivity

**Cartesian coordinates of the structures**

| | | | |
|---|---|---|---|
| C | -0.52043 | 5.71402 | 0.56215 |
| C | -0.75265 | 4.3056 | 0.46929 |
| C | 0.36421 | 3.37622 | 0.5068 |
| C | 1.68051 | 3.90504 | 0.57864 |
| C | 1.87679 | 5.31037 | 0.65337 |
| C | 0.75641 | 6.19784 | 0.65619 |
| S | 3.17667 | 2.83541 | 0.72907 |
| C | 4.4088 | 3.94234 | -0.06857 |
| C | 4.28182 | 5.3268 | 0.05777 |
| N | 3.1406 | 5.88374 | 0.73 |
| C | 5.49408 | 3.36697 | -0.72534 |
| C | 6.49503 | 4.19448 | -1.2554 |
| C | 6.3725 | 5.5827 | -1.14829 |
| C | 5.26635 | 6.15465 | -0.50609 |
| C | 3.2929 | 7.23166 | 1.29907 |
| O | -1.657 | 6.49994 | 0.5197 |
| C | -2.05477 | 3.85796 | 0.31751 |
| N | -2.35416 | 2.54595 | 0.22113 |
| C | -1.337 | 1.57853 | 0.32049 |
| C | -0.00674 | 1.96448 | 0.47506 |
| C | -3.75479 | 2.18296 | 0.0292 |
| C | -1.75421 | 0.14821 | 0.24058 |
| C | 0.97787 | 0.84524 | 0.58428 |
| C | -4.49264 | 1.6136 | 1.07242 |
| C | -5.83592 | 1.30079 | 0.88316 |
| C | -6.47429 | 1.56393 | -0.35029 |
| C | -5.71696 | 2.15474 | -1.38553 |
| C | -4.36952 | 2.45579 | -1.19732 |
| C | -1.77672 | -0.6565 | 1.39298 |
| C | -2.08671 | -2.01221 | 1.31511 |
| C | -2.37607 | -2.60775 | 0.07173 |
| C | -2.3823 | -1.7976 | -1.0807 |
| C | -2.07584 | -0.4415 | -0.99435 |

| | | | |
|---|---|---|---|
| C | 1.5218 | 0.46002 | 1.82229 |
| C | 2.37745 | -0.63347 | 1.92874 |
| C | 2.71128 | -1.40898 | 0.79122 |
| C | 2.15405 | -1.02194 | -0.45315 |
| C | 1.31154 | 0.07735 | -0.54516 |
| C | -1.52559 | 7.94908 | 0.59127 |
| N | -2.62271 | -4.00243 | -0.03193 |
| N | 3.5516 | -2.52329 | 0.87773 |
| C | -2.01207 | -4.74428 | -1.09995 |
| C | 4.03799 | -3.19269 | -0.31338 |
| C | 4.05797 | -3.00539 | 2.13662 |
| C | -3.38279 | -4.6834 | 0.96754 |
| C | -4.54129 | -4.0908 | 1.50551 |
| C | -5.28234 | -4.75577 | 2.48536 |
| C | -4.89476 | -6.02515 | 2.93177 |
| C | -3.74884 | -6.62052 | 2.39044 |
| C | -2.99157 | -5.95668 | 1.42204 |
| C | 3.63958 | -4.51576 | -0.56135 |
| C | 4.12768 | -5.18766 | -1.68786 |
| C | 5.00653 | -4.53881 | -2.56653 |
| C | 5.40105 | -3.2187 | -2.31572 |
| C | 4.91886 | -2.54176 | -1.1876 |
| C | 5.44246 | -3.00568 | 2.37268 |
| C | 5.94238 | -3.5097 | 3.57745 |
| C | 5.07152 | -4.00412 | 4.55639 |
| C | 3.69098 | -3.99935 | 4.32226 |
| C | 3.18416 | -3.50955 | 3.11432 |
| C | -2.76038 | -5.68541 | -1.82786 |
| C | -2.15569 | -6.41565 | -2.85501 |
| C | -0.80849 | -6.20707 | -3.17642 |
| C | -0.06395 | -5.2646 | -2.45591 |
| C | -0.65733 | -4.54033 | -1.41808 |
| N | -7.83465 | 1.24216 | -0.54467 |
| C | -8.32611 | 0.89701 | -1.85085 |
| C | -8.75616 | 1.23784 | 0.55706 |
| C | -9.49754 | 1.49784 | -2.34127 |
| C | -9.99006 | 1.14606 | -3.60118 |
| C | -9.31491 | 0.20571 | -4.38884 |
| C | -8.14494 | -0.39037 | -3.90185 |
| C | -7.65485 | -0.05637 | -2.63624 |
| C | -9.63325 | 0.15448 | 0.73531 |
| C | -10.54702 | 0.15944 | 1.79281 |
| C | -10.58781 | 1.23353 | 2.69016 |
| C | -9.71148 | 2.31173 | 2.51538 |
| C | -8.80607 | 2.32218 | 1.45075 |
| H | 0.93296 | 7.26186 | 0.67785 |
| H | 5.5933 | 2.28484 | -0.80515 |
| H | 7.34918 | 3.72392 | -1.73231 |
| H | 7.1352 | 6.23188 | -1.56616 |
| H | 5.1767 | 7.23363 | -0.4553 |

| | | | |
|---|---|---|---|
| H | 4.31067 | 7.32681 | 1.6792 |
| H | 3.1206 | 8.0329 | 0.56661 |
| H | 2.60333 | 7.35827 | 2.13612 |
| H | -2.87829 | 4.55492 | 0.27474 |
| H | -4.01567 | 1.39721 | 2.02097 |
| H | -6.39651 | 0.84561 | 1.68961 |
| H | -6.1872 | 2.37541 | -2.33531 |
| H | -3.79718 | 2.9038 | -2.00303 |
| H | -1.50559 | -0.22821 | 2.35216 |
| H | -2.07356 | -2.62274 | 2.2101 |
| H | -2.60833 | -2.24316 | -2.04228 |
| H | -2.06802 | 0.15942 | -1.89817 |
| H | 1.28868 | 1.03127 | 2.71685 |
| H | 2.7973 | -0.88481 | 2.89363 |
| H | 2.40513 | -1.57585 | -1.34732 |
| H | 0.9124 | 0.35068 | -1.51671 |
| H | -2.5454 | 8.32738 | 0.54967 |
| H | -1.05274 | 8.25067 | 1.53182 |
| H | -0.95024 | 8.33032 | -0.25894 |
| H | -4.85609 | -3.11597 | 1.14981 |
| H | -6.17372 | -4.286 | 2.88983 |
| H | -5.47643 | -6.54215 | 3.68763 |
| H | -3.43336 | -7.60166 | 2.73114 |
| H | -2.09835 | -6.41749 | 1.0166 |
| H | 2.96591 | -5.01054 | 0.13128 |
| H | 3.82972 | -6.2159 | -1.87228 |
| H | 5.39063 | -5.06438 | -3.43563 |
| H | 6.10249 | -2.70295 | -2.96404 |
| H | 5.23958 | -1.52077 | -0.99209 |
| H | 6.11436 | -2.61219 | 1.61746 |
| H | 7.01336 | -3.50719 | 3.75275 |
| H | 5.46464 | -4.39108 | 5.4912 |
| H | 3.00969 | -4.38886 | 5.07272 |
| H | 2.11669 | -3.51788 | 2.91943 |
| H | -3.80559 | -5.84082 | -1.58369 |
| H | -2.74144 | -7.14132 | -3.41065 |
| H | -0.34378 | -6.77334 | -3.97673 |
| H | 0.98493 | -5.10274 | -2.68314 |
| H | -0.07311 | -3.82088 | -0.85457 |
| H | -10.0149 | 2.23217 | -1.73392 |
| H | -10.89637 | 1.61486 | -3.97066 |
| H | -9.69674 | -0.06151 | -5.36841 |
| H | -7.62032 | -1.12809 | -4.50038 |
| H | -6.75895 | -0.532 | -2.2523 |
| H | -9.59562 | -0.68124 | 0.0456 |
| H | -11.22038 | -0.68195 | 1.92054 |
| H | -11.29478 | 1.2319 | 3.51293 |
| H | -9.74228 | 3.15406 | 3.1991 |
| H | -8.1413 | 3.16666 | 1.30522 |
| S | 7.681 | 0.22981 | -1.75953 |

| | | | |
|---|---|---|---|
| O | 6.2554 | 0.35149 | -0.9279 |
| O | 8.44716 | 1.65918 | -2.01736 |
| C | 8.80643 | -0.65921 | -0.4075 |
| F | 10.04361 | -0.96631 | -0.92282 |
| F | 8.20003 | -1.8315 | 0.00857 |
| F | 8.97105 | 0.15774 | 0.6878 |
| O | 7.67151 | -0.83655 | -3.00709 |

**Gaussian calculation:**

# td = (singlets, nstates = 7, root = 1) cam-b3lyp/6-31+g(d,p) scrf = (cpcm, solvent = dichloromethane) geom = connectivity

**Cartesian coordinates of the structures**

| | | | |
|---|---|---|---|
| C | -0.20189 | 5.66194 | 0.63101 |
| C | -0.48184 | 4.26643 | 0.47059 |
| C | 0.59464 | 3.29697 | 0.4616 |
| C | 1.92717 | 3.78378 | 0.53793 |
| C | 2.1746 | 5.16788 | 0.7006 |
| C | 1.09289 | 6.09444 | 0.75464 |
| S | 3.40566 | 2.66635 | 0.53848 |
| C | 4.62513 | 3.80468 | -0.23267 |
| C | 4.55315 | 5.17513 | 0.03937 |
| N | 3.46775 | 5.6902 | 0.81036 |
| C | 5.66031 | 3.26917 | -0.99831 |
| C | 6.66977 | 4.11213 | -1.48353 |
| C | 6.60897 | 5.48437 | -1.22131 |
| C | 5.5512 | 6.02088 | -0.47664 |
| C | 3.66459 | 6.99397 | 1.47166 |
| O | -1.3029 | 6.48332 | 0.62657 |
| C | -1.80027 | 3.86975 | 0.30651 |
| N | -2.14293 | 2.57544 | 0.16651 |
| C | -1.16731 | 1.56876 | 0.23687 |
| C | 0.17529 | 1.90317 | 0.39829 |
| C | -3.56189 | 2.26859 | -0.01373 |
| C | -1.6557 | 0.16195 | 0.14706 |
| C | 1.12327 | 0.7546 | 0.54663 |
| C | -4.31922 | 1.78941 | 1.05988 |
| C | -5.67628 | 1.52769 | 0.88539 |
| C | -6.30228 | 1.75644 | -0.36067 |
| C | -5.52247 | 2.25929 | -1.42489 |
| C | -4.16185 | 2.50891 | -1.25362 |
| C | -1.70161 | -0.65596 | 1.28983 |
| C | -2.15117 | -1.97259 | 1.21092 |
| C | -2.57733 | -2.51293 | -0.01995 |

| | | | |
|---|---|---|---|
| C | -2.53697 | -1.69146 | -1.16533 |
| C | -2.07979 | -0.37772 | -1.08023 |
| C | 1.66357 | 0.42791 | 1.8033 |
| C | 2.48425 | -0.6865 | 1.97161 |
| C | 2.77837 | -1.54106 | 0.88409 |
| C | 2.22654 | -1.21578 | -0.3781 |
| C | 1.42102 | -0.09206 | -0.5357 |
| C | -1.12722 | 7.92997 | 0.77972 |
| N | -3.0315 | -3.85444 | -0.10652 |
| N | 3.58307 | -2.68462 | 1.05105 |
| C | -2.75462 | -4.63723 | -1.27599 |
| C | 4.13926 | -3.39444 | -0.07531 |
| C | 3.88863 | -3.20619 | 2.35725 |
| C | -3.75978 | -4.44711 | 0.97602 |
| C | -4.79392 | -3.73729 | 1.61429 |
| C | -5.50047 | -4.32247 | 2.6692 |
| C | -5.20065 | -5.62503 | 3.08979 |
| C | -4.1783 | -6.33643 | 2.44818 |
| C | -3.45547 | -5.75241 | 1.40365 |
| C | 3.86384 | -4.76438 | -0.22572 |
| C | 4.42997 | -5.48149 | -1.28471 |
| C | 5.26762 | -4.83642 | -2.20431 |
| C | 5.53905 | -3.46978 | -2.05625 |
| C | 4.98093 | -2.74777 | -0.99446 |
| C | 5.23116 | -3.36957 | 2.73883 |
| C | 5.54292 | -3.91103 | 3.99029 |
| C | 4.52172 | -4.28098 | 4.87536 |
| C | 3.18283 | -4.11231 | 4.49762 |
| C | 2.86406 | -3.58525 | 3.24171 |
| C | -3.76663 | -5.4213 | -1.85859 |
| C | -3.4911 | -6.19704 | -2.98877 |
| C | -2.21212 | -6.18957 | -3.56073 |
| C | -1.20496 | -5.40376 | -2.98489 |
| C | -1.46765 | -4.63839 | -1.84478 |
| N | -7.67871 | 1.48812 | -0.5385 |
| C | -8.1839 | 1.07402 | -1.81776 |
| C | -8.59844 | 1.61272 | 0.55727 |
| C | -9.3384 | 1.67695 | -2.34678 |
| C | -9.84503 | 1.25902 | -3.58131 |
| C | -9.20049 | 0.249 | -4.30736 |
| C | -8.04667 | -0.34855 | -3.78288 |
| C | -7.54347 | 0.05206 | -2.54143 |
| C | -9.53214 | 0.59288 | 0.81165 |
| C | -10.44295 | 0.72227 | 1.86466 |
| C | -10.42431 | 1.85915 | 2.68318 |
| C | -9.49065 | 2.87364 | 2.43327 |
| C | -8.58779 | 2.75936 | 1.37173 |
| H | 1.30709 | 7.14785 | 0.83977 |
| H | 5.7051 | 2.20213 | -1.19684 |
| H | 7.49016 | 3.68382 | -2.04924 |

| | | | |
|---|---|---|---|
| H | 7.37991 | 6.14799 | -1.59851 |
| H | 5.50882 | 7.08902 | -0.3049 |
| H | 4.6852 | 7.03364 | 1.85446 |
| H | 3.50992 | 7.84621 | 0.79792 |
| H | 2.98152 | 7.07888 | 2.31758 |
| H | -2.59942 | 4.59523 | 0.29105 |
| H | -3.852 | 1.60825 | 2.02071 |
| H | -6.25451 | 1.14268 | 1.71554 |
| H | -5.98345 | 2.45666 | -2.38418 |
| H | -3.57286 | 2.89333 | -2.07908 |
| H | -1.36618 | -0.26636 | 2.24477 |
| H | -2.16594 | -2.58842 | 2.10192 |
| H | -2.86332 | -2.08489 | -2.12037 |
| H | -2.05612 | 0.23267 | -1.97673 |
| H | 1.44618 | 1.05403 | 2.66343 |
| H | 2.89306 | -0.89759 | 2.95116 |
| H | 2.42804 | -1.84755 | -1.23337 |
| H | 1.01175 | 0.12694 | -1.51633 |
| H | -2.13562 | 8.33698 | 0.75546 |
| H | -0.65033 | 8.15833 | 1.7369 |
| H | -0.53695 | 8.33402 | -0.0474 |
| H | -5.0407 | -2.73501 | 1.28199 |
| H | -6.29574 | -3.76414 | 3.15279 |
| H | -5.75564 | -6.07875 | 3.90404 |
| H | -3.9329 | -7.34412 | 2.76802 |
| H | -2.6563 | -6.30145 | 0.91855 |
| H | 3.21415 | -5.25931 | 0.48833 |
| H | 4.21373 | -6.53976 | -1.39229 |
| H | 5.70898 | -5.39432 | -3.02395 |
| H | 6.20847 | -2.96358 | -2.74428 |
| H | 5.21434 | -1.69479 | -0.88279 |
| H | 6.01852 | -3.06934 | 2.05534 |
| H | 6.58242 | -4.03452 | 4.27653 |
| H | 4.76592 | -4.69674 | 5.84734 |
| H | 2.3857 | -4.40328 | 5.17426 |
| H | 1.82821 | -3.46784 | 2.94219 |
| H | -4.76076 | -5.41936 | -1.42565 |
| H | -4.28053 | -6.79799 | -3.42879 |
| H | -2.00284 | -6.78787 | -4.44116 |
| H | -0.2081 | -5.39632 | -3.41389 |
| H | -0.68083 | -4.04435 | -1.39315 |
| H | -9.83163 | 2.46685 | -1.79131 |
| H | -10.73713 | 1.73123 | -3.97991 |
| H | -9.59246 | -0.06935 | -5.26749 |
| H | -7.5446 | -1.13808 | -4.33266 |
| H | -6.66013 | -0.42429 | -2.13056 |
| H | -9.54008 | -0.29296 | 0.18642 |
| H | -11.15932 | -0.07121 | 2.05141 |
| H | -11.1284 | 1.95423 | 3.50295 |
| H | -9.47343 | 3.76277 | 3.05525 |

| | | | |
|---|---|---|---|
| H | -7.87862 | 3.55452 | 1.16972 |
| S | 7.99146 | -0.06694 | -2.09697 |
| O | 6.38311 | 0.19197 | -1.83997 |
| O | 8.87997 | 1.2915 | -2.363 |
| C | 8.6119 | -0.66186 | -0.32708 |
| F | 9.95838 | -0.93315 | -0.37247 |
| F | 7.93614 | -1.79963 | 0.05244 |
| F | 8.38086 | 0.32765 | 0.59813 |
| O | 8.3413 | -1.34222 | -3.07415 |

**Gaussian calculation:**

# tda = (triplets, nstates = 1) uhf/6-311g geom=connectivity

**Cartesian coordinates of the structures**

| | | | |
|---|---|---|---|
| C | -1.10287 | 5.19825 | -0.40249 |
| C | -0.66763 | 3.93631 | -0.32732 |
| C | -1.54484 | 2.92918 | -0.26542 |
| C | -2.85725 | 3.1839 | -0.2787 |
| C | -3.29249 | 4.44584 | -0.35387 |
| C | -2.4223 | 5.45854 | -0.28334 |
| S | -4.09496 | 1.86003 | -0.18148 |
| C | -5.47871 | 2.85348 | 0.44519 |
| C | -5.48603 | 4.16233 | 0.17229 |
| N | -4.54592 | 4.67297 | -0.50472 |
| C | -6.46542 | 2.31626 | 1.17006 |
| C | -7.46568 | 3.08566 | 1.61164 |
| C | -7.46693 | 4.39657 | 1.34863 |
| C | -6.48013 | 4.93383 | 0.62396 |
| C | -4.92742 | 5.61664 | -1.56526 |
| O | -0.2241 | 6.21102 | -0.59726 |
| C | 0.64471 | 3.68225 | -0.30246 |
| N | 1.068 | 2.45527 | -0.22937 |
| C | 0.20295 | 1.41392 | -0.15432 |
| C | -1.10948 | 1.66797 | -0.17868 |
| C | 2.34691 | 2.22186 | -0.22825 |
| C | 0.65306 | 0.1587 | -0.05784 |
| C | -1.98669 | 0.66087 | -0.11638 |
| C | 2.85029 | 1.09243 | -0.87363 |
| C | 4.2225 | 0.84205 | -0.87234 |
| C | 5.09124 | 1.72106 | -0.22575 |
| C | 4.58786 | 2.85049 | 0.41963 |
| C | 3.21565 | 3.10087 | 0.41833 |
| C | 0.22315 | -0.80812 | -0.96661 |
| C | 0.69268 | -2.11774 | -0.86589 |
| C | 1.59219 | -2.4604 | 0.1436 |
| C | 2.0221 | -1.49357 | 1.05237 |

| | | | |
|---|---|---|---|
| C | 1.55247 | -0.18399 | 0.95164 |
| C | -2.59141 | 0.18965 | -1.28158 |
| C | -3.50658 | -0.861 | -1.21651 |
| C | -3.81684 | -1.44036 | 0.01375 |
| C | -3.21209 | -0.96923 | 1.17895 |
| C | -2.29696 | 0.08151 | 1.11387 |
| C | -0.88866 | 7.44469 | -0.64453 |
| N | 2.01834 | -3.64896 | 0.23501 |
| N | -4.64746 | -2.39397 | 0.07275 |
| C | 2.8348 | -3.96002 | 1.15126 |
| C | -4.9292 | -2.91993 | 1.18946 |
| C | -5.19643 | -2.82165 | -0.98485 |
| C | 1.62815 | -4.52652 | -0.58982 |
| C | 1.06879 | -5.71784 | -0.12805 |
| C | 0.63891 | -6.68476 | -1.03692 |
| C | 0.76836 | -6.46027 | -2.40745 |
| C | 1.3276 | -5.2689 | -2.86923 |
| C | 1.75757 | -4.30194 | -1.96037 |
| C | -4.80327 | -4.29817 | 1.36276 |
| C | -5.11372 | -4.87759 | 2.59302 |
| C | -5.55004 | -4.07866 | 3.64987 |
| C | -5.67588 | -2.70039 | 3.47656 |
| C | -5.36552 | -2.121 | 2.24631 |
| C | -6.58676 | -2.89506 | -1.06824 |
| C | -7.19157 | -3.36632 | -2.23353 |
| C | -6.40592 | -3.76422 | -3.31512 |
| C | -5.01559 | -3.69081 | -3.23173 |
| C | -4.41078 | -3.21954 | -2.06644 |
| C | 4.04288 | -4.57799 | 0.82858 |
| C | 4.94233 | -4.92078 | 1.83807 |
| C | 4.63365 | -4.64551 | 3.17014 |
| C | 3.42563 | -4.02742 | 3.49281 |
| C | 2.52609 | -3.68467 | 2.48332 |
| N | 6.33664 | 1.49379 | -0.22454 |
| C | 7.12514 | 2.29156 | 0.36233 |
| C | 6.79347 | 0.46861 | -0.8103 |
| C | 8.20527 | 2.83944 | -0.32946 |
| C | 9.07414 | 3.71839 | 0.31723 |
| C | 8.86275 | 4.04952 | 1.6556 |
| C | 7.78253 | 3.5016 | 2.34739 |
| C | 6.91366 | 2.62265 | 1.70071 |
| C | 7.60917 | -0.42535 | -0.11678 |
| C | 8.11264 | -1.55475 | -0.76217 |
| C | 7.80032 | -1.79021 | -2.10107 |
| C | 6.98455 | -0.89638 | -2.79448 |
| C | 6.48114 | 0.23315 | -2.14911 |
| H | -2.77624 | 6.48875 | -0.13053 |
| H | -6.45422 | 1.24136 | 1.40305 |
| H | -8.2876 | 2.64029 | 2.19138 |
| H | -8.27977 | 5.03294 | 1.72839 |

| | | | |
|---|---|---|---|
| H | -6.48611 | 6.01071 | 0.39936 |
| H | -6.03519 | 5.72206 | -1.58801 |
| H | -4.46502 | 6.60855 | -1.36252 |
| H | -4.57108 | 5.23412 | -2.54783 |
| H | 1.36639 | 4.51141 | -0.3439 |
| H | 2.16513 | 0.3993 | -1.38371 |
| H | 4.61952 | -0.04873 | -1.38123 |
| H | 5.27299 | 3.5437 | 0.9295 |
| H | 2.81866 | 3.99156 | 0.92733 |
| H | -0.48611 | -0.5378 | -1.76276 |
| H | 0.35367 | -2.88019 | -1.58251 |
| H | 2.73155 | -1.76384 | 1.84842 |
| H | 1.89146 | 0.57857 | 1.66836 |
| H | -2.34654 | 0.64646 | -2.25181 |
| H | -3.9835 | -1.23268 | -2.13543 |
| H | -3.45681 | -1.42619 | 2.14919 |
| H | -1.82003 | 0.4532 | 2.03279 |
| H | -0.14797 | 8.25975 | -0.80496 |
| H | -1.62073 | 7.43913 | -1.48287 |
| H | -1.42448 | 7.6143 | 0.31616 |
| H | 0.96662 | -5.89487 | 0.95277 |
| H | 0.19789 | -7.62441 | -0.6728 |
| H | 0.42935 | -7.22273 | -3.12408 |
| H | 1.42961 | -5.09171 | -3.95007 |
| H | 2.19863 | -3.36238 | -2.32448 |
| H | -4.45906 | -4.92815 | 0.52932 |
| H | -5.01444 | -5.96451 | 2.72972 |
| H | -5.79485 | -4.53567 | 4.62002 |
| H | -6.01987 | -2.07044 | 4.31011 |
| H | -5.4648 | -1.03408 | 2.1095 |
| H | -7.20637 | -2.58119 | -0.21527 |
| H | -8.28804 | -3.42432 | -2.29937 |
| H | -6.88294 | -4.13594 | -4.23404 |
| H | -4.39604 | -4.0045 | -4.08481 |
| H | -3.31428 | -3.16164 | -2.0006 |
| H | 4.28629 | -4.795 | -0.22197 |
| H | 5.89503 | -5.40821 | 1.58368 |
| H | 5.34292 | -4.91584 | 3.96629 |
| H | 3.18219 | -3.81032 | 4.54335 |
| H | 1.57333 | -3.19736 | 2.73772 |
| H | 8.37199 | 2.57842 | -1.38496 |
| H | 9.92602 | 4.1505 | -0.2282 |
| H | 9.54791 | 4.74264 | 2.16558 |
| H | 7.61574 | 3.76281 | 3.40278 |
| H | 6.06182 | 2.19045 | 2.24624 |
| H | 7.85544 | -0.23969 | 0.9391 |
| H | 8.75599 | -2.25975 | -0.21528 |
| H | 8.19743 | -2.68096 | -2.60997 |
| H | 6.73822 | -1.08216 | -3.85036 |
| H | 5.83788 | 0.93817 | -2.69609 |

Transition dipole moments (TDMs) and their derivatives were extracted from the frequency calculations, using Multiwfn version 3.7 for those between excited states.[19]

**S1**

Integral of hole: 0.931096
Integral of electron: 0.928180
Integral of transition density: -0.001371
Transition dipole moment in X/Y/Z: -1.411740 -1.726662 -0.103818 a.u.
Sm index (integral of Sm function): 0.23393 a.u.
Sr index (integral of Sr function): 0.47779 a.u.
Centroid of hole in X/Y/Z: 2.889367 3.304729 0.390228 Angstrom Centroid of electron in X/Y/Z: -0.255594 3.893796 0.413279 Angstrom
Dx: 3.145 Dy: 0.589 Dz: 0.023 D index: 3.200 Angstrom
Variation of dipole moment with respect to ground state:
X: 5.524944 Y: -1.034851 Z: -0.040494 Norm: 5.621171 a.u.
RMSD of hole in X/Y/Z: 2.225 2.360 1.061 Norm: 3.413 Angstrom
RMSD of electron in X/Y/Z: 2.164 1.574 0.865 Norm: 2.812 Angstrom
Difference between RMSD of hole and electron (delta sigma):
X: -0.062 Y: -0.786 Z: -0.195 Overall: -0.601 Angstrom
H_x: 2.195 H_y: 1.967 H_z: 0.963 H_CT: 2.187 H index: 3.112 Angstrom
t index: 1.012 Angstrom
Hole delocalization index (HDI): 6.31
Electron delocalization index (EDI): 7.84
Ghost-hunter index (def 1): 4.675 eV, 1st/2nd terms: 9.175 4.500 eV
Ghost-hunter index (def 2): 3.952 eV, 1st/2nd terms: 8.452 4.500 eV
Excitation energy of this state: 2.774 eV

**S2**

Integral of hole: 0.858858
Integral of electron: 0.858852
Integral of transition density: 0.000360
Transition dipole moment in X/Y/Z: 0.636377 -0.148276 -0.021113 a.u.
Sm index (integral of Sm function): 0.09154 a.u.
Sr index (integral of Sr function): 0.26810 a.u.
Centroid of hole in X/Y/Z: 0.605906 -1.289106 0.477553 Angstrom
Centroid of electron in X/Y/Z: -0.223648 3.771941 0.406038 Angstrom
Dx: 0.830 Dy: 5.061 Dz: 0.072 D index: 5.129 Angstrom
Variation of dipole moment with respect to ground state:
X: 1.346368 Y: -8.214082 Z: 0.116070 Norm: 8.324501 a.u.
RMSD of hole in X/Y/Z: 4.131 2.954 1.682 Norm: 5.350 Angstrom
RMSD of electron in X/Y/Z: 2.170 1.648 0.870 Norm: 2.860 Angstrom
Difference between RMSD of hole and electron (delta sigma):
X: -1.962 Y: -1.306 Z: -0.812 Overall: -2.490 Angstrom
H_x: 3.151 H_y: 2.301 H_z: 1.276 H_CT: 2.327 H index: 4.105 Angstrom
t index: 2.802 Angstrom
Hole delocalization index (HDI): 5.06

Electron delocalization index (EDI): 7.07
Ghost-hunter index (def 1): 5.844 eV, 1st/2nd terms: 8.652 2.807 eV
Ghost-hunter index (def 2): 5.576 eV, 1st/2nd terms: 8.383 2.807 eV
Excitation energy of this state: 3.398 eV

# XII. Nanoparticle preparation and characterization

### i. Preparation of nanoparticles 4g

The **4g** (0.5 mg) compounds were separately dissolved in 3 mL of THF and then ultrasound for 30 min. Then, 30 mg of Poloxamer 188 was added and the mixed solution was treated by ultrasound for 5 min, followed by stirring at room temperature for 6 h. Afterwards, the solvent was removed by reduced pressure and 3 mL of deionized water was added to drive the formation of NPs after ultrasound for 6 h. To remove the residual THF, the nanoparticle suspensions were dialyzed by 3 times (molecular weight cut-off 3500). The concentrations of nanoparticles were calibrated via the UV-vis spectra. Finally, the NPs solution was collected and stored at 4 °C.

### ii. Dynamic light scattering (DLS) measurement

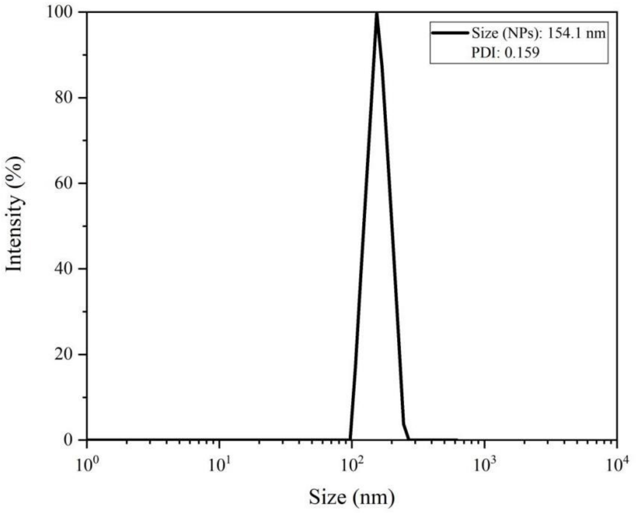

**Fig. S30** Hydrodynamic size distribution of **4g** nanoparticles in aqueous solution.

### iii. The photophysical properties of 4g nanoparticles

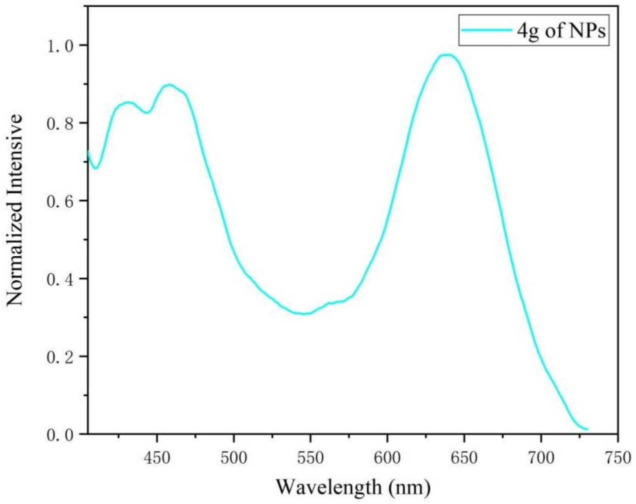


**Fig. S31** Normalized fluorescence emission spectra of **4g** nanoparticles**.**

## XIII. Cell imaging experiments and MTT assays

### i. Methyl thiazolyl tetrazolium (MTT) assay

To assess the safe usability of **4g** nanoparticles for biomedical applications, the cytotoxicity experiments in HeLa cells were carried out using the standard methyl thiazolyl tetrazolium (MTT) assay.

**Cellular Experiments:** The HeLa cells were cultured in 10% FBS and 1% penicillin-streptomycin-containing DMEM medium at 37 °C with 5% $CO_2$.

**In Vitro Cytotoxicity:** The bioavailabilities of **4g** NPs in HeLa cells were measured via MTT assays. The HeLa cells were seeded into 96-well plates and incubated with a standard medium overnight. Then, the cells were treated with **4g** NPs separately at serial concentrations and incubated for another 24 h.

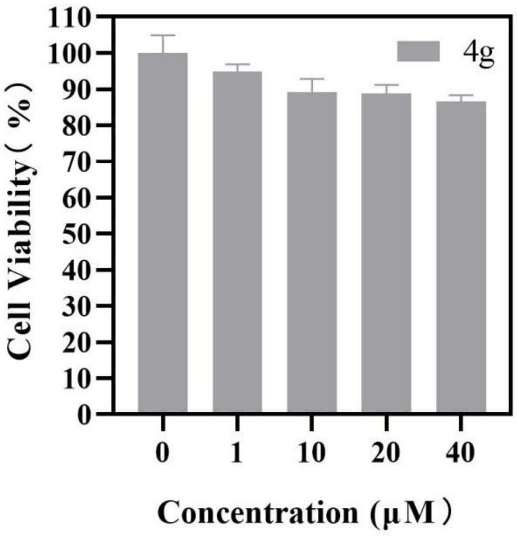


**Fig. S32** MTT assay for estimating cell viability (%) of HeLa cells was incubated with **4g** nanoparticles for 24 h.

**ii. Cell membrane-specific cell imaging**

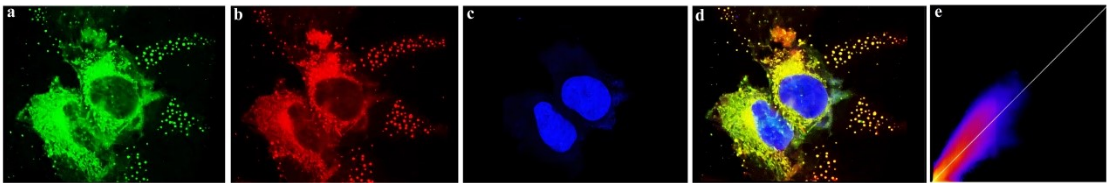


**Fig. S33 Co-staining of HeLa cells with 4g NPs and DiO to incubate for 30 min.** (a) Fluorescent image of HeLa cells with the DiO-stained membrane signals (5 μM, $\lambda_{ex}$ = 488 nm, $\lambda_{em}$ = 500-540 nm). (b) Fluorescent image of HeLa cells cultured with **4g** NPs (10 μM) ($\lambda_{ex}$ = 561 nm, $\lambda_{em}$ = 600–750 nm). (c) The nuclei are stained by DAPI (4',6-diamidino-2-phenylindole) and excited by a laser at 408 nm. (d) Merged image of (a), (b) and (c). (e) The Pearson correlation coefficient r = 0.90.

**iii. Mitochondria-specific cell imaging**

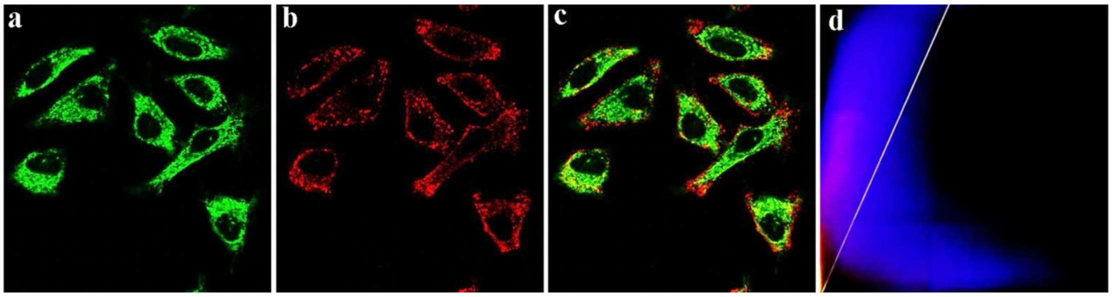


**Fig. S34 Co-staining of HeLa cells with 4g NPs and Mito-Tracker green (0.25 μM) to incubate for 15 min.** (a) fluorescent image of HeLa cells stained with Mito-Tracker green (0.25 μM, $\lambda_{ex}$ = 488 nm, $\lambda_{em}$ = 500–550 nm); (b) fluorescent image of HeLa cells stained with **4g** NPs (10 μM, $\lambda_{ex}$ = 556 nm, $\lambda_{em}$ = 600–750 nm); (c) merged images of (a) and (b); (d) Pearson's coefficient = 0.48.

**V. Lysosomes-specific cell imaging**

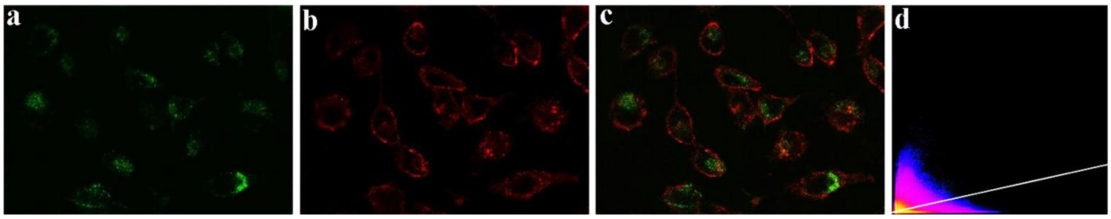


**Fig. S35 Co-staining of HeLa cells with 4g NPs and Lyso-Tracker green (0.25 μM) to incubate for 15 min.** (a) fluorescent image of HeLa cells stained with Lyso-Tracker green (0.25 μM, $\lambda_{ex}$ = 488 nm, $\lambda_{em}$ = 500–550 nm); (b) fluorescent image of HeLa cells stained with **4g** NPs (10 μM, $\lambda_{ex}$ = 556 nm, $\lambda_{em}$ = 600–750 nm); (c) merged images of (a) and (b); (d) Pearson's coefficient = 0.27.

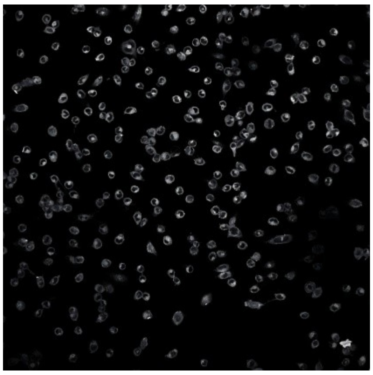

**Fig. S36** The white light emission signal in HeLa cells at a microscope under blue light excitation.

# XIV. Experimental data for the substrates 1a and 1b

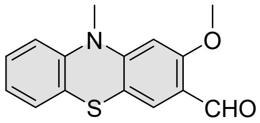


**2-methoxy-10-methyl-10*H*-phenothiazine-3-carbaldehyde (1a)**

Yellow solid. $^{1}H$ NMR (400 MHz, $CDCl_3$): $\delta$ (ppm) 10.20 (s, 1H), 7.54 (s, 1H), 7.18-7.11 (m, 2H), 6.97 (t, $J$ = 7.4 Hz, 1H), 6.82 (d, $J$ = 8.0 Hz, 1H), 6.29 (s, 1H), 3.91 (s, 3H), 3.43 (s, 3H); $^{13}C$ NMR (100 MHz, $CDCl_3$): $\delta$ (ppm) 187.41, 162.94, 152.88, 143.82, 127.60, 127.36, 126.70, 123.73, 123.46, 119.71, 114.94, 114.72, 97.74, 55.90, 36.05.

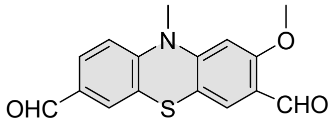


**2-methoxy-10-methyl-10*H*-phenothiazine-3,7-dicarbaldehyde (1b)**

Yellow solid. $^{1}H$ NMR (400 MHz, $CDCl_3$): $\delta$ (ppm) 10.25 (s, 1H), 9.84 (s, 1H), 7.69 (d, $J$ = 8.3 Hz, 1H), 7.62 (s, 1H), 7.57 (s, 1H), 6.93 (d, $J$ = 8.4 Hz, 1H), 6.38 (s, 1H), 3.95 (s, 3H), 3.52 (s, 3H); $^{13}C$ NMR (100 MHz, $CDCl_3$): $\delta$ (ppm) 190.19, 187.41, 130.12, 128.45, 127.10, 114.81, 98.72, 56.06, 36.58.

# XV. Experimental data for the described substances

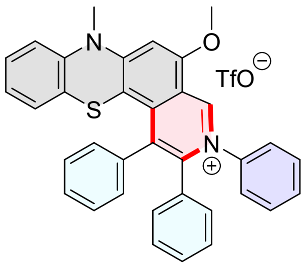


**5-methoxy-7-methyl-1,2,3-triphenyl-7*H*-pyrido[3,4-*c*]phenothiazin-3-ium trifluoromethanesulfonate (4a)**

Red solid 62 mg, yield: 92%. $^1$H NMR (400 MHz, CDCl$_3$): $\delta$ (ppm) 9.28 (s, 1H), 7.35-7.32 (m, 5H), 7.29 (d, $J$ = 7.2 Hz, 1H), 7.21 (t, $J$ = 7.2 Hz, 2H), 7.15-7.13 (m, 3H), 7.04 (s, 1H), 7.01-6.99 (m, 5H), 6.94 (t, $J$ = 6.8 Hz, 2H), 6.56 (d, $J$ = 7.6 Hz, 1H), 4.20 (s, 3H), 3.68 (s, 3H); $^{13}$C NMR (100 MHz, CDCl$_3$): $\delta$ (ppm) 159.89, 159.68, 145.54, 144.16, 143.01, 142.14, 135.96, 135.06, 134.76, 131.54, 131.47, 131.35, 130.24, 129.65, 128.91, 128.59, 128.17, 128.06, 127.76, 127.48, 127.06, 125.14, 123.94, 116.96, 115.81, 108.59, 100.37, 57.60, 38.07. Anal. Calcd. For. $C_{36}H_{27}F_3N_2O_4S_2$: C, 64.27; H, 4.05; N, 4.16; S, 9.53. Found: C, 64.51; H, 4.133; N, 4.16; S, 9.615. HRMS [ESI$^+$]: calcd for $C_{35}H_{27}N_2^+OS$, 523.1839, found 523.1855.

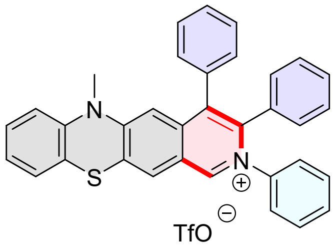


**6-methyl-2,3,4-triphenyl-6*H*-pyrido[4,3-*b*]phenothiazin-2-ium trifluoromethanesulfonate (4b)**

Yellow solid 41 mg, yield: 63%. $^{1}H$ NMR (400 MHz, $CDCl_3$): $\delta$ (ppm) 9.27 (s, 1H), 8.05 (s, 1H), 7.45-7.42 (m, 2H), 7.33-7.30 (m, 6H), 7.23-7.22 (m, 2H), 7.18 (t, $J$ = 8.6 Hz, 1H), 7.07-6.99 (m, 7H), 6.82 (d, $J$ = 8.4 Hz, 1H), 6.55 (s, 1H), 3.24 (s, 3H); $^{13}C$ NMR (100 MHz, $CDCl_3$): $\delta$ (ppm) 152.71, 146.28, 144.37, 142.05, 141.29, 140.63, 136.26, 133.58, 132.28, 131.50, 131.17, 130.25, 130.13, 129.58, 129.08, 128.83, 128.78, 128.49, 127.97, 127.20, 126.96, 126.51, 124.78, 123.67, 121.26, 116.56, 106.12, 36.68. HRMS [ESI$^+$]: calcd for $C_{34}H_{25}N_2^{+}S$, 493.1733, found 493.1733.

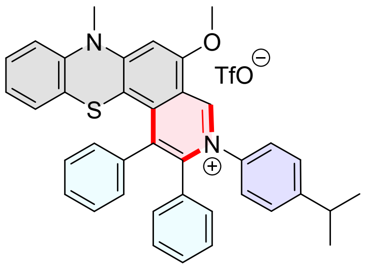


**3-(4-isopropylphenyl)-5-methoxy-7-methyl-1,2-diphenyl-7*H*-pyrido[3,4-*c*]phenothiazin-3-ium trifluoromethanesulfonate (4c)**

Red solid 69 mg, yield: 97%. $^{1}H$ NMR (400 MHz, $CDCl_3$): $\delta$ (ppm) 9.29 (s, 1H), 7.31 (t, $J$ = 7.4 Hz, 1H), 7.24-7.20 (m, 4H), 7.18-7.12 (m, 5H), 7.06 (s, 1H), 7.03-6.98 (m, 3H), 6.96-6.95 (m, 4H), 6.57 (d, $J$ = 8.0 Hz, 1H), 4.23 (s, 3H), 3.71 (s, 3H), 2.90-2.80 (m, 1H), 1.16 (d, $J$ = 6.8 Hz, 6H); $^{13}C$ NMR (100 MHz, $CDCl_3$): $\delta$ (ppm) 159.80, 159.65, 151.37, 145.62, 144.17, 143.01, 139.81, 135.96, 134.97, 134.66, 131.53, 131.42, 131.26, 128.85, 128.59, 128.18, 128.07, 127.72, 127.57, 127.46, 126.78, 125.12, 123.87, 116.92, 115.82, 108.47, 100.40, 57.62, 38.09, 33.82, 23.74. HRMS [ESI$^+$]: calcd for $C_{38}H_{33}N_2^{+}OS$, 565.2309, found 565.2324.

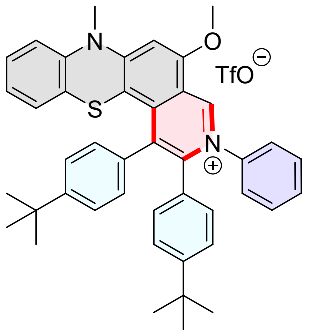


**1,2-bis(4-(tert-butyl)phenyl)-5-methoxy-7-methyl-3-phenyl-7*H*-pyrido[3,4-*c*]phenothiazin-3-ium trifluoromethanesulfonate (4d)**

Red solid 72 mg, yield: 92%. $^{1}H$ NMR (400 MHz, $CDCl_3$): $\delta$ (ppm) 9.27 (s, 1H), 7.34-7.33 (m, 5H), 7.21-7.15 (m, 3H), 7.06 (s, 1H), 6.98-6.92(m, 6H), 6.80 (d, $J$ = 8.0 Hz, 2H), 6.51 (d, $J$ = 7.6 Hz, 1H), 4.23 (s, 3H), 3.72 (s, 3H), 1.30 (s, 9H), 1.10 (s, 9H); $^{13}C$ NMR (100 MHz, $CDCl_3$): $\delta$ (ppm) 159.84, 159.59, 152.09, 145.86, 144.20, 142.61, 142.23, 135.16, 135.05, 132.90, 131.01, 130.98, 130.19, 129.64, 128.50, 128.16, 127.29, 126.95, 125.08, 124.72, 124.48, 124.20, 116.91, 115.88, 108.53, 100.40, 57.66, 38.12, 34.76, 34.60, 31.50, 31.01. HRMS [ESI$^+$]: calcd for $C_{43}H_{43}N_2^+OS$, 635.3091, found 635.3107.

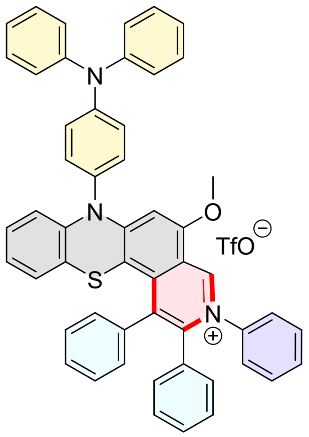

**7-(4-(diphenylamino)phenyl)-5-methoxy-1,2,3-triphenyl-7*H*-pyrido[3,4-*c*]phenothiazin-3-ium trifluoromethanesulfonate (4e)**

Black solid 80 mg, yield: 89%. $^{1}$H NMR (400 MHz, $CDCl_3$): $\delta$ (ppm) 9.22 (s, 1H), 7.41-7.39 (m, 2H), 7.35 (t, $J$ = 7.6 Hz, 4H), 7.29-7.25 (m, 7H), 7.23-7.19 (m, 9H), 7.14 (t, $J$ = 7.4 Hz, 2H), 7.00-6.99 (m, 5H), 6.94 (t, $J$ = 7.8 Hz, 1H), 6.87 (t, $J$ = 7.4 Hz, 1H), 6.44 (t, $J$ = 8.8 Hz, 3H), 3.86 (s, 3H); $^{13}$C NMR (100 MHz, $CDCl_3$): $\delta$ (ppm) 158.77, 157.80, 149.09, 146.91, 145.82, 142.83, 142.27, 142.14, 135.88, 135.54, 134.71, 131.91, 131.63, 131.42, 130.81, 130.07, 129.83, 129.55, 128.80, 128.52, 127.88, 127.68, 127.58, 127.37, 127.17, 125.57, 124.50, 123.35, 121.84, 117.44, 117.39, 104.53, 100.68, 56.38. HRMS [ESI$^+$]: calcd for $C_{52}H_{38}N_3^{+}OS$, 752.2731, found 752.2745.

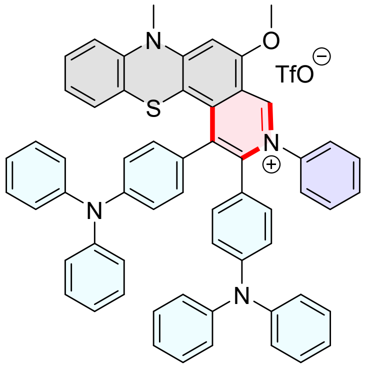


**1,2-bis(4-(diphenylamino)phenyl)-5-methoxy-7-methyl-3-phenyl-7*H*-pyrido[3,4-*c*]phenothiazin-3-ium trifluoromethanesulfonate (4f)**

Red solid 91 mg, yield: 90%. $^{1}$H NMR (400 MHz, $CDCl_3$): $\delta$ (ppm) 9.29 (s, 1H), 7.44-7.36 (m, 5H), 7.29-7.21 (m, 7H), 7.17 (d, $J$ = 8.0 Hz, 1H), 7.12 (d, $J$ = 8.0 Hz, 4H), 7.06-6.96 (m, 12H), 6.91 (d, $J$ = 8.0 Hz, 4H), 6.78-6.70 (m, 5H), 4.20 (s, 3H),

3.70 (s, 3H); $^{13}$C NMR (100 MHz, $CDCl_3$): $\delta$ (ppm) 159.87, 159.29, 148.27, 148.18, 147.54, 146.95, 145.89, 144.42, 142.82, 142.42, 135.07, 134.92, 132.56, 132.29, 130.13, 129.84, 129.66, 129.58, 129.54, 128.25, 127.27, 127.14, 125.12, 124.93, 124.60, 124.52, 124.18, 123.90, 123.40, 122.93, 121.25, 116.93, 115.92, 108.65, 100.54, 56.73, 38.09. HRMS [ESI$^+$]: calcd for $C_{59}H_{45}N_4^+OS$, 857.3309, found 857.3324.

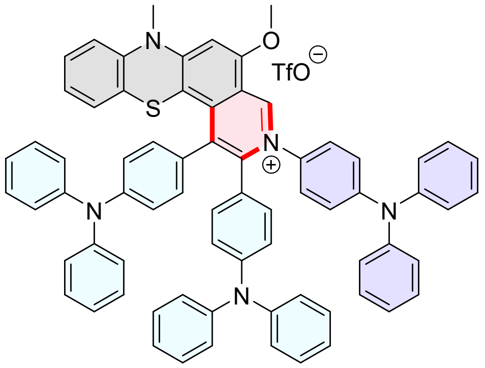


**7-1,2,3-tris(4-(diphenylamino)phenyl)-5-methoxy-7-methyl-7*H*-pyrido[3,4-*c*]phenothiazin-3-ium trifluoromethanesulfonate (4g)**

Red solid 100 mg, yield: 85%. $^{1}$H NMR (400 MHz, $CDCl_3$): $\delta$ (ppm) 9.32 (s, 1H), 7.30-7.25 (m, 13H), 7.14-7.07 (m, 17H), 7.00-6.96 (m, 12H), 6.81-6.75 (m, 5H), 4.23 (s, 3H), 3.70 (s, 3H); $^{13}$C NMR (100 MHz, $CDCl_3$): $\delta$ (ppm) 159.78, 159.16, 149.41, 148.26, 148.13, 147.54, 147.04, 146.71, 146.14, 144.50, 142.95, 135.10, 134.96, 134.85, 132.56, 132.31, 129.86, 129.72, 129.55, 128.25, 127.69, 127.24, 125.50, 125.06, 124.72, 124.62, 124.02, 123.41, 122.91, 121.43, 121.03, 117.02, 115.91, 108.58, 100.58, 57.71, 38.09. HRMS [ESI$^+$]: calcd for $C_{71}H_{54}N_5^+OS$, 1024.4044, found 1024.4048.

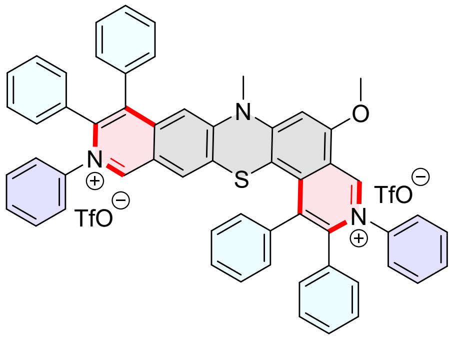


**5-methoxy-7-methyl-1,2,3,9,10,11-hexaphenyl-7*H*-dipyrido[4,3-*b*:4',3'-*h*]phenothiazine-3,11-diium trifluoromethanesulfonate (4h)**

A 10 mL reaction tube with a magnetic stir bar was charged with **1b** (0.05 mmol), **2a** (0.15 mmol, 1.5 equiv), **3a** (0.15 mmol, 1.5 equiv), $[RuCl_2(p\text{-cymene})]_2$ (3.1 mg, 10 mol%), $Cu(OAc)_2$ (36.2 mg, 0.2 mmol), TfOH (8.8 μL, 0.1 mmol) and DCE (1 mL) under air atmosphere. The resulting mixture was stirred at 140 ºC for 12 h. The reaction mixture was cooled to ambient temperature, and diluted with 5 mL of DCM. The mixture was filtered through a celite pad and washed with 20 mL of DCM. The organic phase was evaporated under a vacuum. The residue was purified by column chromatography (DCM/MeOH = 20/1, v/v) on silica gel to obtain products **4h**. Red solid 35 mg, yield: 64%. $^1$H NMR (400 MHz, $CDCl_3$): $\delta$ (ppm) 9.46 (s, 1H), 9.22 (s, 1H), 7.62 (s, 1H), 7.44-7.42 (m, 4H), 7.35-7.32 (m, 10H), 7.28-7.25 (m, 4H), 7.08-6.96 (m, 13H), 6.86 (s, 1H), 4.19 (s, 3H), 3.54 (s, 3H); $^{13}$C NMR (100 MHz, $CDCl_3$): $\delta$ (ppm) 159.78, 154.60, 152.97, 147.00, 146.54, 144.85, 144.08, 142.12, 142.07, 140.48, 137.63, 136.22, 135.46, 134.96, 133.08, 132.48, 131.70, 131.24, 131.14, 130.46, 130.34, 130.29, 129.62, 129.22, 129.14, 128.98, 128.87, 128.50, 127.99,

127.81, 127.36, 126.91, 126.83, 124.68, 122.33, 119.14, 117.96, 109.64, 108.51, 102.32, 57.58, 38.53. HRMS [ESI$^+$]: calcd for $C_{56}H_{41}N_3^{2+}OS$, 401.6480, found 401.6482.

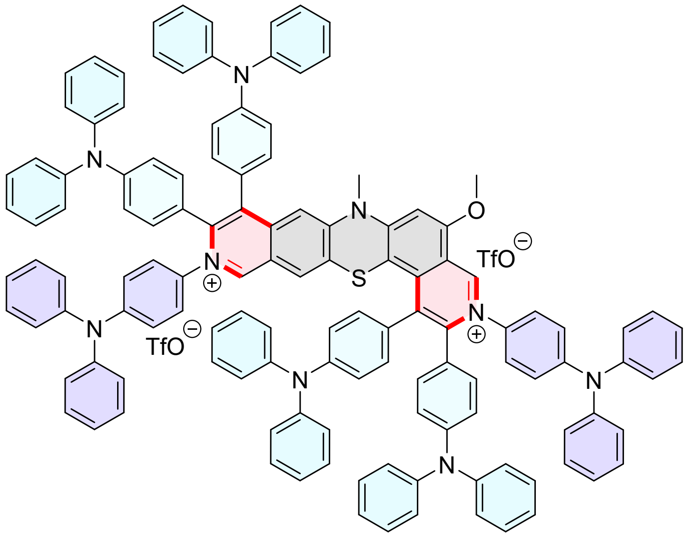


**1,2,3,9,10,11-hexakis(4-(diphenylamino)phenyl)-5-methoxy-7-methyl-7*H*-dipyrido[4,3-*b*:4',3'-*h*]phenothiazine-3,11-diium trifluoromethanesulfonate (4i)**

A 10 mL reaction tube with a magnetic stir bar was charged with **1b** (0.05 mmol), $N^1$, $N^1$-diphenylbenzene-1,4-diamine (0.15 mmol, 1.5 equiv), 4,4'-(ethyne-1,2-diyl)bis(*N,N*-diphenylaniline) (0.15 mmol, 1.5 equiv), $[RuCl_2(p\text{-cymene})]_2$ (6.2 mg, 20 mol%), $Cu(OAc)_2$ (36.2 mg, 0.2 mmol), TfOH (8.8 μL, 0.1 mmol) and DCE (1 mL) under air atmosphere. The resulting mixture was stirred at 140 ºC for 24 h. The reaction mixture was cooled to ambient temperature, and diluted with 5 mL of DCM. The mixture was filtered through a celite pad and washed with 20 mL of DCM. The organic phase was evaporated under a vacuum. The residue was purified by column chromatography (DCM/MeOH = 20/1, v/v) on silica gel to obtain products **4i**. Red

solid 40 mg, yield: 38%. $^{1}H$ NMR (400 MHz, $CDCl_3$): $\delta$ (ppm) 9.46 (s, 1H), 9.22 (s, 1H), 7.78 (s, 1H), 7.30-7.28 (m, 16H), 7.24-7.22 (m, 4H), 7.16-7.00 (m, 58H), 6.84-6.80 (m, 8H), 4.26 (s, 3H), 3.71 (s, 3H); $^{13}C$ NMR (100 MHz, $CDCl_3$): $\delta$ (ppm) 159.42, 153.64, 153.38, 149.63, 149.47, 148.81, 148.34, 147.23, 146.97, 146.72, 146.62, 145.46, 143.68, 140.45, 137.63, 136.51, 135.67, 135.25, 134.99, 132.81, 132.28, 132.21, 131.39, 129.90, 129.84, 129.75, 129.69, 128.20, 127.59, 127.29, 126.05, 125.57, 125.53, 125.28, 125.21, 125.13, 124.72, 124.56, 124.10, 123.94, 123.80, 122.40, 122.24, 121.97, 121.41, 121.25, 121.10, 121.01, 119.20, 118.16, 110.42, 108.35, 102.70, 57.70, 38.55. HRMS [ESI$^+$]: calcd for $C_{128}H_{95}N_9^{2+}OS$, 903.3702, found 903.3695.

## XVI. Crystal

Single crystal of **4g** (CCDC-2369380) was obtained by slow diffusion of petroleum ether into dichloromethane solution. These data can be obtained free of charge from The Cambridge Crystallographic Data Centre via www. ccdc.cam.ac.uk/data request/cif.

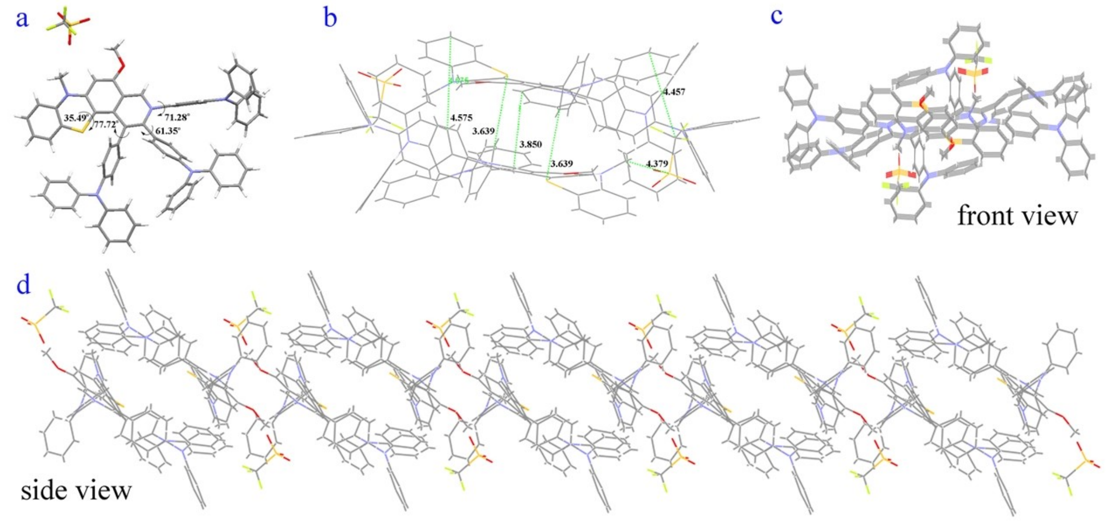

**Fig. S37** Crystal packing of **4g**.

Bond precision: C-C = 0.0049 A Wavelength=1.54184

| | | | |
|---|---|---|---|
| Cell: | a=11.8230(3) | b=13.6657(3) | c=23.0648(6) |
| | alpha=100.582(2) | beta=94.663(2) | gamma=108.170(2) |
| Temperature: | 200 K | | |

| | Calculated | Reported |
|---|---|---|
| Volume | 3442.01(16) | 3442.01(15) |
| Space group | P -1 | P -1 |
| Hall group | -P 1 | -P 1 |
| Moiety formula | C71 H54 N5 O S, C F3 O3 S [+ solvent] | C71 H54 N5 O S, C F3 O3 S, 0.5[], 0.5[] |
| Sum formula | C72 H54 F3 N5 O4 S2 [+ solvent] | C72 H54 F3 N5 O4 S2 |
| Mr | 1174.32 | 1174.32 |
| Dx,g cm-3 | 1.133 | 1.133 |
| Z | 2 | 2 |
| Mu (mm-1) | 1.163 | 1.163 |
| F000 | 1224.0 | 1224.0 |
| F000' | 1228.90 | |
| h,k,lmax | 14,16,28 | 14,16,28 |
| Nref | 13133 | 13073 |
| Tmin,Tmax | 0.758,0.840 | 0.448,1.000 |
| Tmin' | 0.688 | |

Correction method= # Reported T Limits: Tmin=0.448 Tmax=1.000
AbsCorr = MULTI-SCAN

Data completeness= 0.995 Theta(max)= 70.293

**Fig. S38** Crystal dates of **4g.**

## XVII. References

[1] J. Zhang, T. Sun, K. Wang, R. Hu, C. Zhou, H. Ge and B. Li, *Chem. Sci.* **2024**, *15*, 12270–12276.

[2] M. J. Frisch, G. W. Trucks, H. B. Schlegel, G. E. Scuseria, M. A. Robb, J. R. Cheeseman, G. Scalmani, V. Barone, G. A. Petersson, H. Nakatsuji, X. Li, M. Caricato, A. V. Marenich, J. Bloino, B. G. Janesko, R. Gomperts, B. Mennucci, H. P.

Hratchian, J. V. Ortiz, A. F. Izmaylov, J. L. Sonnenberg, D. Williams-Young, F. Ding, F. Lipparini, F. Egidi, J. Goings, B. Peng, A. Petrone, T. Henderson, D. Ranasinghe, V. G. Zakrzewski, J. Gao, N. Rega, G. Zheng, W. Liang, M. Hada, M. Ehara, K. Toyota, R. Fukuda, J. Hasegawa, M. Ishida, T. Nakajima, Y. Honda, O. Kitao, H. Nakai, T. Vreven, K. Throssell, J. A. Montgomery, Jr., J. E. Peralta, F. Ogliaro, M. J. Bearpark, J. J. Heyd, E. N. Brothers, K. N. Kudin, V. N. Staroverov, T. A. Keith, R. Kobayashi, J. Normand, K. Raghavachari, A. P. Rendell, J. C. Burant, S. S. Iyengar, J. Tomasi, M. Cossi, J. M. Millam, M. Klene, C. Adamo, R. Cammi, J. W. Ochterski, R. L. Martin, K. Morokuma, O. Farkas, J. B. Foresman, and D. J. Fox, Gaussian 16, Revision A.03, Gaussian, Inc., Wallingford CT, 2016.

[3] T. Lu, and F. Chen, *J. Comput. Chem.* **2012**, *33*, 580-592.

[4] T. Le Bahers, C. Adamo, and I. Ciofini, *J. Chem. Theory Comput.* **2011**, *7*, 2498-2506.

[5] F. J. Avila Ferrer, and F. Santoro, *Phys. Chem. Chem. Phys.* **2012**, *14*, 13549-13563.

[6] D. R. Salahub, and M. C. Zerner, *ChemInform* **1990**, *21*.

[7] P. Hohenberg, and W. Kohn, *Physical Review* **1964**, *136*, B864-B871.

[8] W. Kohn, and L. J. Sham, *Physical Review* **1965**, *140*, A1133-A1138.

[9] M. E. Casida, in, WORLD SCIENTIFIC, 1995, vol. Volume 1,pp. 155-192.

[10] E. Runge, and E. K. U. Gross, *Phys. Rev. Lett.* **1984**, *52*, 997-1000.

[11] T. Yanai, D. P. Tew, and N. C. Handy, *Chem. Phys. Lett.* **2004**, *393*, 51-57.

[12] K. Veys, and D. Escudero, *Acc. Chem. Res*. **2022**, *55*, 2698−2707.

[13] K. Veys, and D. Escudero, *J. Phys. Chem. A* **2020**, *124*, 7228−7237.

[14] M. Röhrs, and D. Escudero, *J. Phys. Chem. Lett.* **2019**, *10*, 5798−5804.

[15] K. Veys, F. d. Jong, A. Adriaens, G. Coenen, K. Coenen, Y. D. Ligt, Q. Meysman, T. Paredis, J. Stalmans,. Dehaen, M. V. d. Auweraer, and D. Escudero, *ChemPhotoChem* **2023**, *7*, e202200262.

# XVIII. Copies of $^{1}H$ and $^{13}C$ NMR spectra

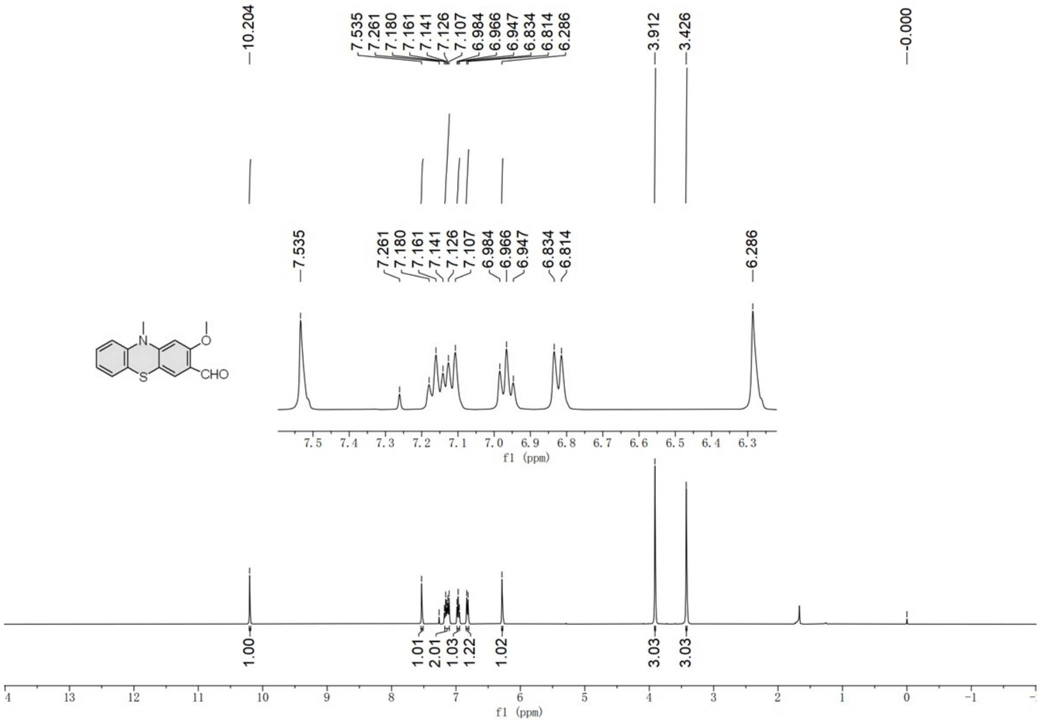


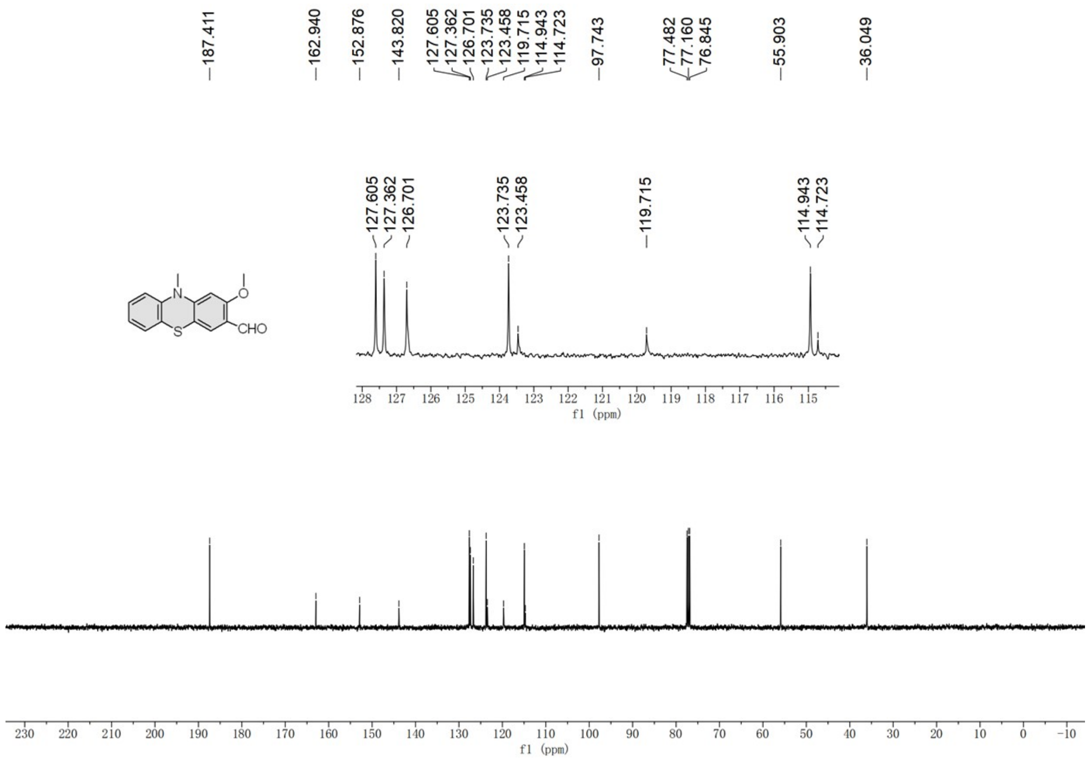

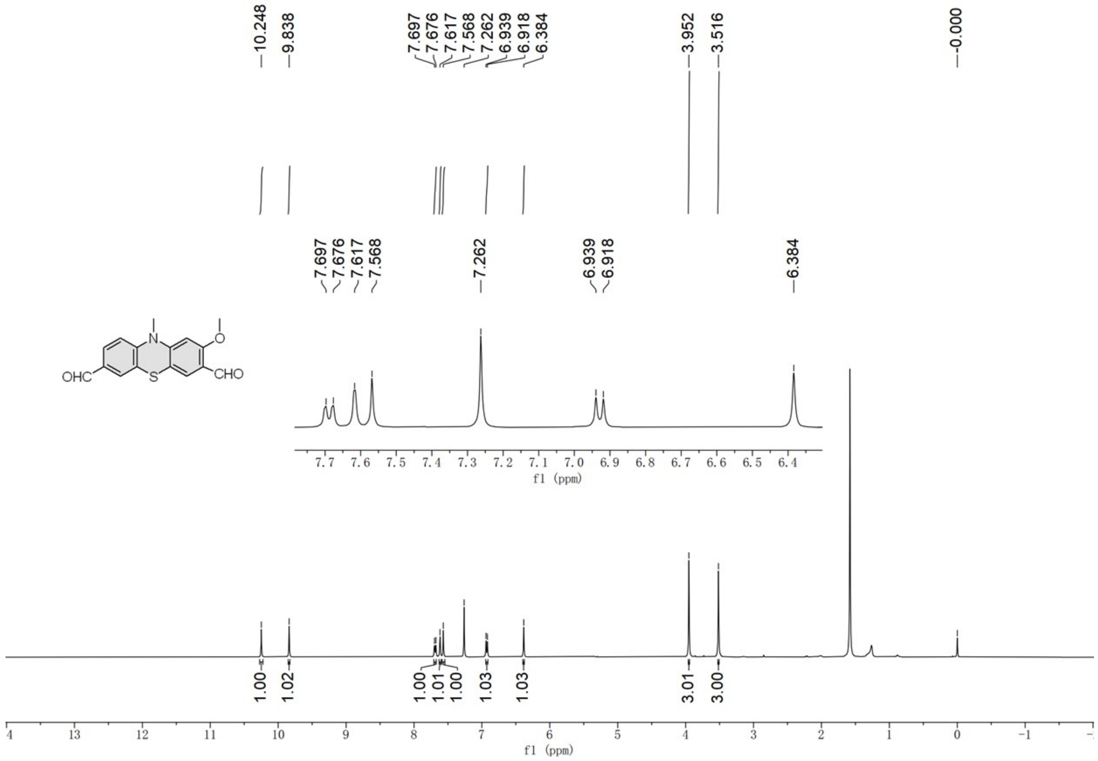
10.248
9.838
7.697
7.676
7.617
7.568
7.262
6.939
6.918
6.384
3.952
3.516
0.000
OHC
N
O
S
CHO
1.00
1.02
1.00
1.01
1.00
1.03
1.03
3.01
3.00
f1 (ppm)

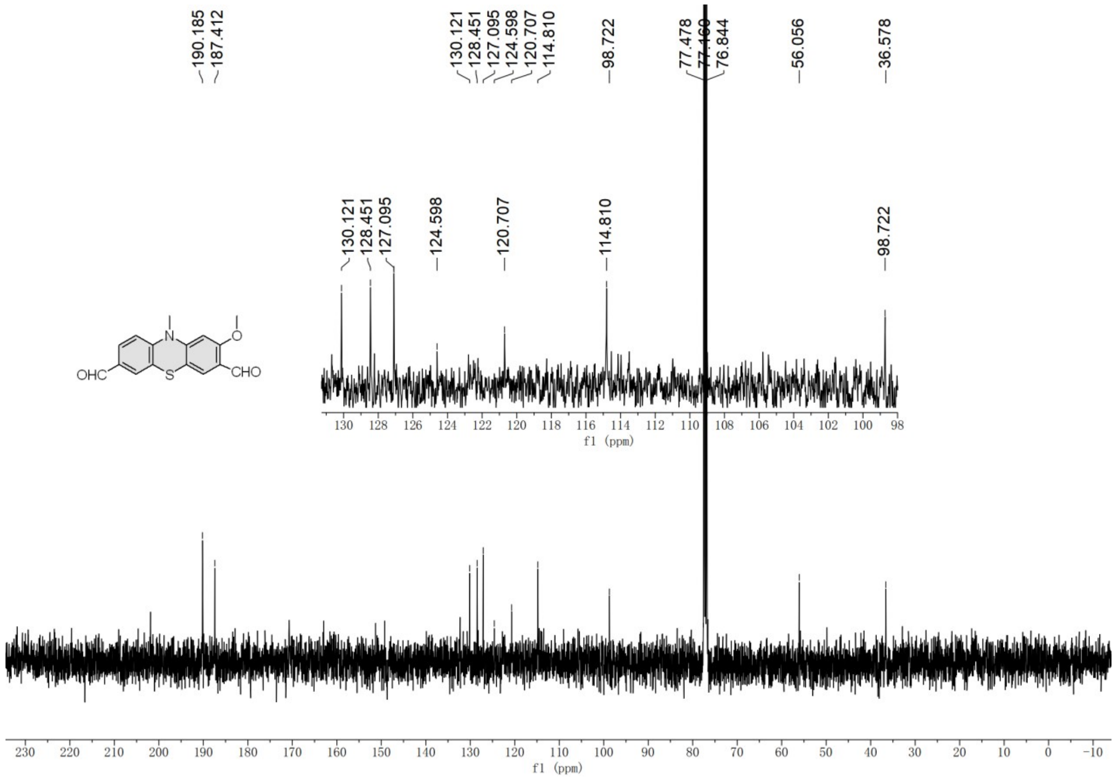
190.185
187.412
130.121
128.451
127.095
124.598
120.707
114.810
98.722
77.478
77.160
76.844
56.056
36.578
OHC
N
O
S
CHO
f1 (ppm)

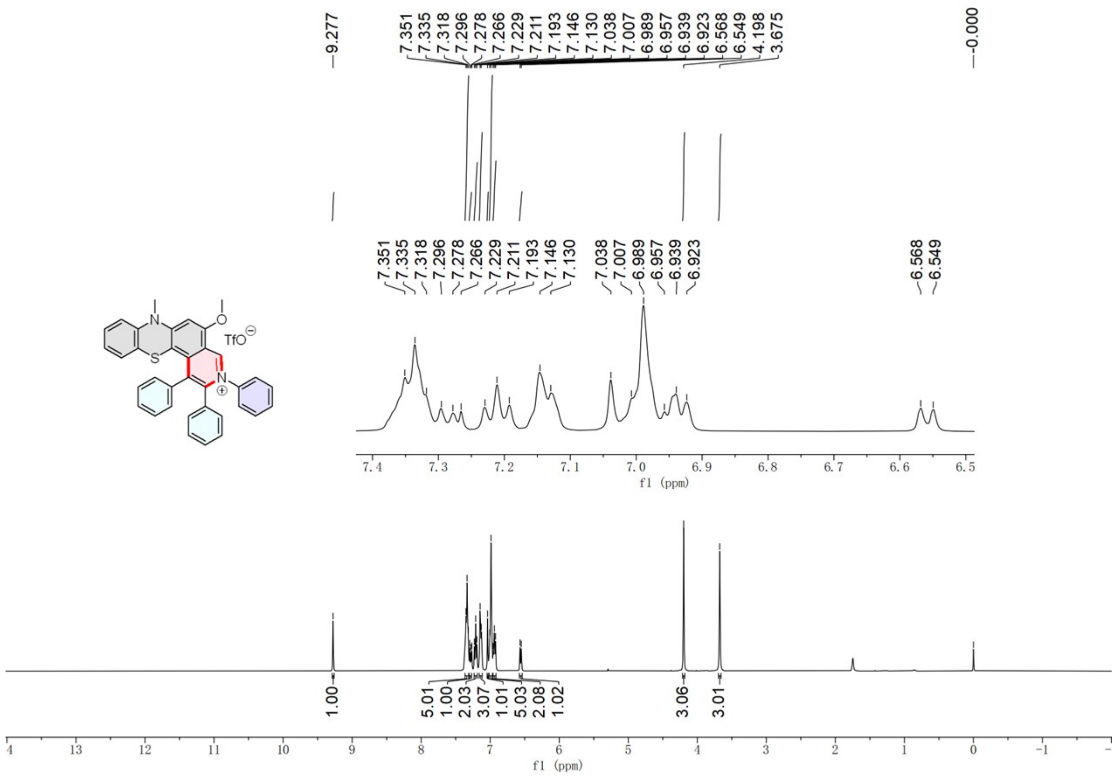

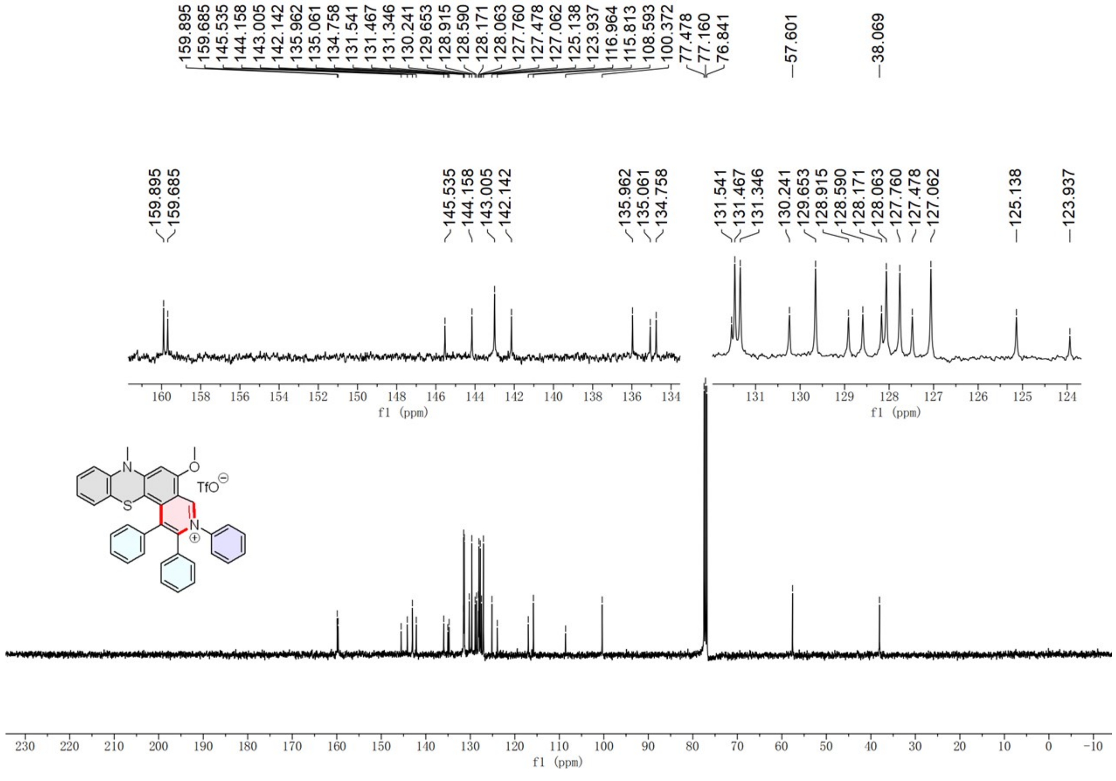

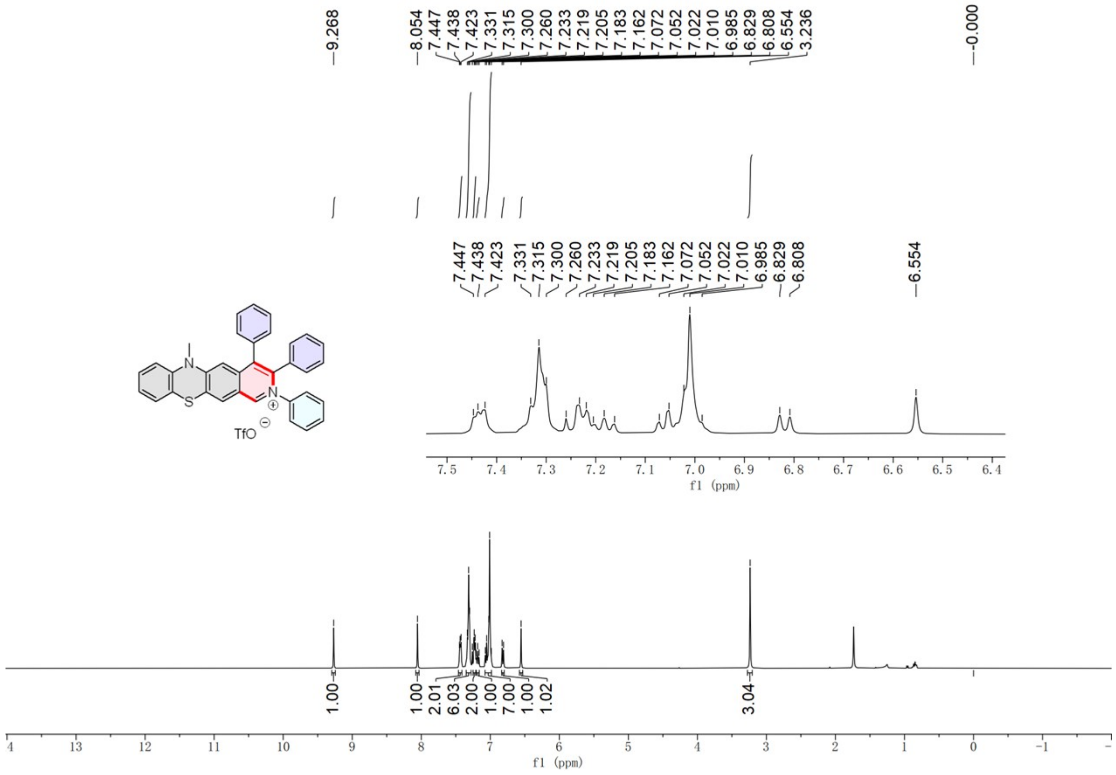

9.268
8.054 7.447 7.438 7.423 7.331 7.315 7.300 7.260 7.233 7.219 7.205 7.183 7.162 7.072 7.052 7.022 7.010 6.985 6.829 6.808 6.554 3.236
0.000
7.447 7.438 7.423 7.331 7.315 7.300 7.260 7.233 7.219 7.205 7.183 7.162 7.072 7.052 7.022 7.010 6.985 6.829 6.808
6.554
N
S
N
TfO
1.00 1.00 2.01 6.03 2.00 1.00 7.00 1.00 1.02 3.04
f1 (ppm)
f1 (ppm)


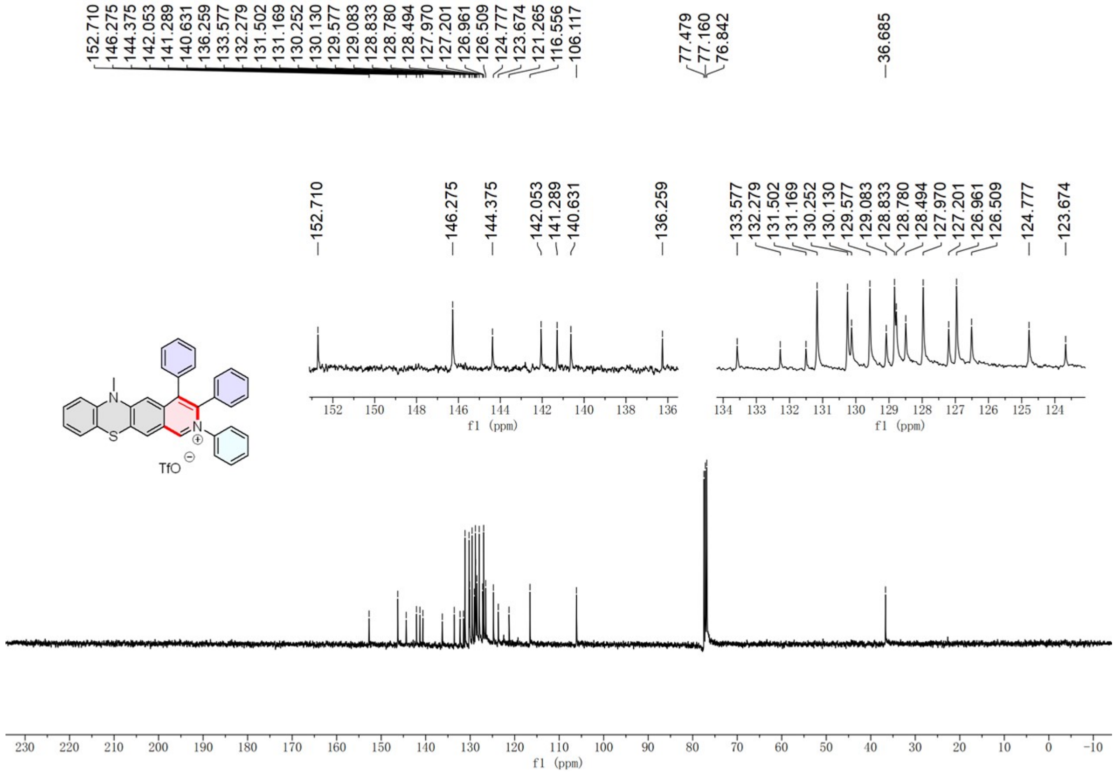

152.710 146.275 144.375 142.053 141.289 140.631 136.259 133.577 132.279 131.502 131.169 130.252 130.130 129.577 129.083 128.833 128.780 128.494 127.970 127.201 126.961 126.509 124.777 123.674 121.265 116.556 106.117
77.479 77.160 76.842
36.685
N
S
N
TfO
f1 (ppm)
f1 (ppm)
f1 (ppm)

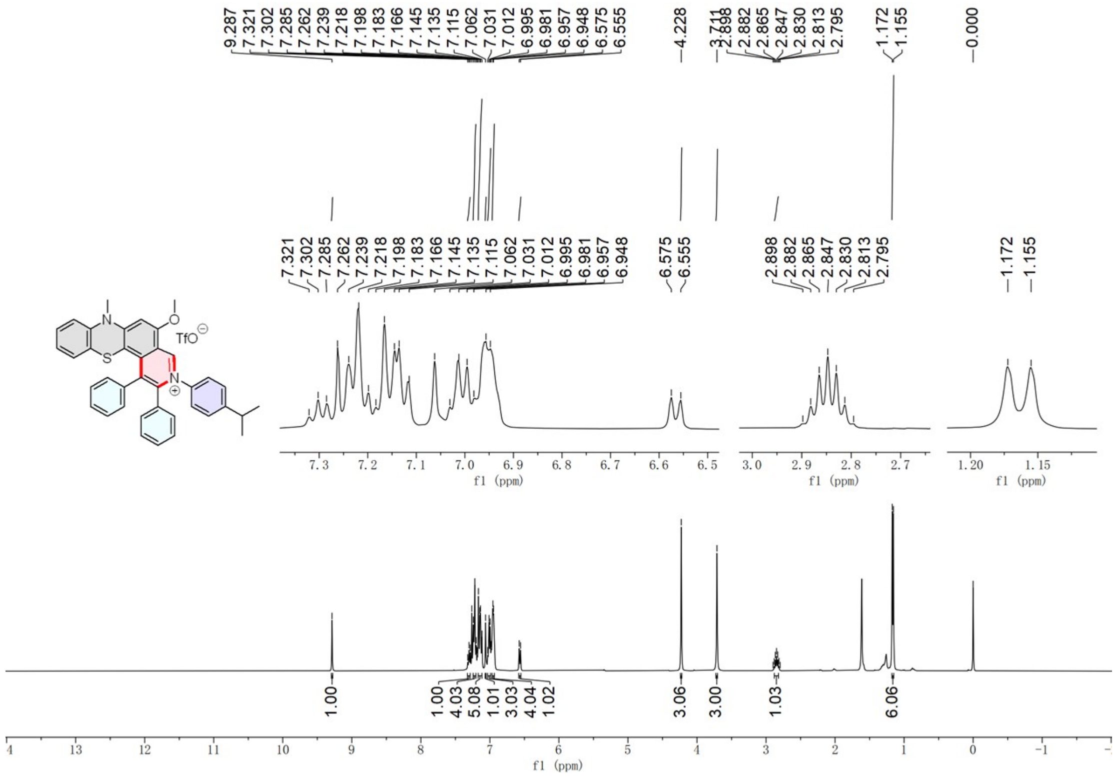
9.287 7.321 7.302 7.285 7.262 7.239 7.218 7.198 7.183 7.166 7.145 7.135 7.115 7.062 7.031 7.012 6.995 6.981 6.957 6.948 6.575 6.555
4.228
3.711 2.898 2.882 2.865 2.847 2.830 2.813 2.795
1.172 1.155
-0.000
N
S
TfO⊖
N⊕
1.00
1.00 4.03 5.08 1.01 3.03 4.04 1.02
3.06
3.00
1.03
6.06
f1 (ppm)

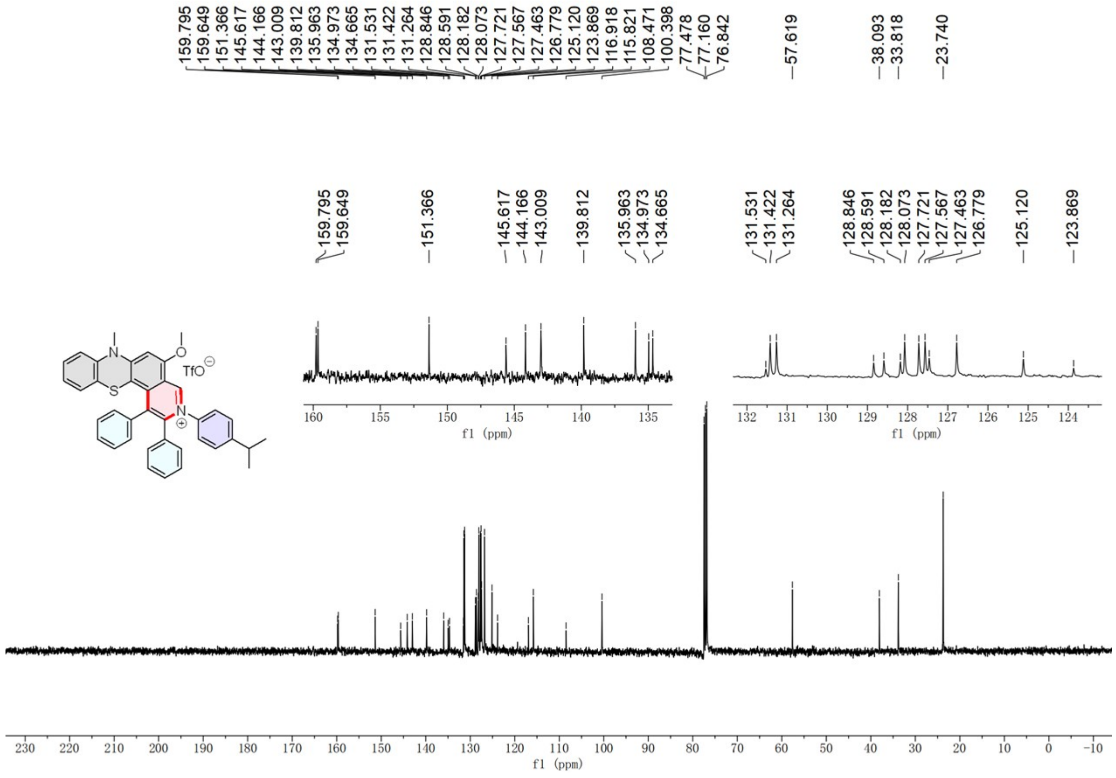
159.795 159.649 151.366 145.617 144.166 143.009 139.812 135.963 134.973 134.665 131.531 131.422 131.264 128.846 128.591 128.182 128.073 127.721 127.567 127.463 126.779 125.120 123.869 116.918 115.821 108.471 100.398 77.478 77.160 76.842
57.619
38.093 33.818
23.740
N
S
TfO⊖
N⊕
f1 (ppm)

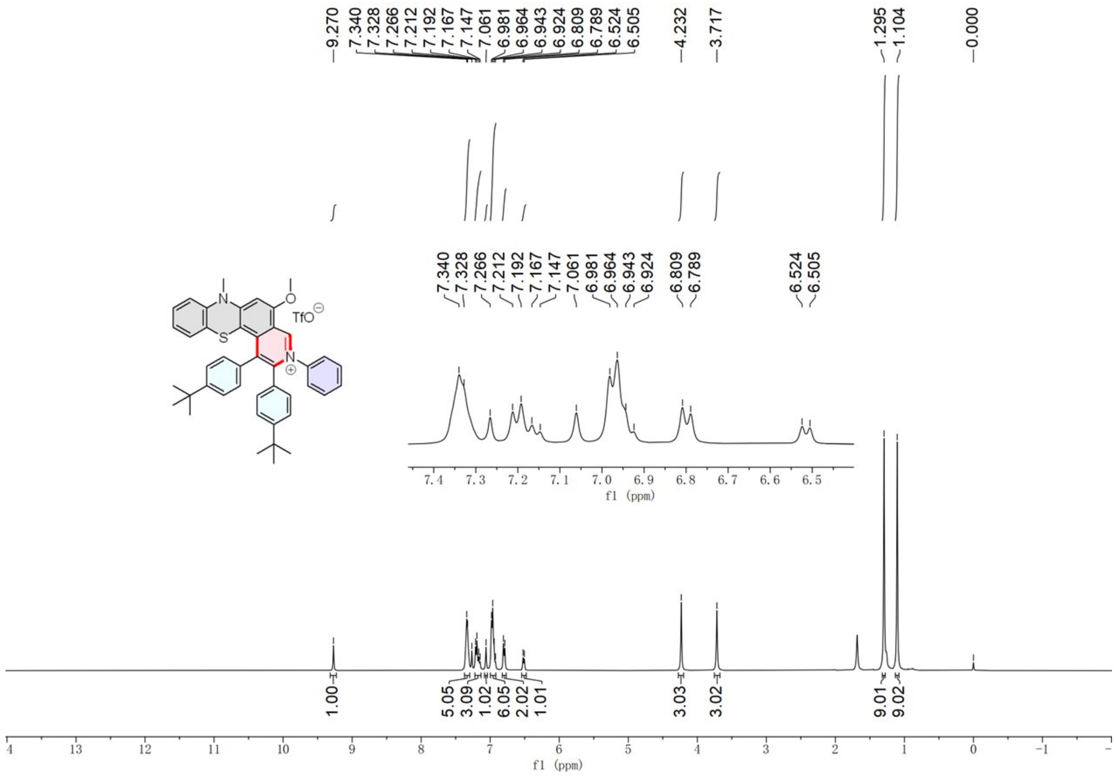
9.270
7.340 7.328 7.266 7.212 7.192 7.167 7.147 7.061 6.981 6.964 6.943 6.924 6.809 6.789 6.524 6.505
4.232
3.717
1.295 1.104
0.000
TfO⊖
N
S
1.00
5.05 3.09 1.02 6.05 2.02 1.01
3.03 3.02
9.01 9.02
f1 (ppm)

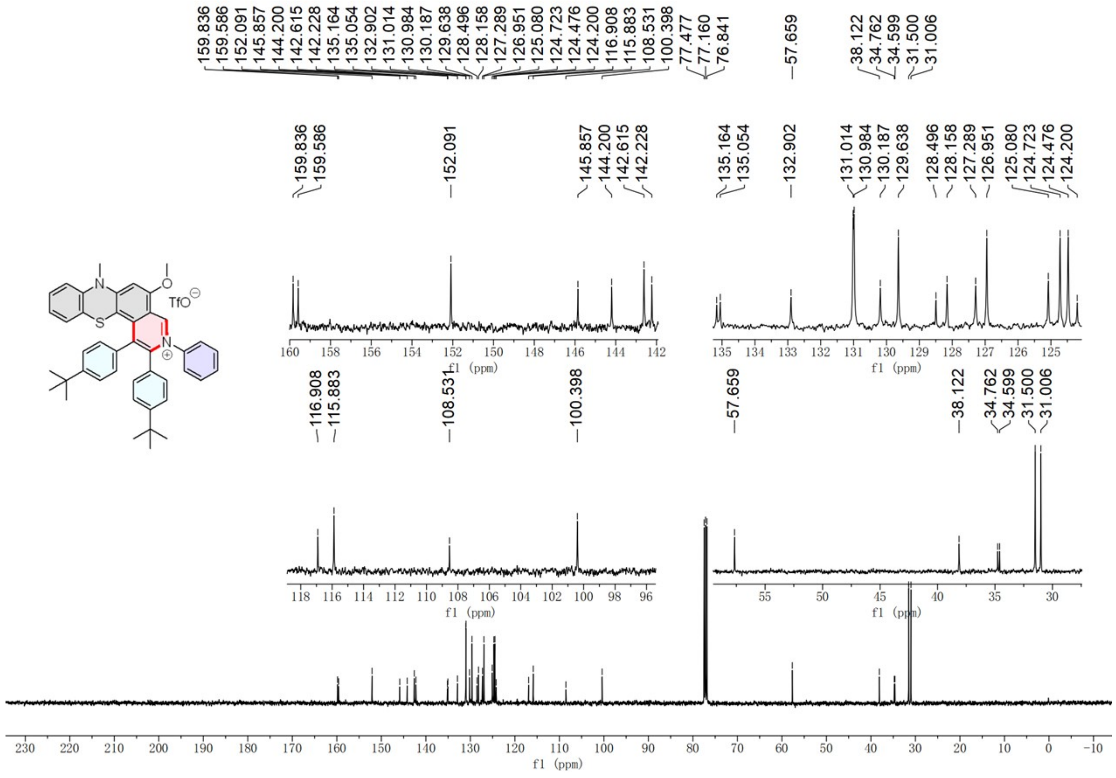
159.836 159.586 152.091 145.857 144.200 142.615 142.228 135.164 135.054 132.902 131.014 130.984 130.187 129.638 128.496 128.158 127.289 126.951 125.080 124.723 124.476 124.200 116.908 115.883 108.531 100.398 77.477 77.160 76.841
57.659
38.122 34.762 34.599 31.500 31.006
TfO⊖
N
S
f1 (ppm)

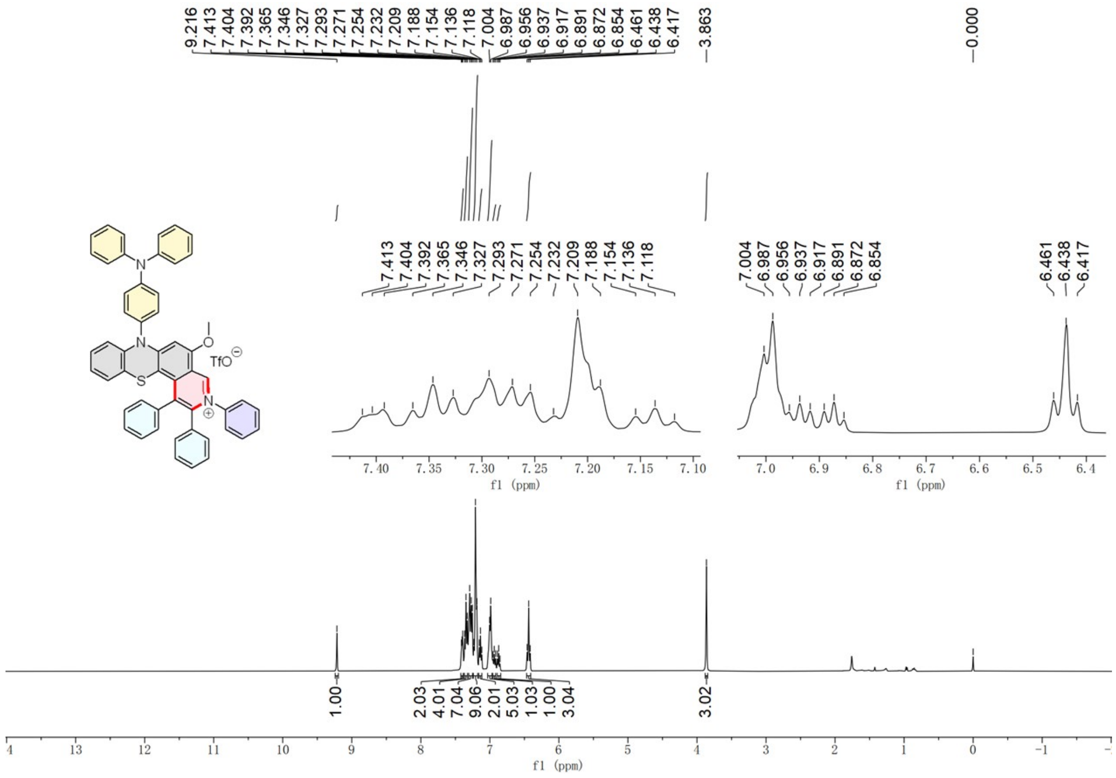
9.216 7.413 7.404 7.392 7.365 7.346 7.327 7.293 7.271 7.254 7.232 7.209 7.188 7.154 7.136 7.118 7.004 6.987 6.956 6.937 6.917 6.891 6.872 6.854 6.461 6.438 6.417
3.863
0.000
TfO
N
S
1.00 2.03 4.01 7.04 9.06 2.01 5.03 1.03 1.00 3.04 3.02
f1 (ppm)

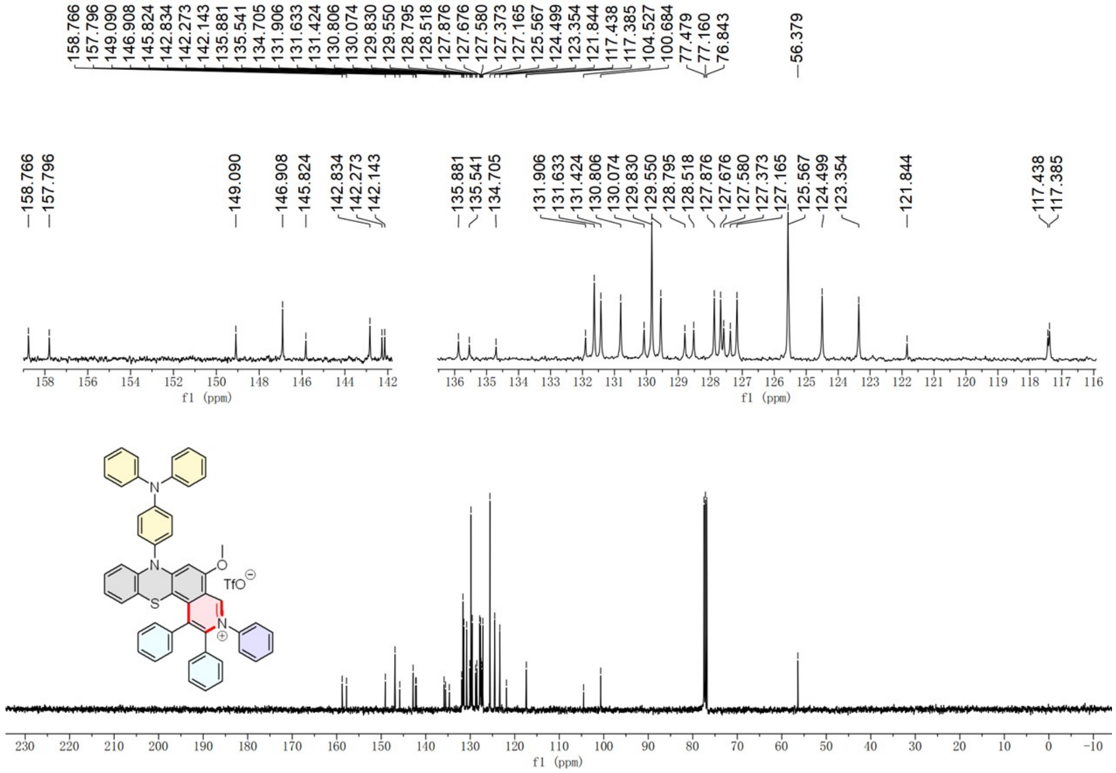
158.766 157.796 149.090 146.908 145.824 142.834 142.273 142.143 135.881 135.541 134.705 131.906 131.633 131.424 130.806 130.074 129.830 129.550 128.795 128.518 127.876 127.676 127.580 127.373 127.165 125.567 124.499 123.354 121.844 117.438 117.385 104.527 100.684 77.479 77.160 76.843
56.379
TfO
N
S
f1 (ppm)

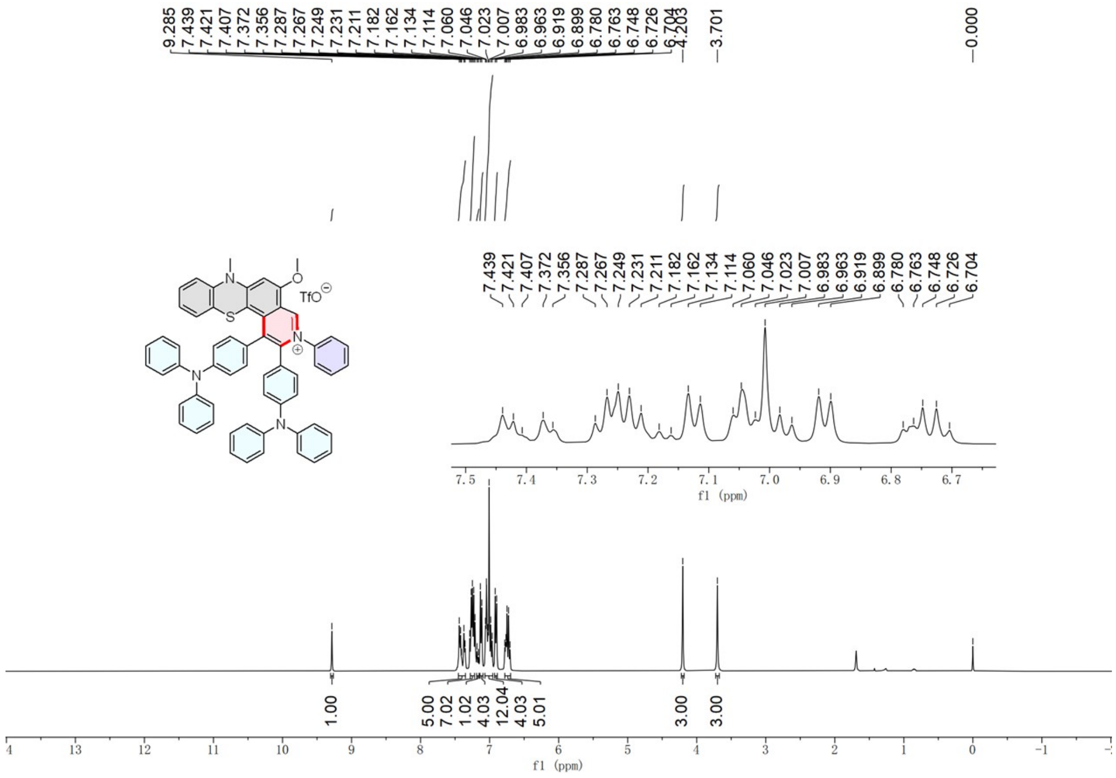

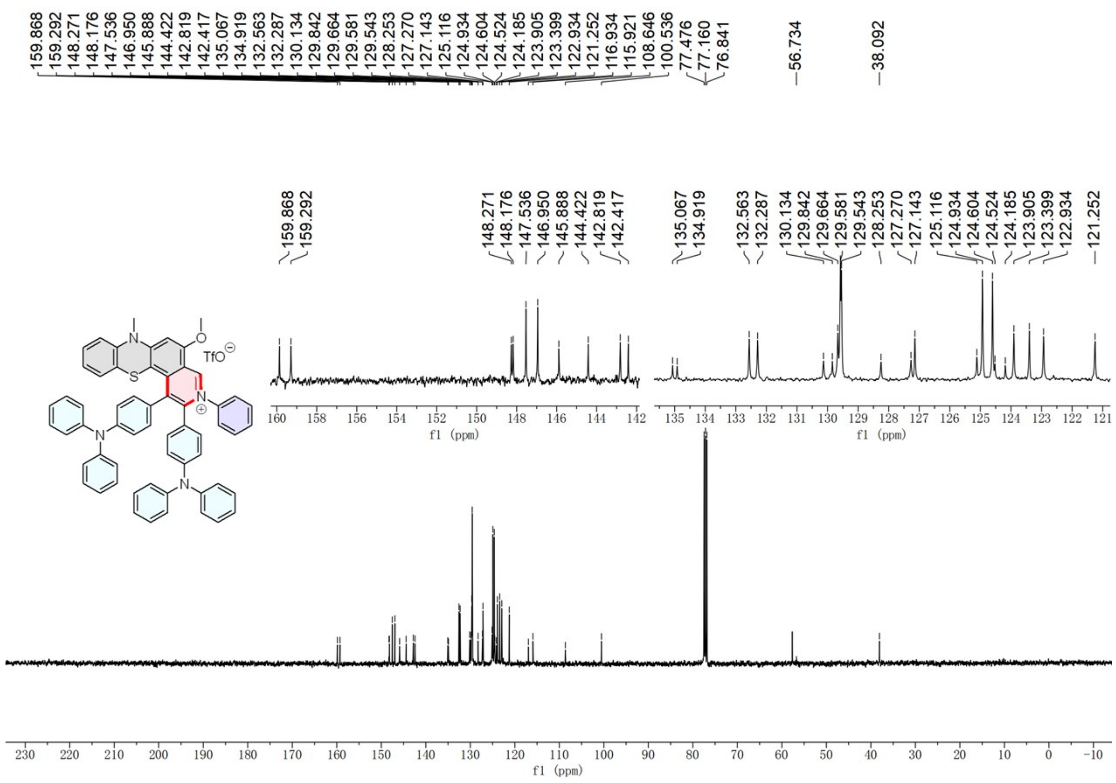

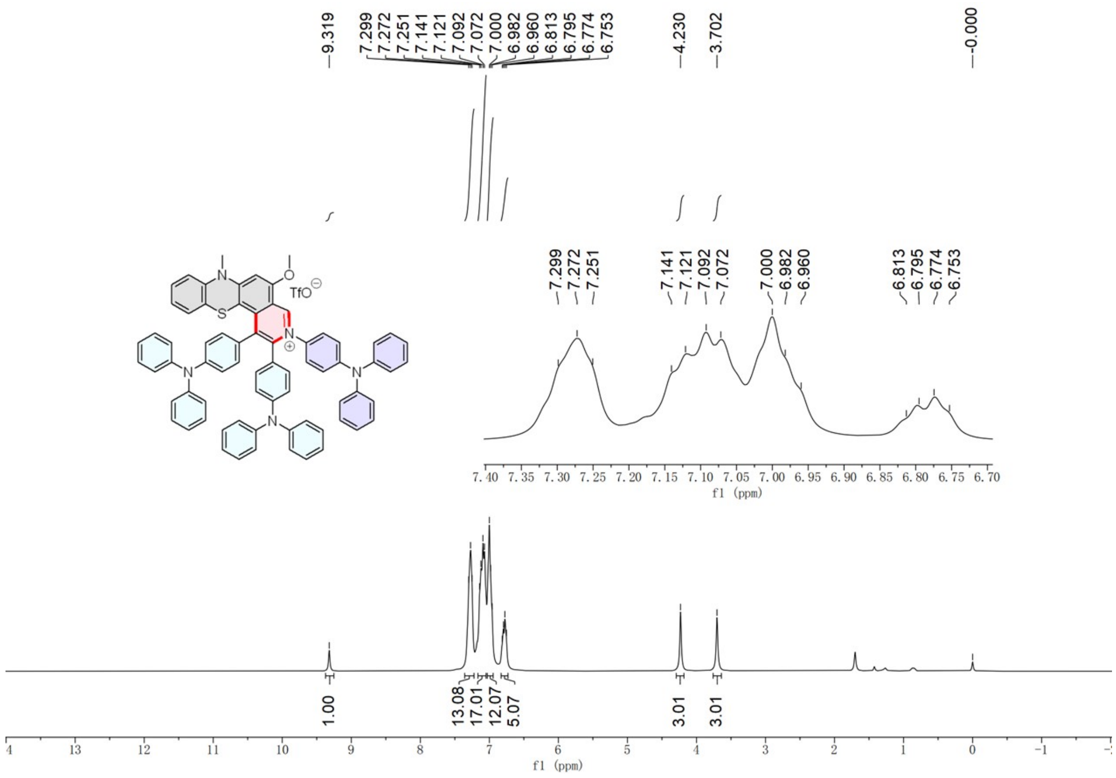
9.319
7.299
7.272
7.251
7.141
7.121
7.092
7.072
7.000
6.982
6.960
6.813
6.795
6.774
6.753
4.230
3.702
-0.000
TfO
1.00
13.08
17.01
12.07
5.07
3.01
3.01
f1 (ppm)

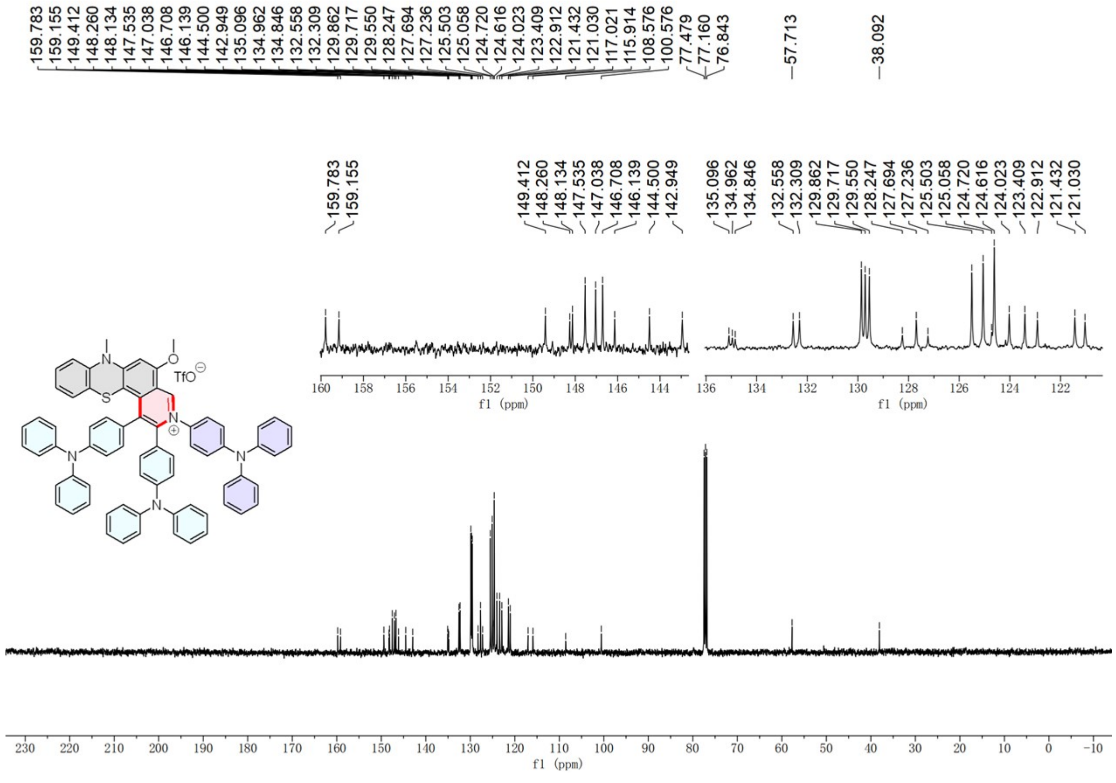
159.783
159.155
149.412
148.260
148.134
147.535
147.038
146.708
146.139
144.500
142.949
135.096
134.962
134.846
132.558
132.309
129.862
129.717
129.550
128.247
127.694
127.236
125.503
125.058
124.720
124.616
124.023
123.409
122.912
121.432
121.030
117.021
115.914
108.576
100.576
77.479
77.160
76.843
57.713
38.092
TfO
f1 (ppm)

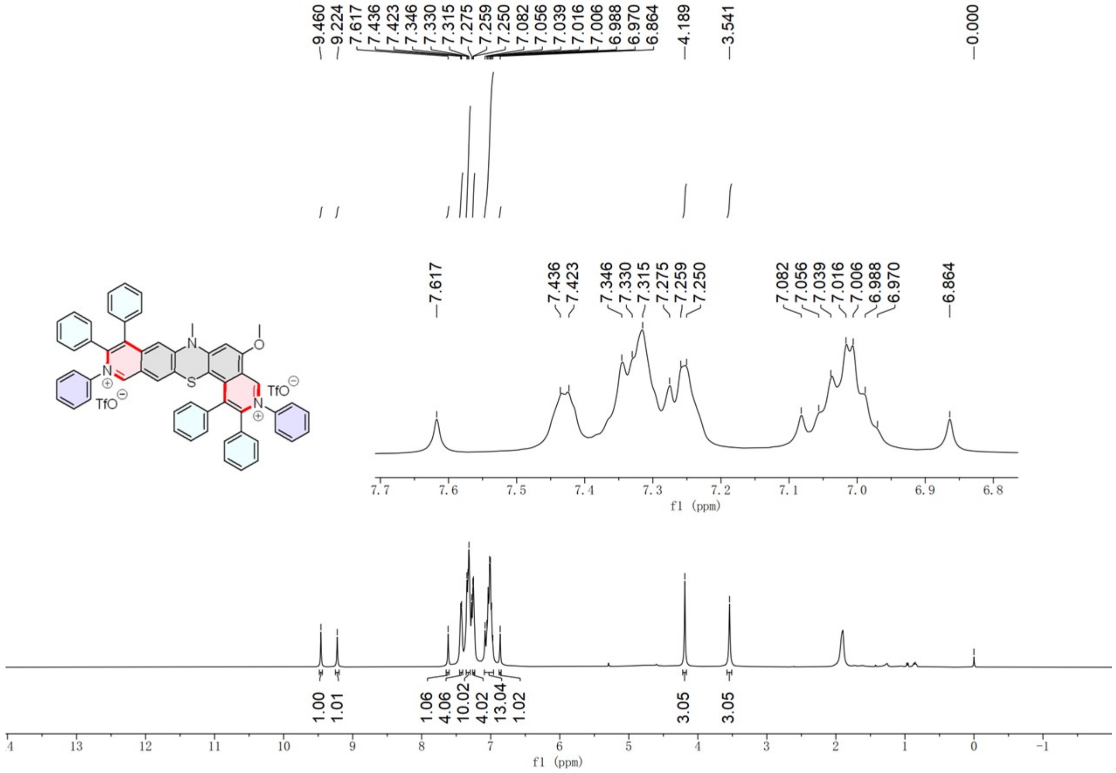
9.460
9.224
7.617
7.436
7.423
7.346
7.330
7.315
7.275
7.259
7.250
7.082
7.056
7.039
7.016
7.006
6.988
6.970
6.864
4.189
3.541
0.000
TfO
N
S
1.00
1.01
1.06
4.06
10.02
4.02
13.04
1.02
3.05
3.05
f1 (ppm)

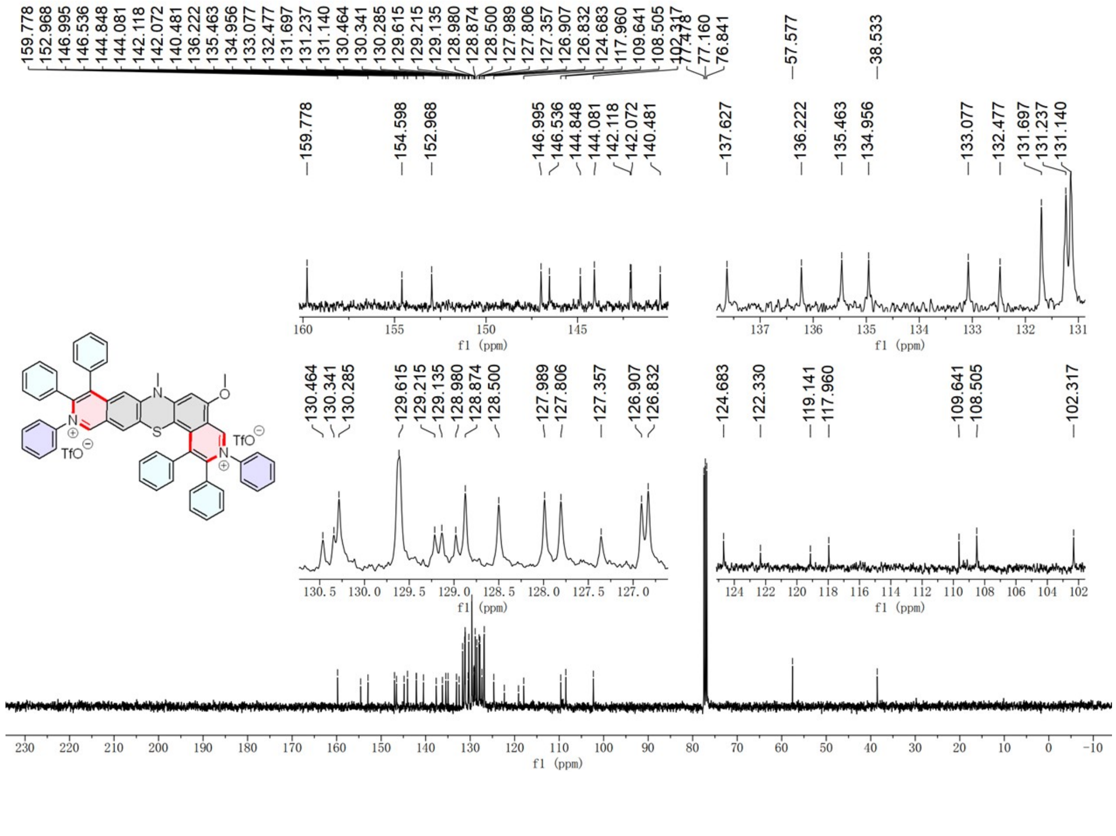
159.778
152.968
146.995
146.536
144.848
144.081
142.118
142.072
140.481
136.222
135.463
134.956
133.077
132.477
131.697
131.237
131.140
130.464
130.341
130.285
129.615
129.215
129.135
128.980
128.874
128.500
127.989
127.806
127.357
126.907
126.832
124.683
117.960
109.641
108.505
102.317
77.478
77.160
76.841
57.577
38.533
154.598
137.627
122.330
119.141
TfO
N
S
f1 (ppm)

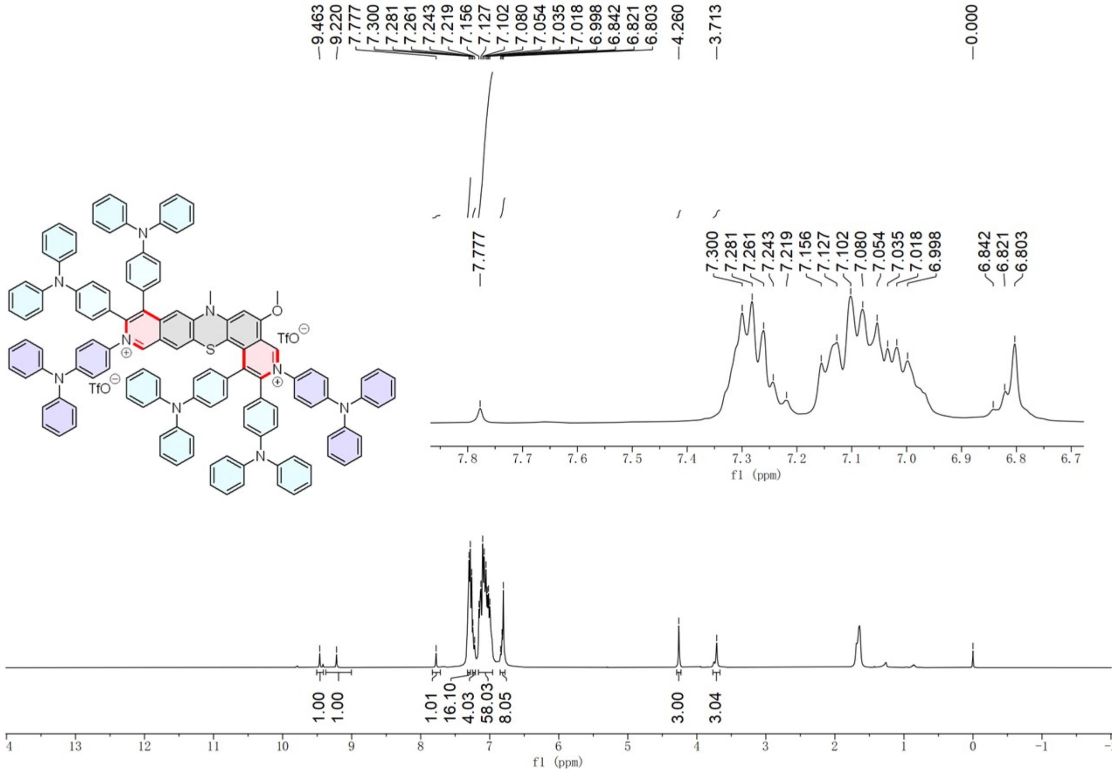
9.463
9.220
7.777
7.300
7.281
7.261
7.243
7.219
7.156
7.127
7.102
7.080
7.054
7.035
7.018
6.998
6.842
6.821
6.803
4.260
3.713
0.000
TfO⊖
N
S
1.00
1.00
1.01
16.10
4.03
58.03
8.05
3.00
3.04
f1 (ppm)

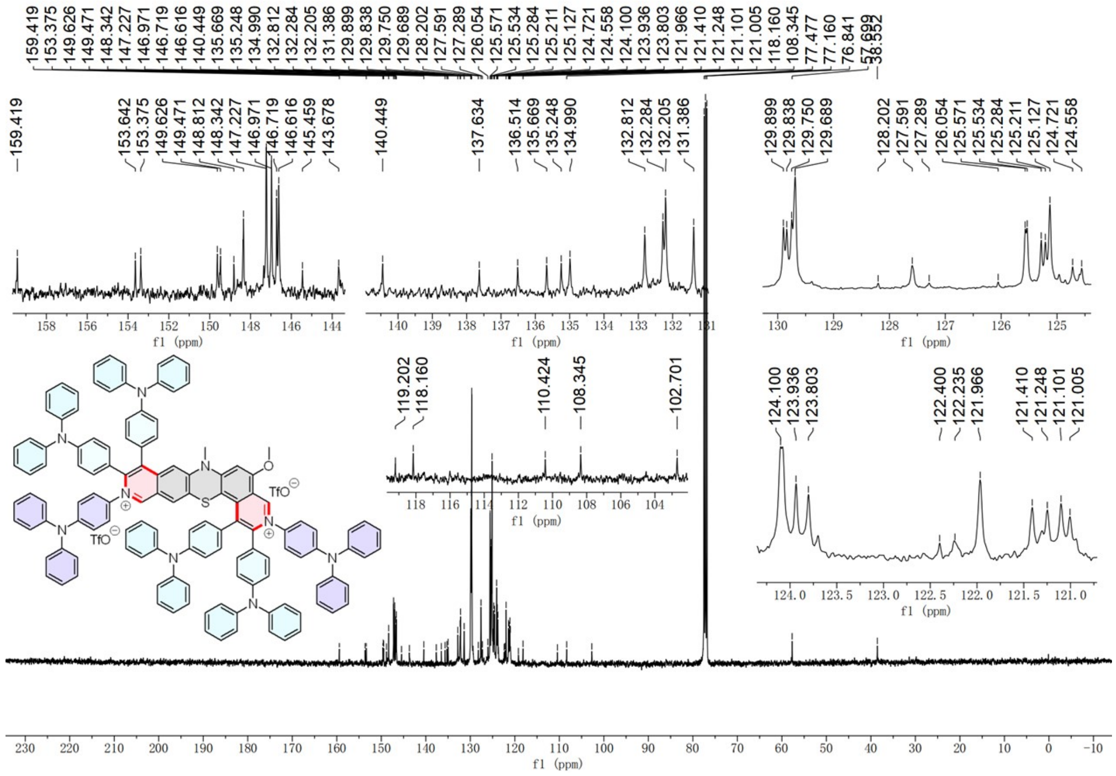
159.419
153.642
153.375
149.626
149.471
148.812
148.342
147.227
146.971
146.719
146.616
145.459
143.678
140.449
137.634
136.514
135.669
135.248
134.990
132.812
132.284
132.205
131.386
129.899
129.838
129.750
129.689
128.202
127.591
127.289
126.054
125.571
125.534
125.284
125.211
125.127
124.721
124.558
124.100
123.936
123.803
122.400
122.235
121.966
121.410
121.248
121.101
121.005
119.202
118.160
110.424
108.345
102.701
77.477
77.160
76.841
57.699
38.552
TfO⊖
N
S
f1 (ppm)
